# Comprehensive Analysis of Relative Pressure Estimation Methods Utilizing 4D Flow MRI

**Published:** 2025-03-04

**Authors:** Brandon Hardy, Judith Zimmermann, Vincent Lechner, Mia Bonini, Julio A. Sotelo, Nicholas S. Burris, Daniel B. Ennis, David Marlevi, David A. Nordsletten

**Affiliations:** Department of Biomedical Engineering, University of Michigan, Ann Arbor, USA; Department of Radiology, Stanford University, Stanford, USA; Departamento de Informática, Universidad Técnica Federico Santa María, Santiago, CHL; Department of Radiology, University of Michigan, Ann Arbor, USA; Department of Radiology, University of Wisconsin–Madison, Madison, USA; Department of Radiology, Stanford University, Stanford, USA; Department of Molecular Medicine and Surgery, Karolinska Institutet, Stockholm, SWE; Institute for Medical Engineering & Science, Massachusetts Institute of Technology, Cambridge, USA; Department of Biomedical Engineering and Cardiac Surgery, University of Michigan, Ann Arbor, USA; School of Biomedical Engineering and Imaging Sciences, King’s College London, UK

**Keywords:** Relative pressure estimation, hemodynamics, 4D flow MRI, aortic dissection

## Abstract

4D flow MRI allows for the estimation of three-dimensional relative pressure fields, providing rich pressure information, unlike catheterization and Doppler echocardiography, which provide one-dimensional pressure drops only. The accuracy of one-dimensional pressure drops derived from 4D flow has been explored in previous literature, but additional work must be done to evaluate the accuracy of three-dimensional relative pressure fields. This work presents an analysis of three state-of-the-art relative pressure estimators: virtual Work-Energy Relative Pressure (*v*WERP), the Pressure Poisson Estimator (PPE), and the Stokes Estimator (STE). Spatiotemporal behavior and sensitivity to noise were determined in silico. Estimators were validated with a type B aortic dissection (TBAD) flow phantom with varying tear geometry and an array of twelve catheter pressure measurements. Finally, the performance of each estimator was evaluated across eight patient cases. In silico pressure field errors were lower in STE compared to PPE, although PPE pressures were less affected by noise. High velocity gradients and low spatial resolution contributed most significantly to local variations in 3D error fields. Low temporal resolution leads to highly transient peak pressure events being averaged, systematically underestimating peak pressures. In the flow phantom analysis, *v*WERP was the most accurate method, followed by STE and PPE. Each pressure estimator strongly correlated with ground truth pressure values despite the tendency to underestimate peak pressures. Patient case results demonstrated that the pressure estimators could be feasibly integrated into a clinical workflow.

## I. Introduction

Relative pressure differences are important when assessing the severity of a variety of cardiovascular diseases, including aortic stenosis [1], aortic coarctation [2], mitral valve regurgitation [3], and aortic dissection [4]. Until recently, relative pressure differences could only be evaluated by invasive catheterization, which carries inherent risks as a diagnostic tool [5], or by Doppler echocardiography [6]. While Doppler echocardiography is a useful diagnostic tool in a subset of disorders, it measures only one velocity direction. It is, therefore, limited to simple pressure models, such as the simplified Bernoulli equation. 4D flow MRI [7] allows for the measurement of three-dimensional velocity fields over the cardiac cycle, capturing rich blood flow information. This rich flow information enables the estimation of additional hemodynamic variables, such as wall shear stress, kinetic energy, and relative pressure [8]–[10]. Pressure estimators that utilize 4D flow MRI have the potential to increase the diagnostic information available by mapping relative pressure across various cardiovascular domains. One-dimensional pressure drops derived from catheter measurements or Doppler echocardiography have been in use for decades, but 4D flow MRI holds the potential for utilizing three-dimensional pressure *fields* as a diagnostic tool.

We will herein focus on analyzing the performance of pressure estimators in the context of type B aortic dissection (TBAD) [11]. In brief, TBAD refers to a partial thickness entry tear in the descending aorta, creating a second pathway for blood known as the false lumen (FL). The false lumen often reconnects with the true lumen (TL) distally through an exit tear. TBAD contains a wide variety of complex flow patterns and challenging geometries, making it a valuable setting for the evaluation of pressure estimators. TBAD contains regions of low velocities, high velocities, narrow geometries, and highly transient pressure events. Low-velocity regions are helpful when analyzing sensitivity to noise. Narrow domains are essential when analyzing spatial resolution limits; highly transient events are valuable when analyzing temporal resolution limits. Finally, high spatial gradients have been reported to be particularly challenging when estimating hemodynamic parameters [12], [13], meriting further study.

Pressure estimators are highly dependent on the image quality of a given 4D flow acquisition [14]. Consequently, it is crucial to stress-test these estimators under a variety of imaging conditions. Previous work has been done to validate and explore the use of various pressure estimators [14]–[19], but additional analysis is necessary. In particular, the spatial distribution of errors requires further study. This work compares CFD-derived pressure fields with estimated pressure fields in a novel voxel-to-voxel analysis. The spatial errors resulting from velocity field corruptions are also determined. Flow phantoms with 12-point arrays of pressure catheter measurements are utilized as ground truth in an in vitro study, enhancing our understanding of the spatial variation of errors in pressure estimations derived from 4D flow acquisitions. In sum, these analyses will add to our understanding of the spatial variations in pressure estimator accuracy rather than the accuracy of one-dimensional pressure drops.

In this paper, the relative pressure estimators Virtual Work-Energy Pressure (*v*WERP), Pressure Poisson Estimator (PPE), and Stokes Estimator (STE) are analyzed across in silico, in vitro, and in vivo TBAD datasets. For the in silico analysis, we utilize patient-specific CFD simulations to generate synthetic 4D flow MRI as input. The resulting estimated relative pressure fields are compared directly to CFD-derived relative pressure fields. Spatiotemporal and noise sensitivity analyses are performed for both the pressure fields and pressure drops. For the in vitro analysis, we utilize 4D flow acquired across three TBAD flow phantom variations and an array of ground truth pressure catheter measurements [20]. Variations in the flow phantoms allow for a qualitative analysis of the changing pressure fields as the TBAD false lumen tears are modified. Validation of the pressure estimators is performed via comparison to catheter pressure measurements. Finally, pressure drops estimated in eight patient cases are compared across methods for agreement. Sources of error are identified, and each pressure estimator is compared to determine their pros and cons in different use cases.

## II. Material and Methods

### A. Relative Pressure Estimation Algorithms

Relative pressure estimation algorithms utilize full-field 4D flow MRI velocity measurements as input. These algorithms can be split into *fully spatial* and *plane-to-plane* estimators. Fully spatial estimators produce a four-dimensional (3D + time) relative pressure field and include PPE and STE. Plane-to-plane estimators produce a time-resolved relative pressure drop between two user-defined planes (e.g., the pressure drop from the aortic root to the FL exit tear over one cardiac cycle) and include *v*WERP. The pressure estimation algorithms used herein are derived from the incompressible Navier-Stokes equations [21] given by (1) and (2)

(1) $$\rho \frac{\partial u}{\partial t}+\rho (u\cdot \nabla )u=-\nabla p+\mu \text{Δ}u\;\text{on Ω}$$

(2) ∇⋅u=0on Ω,

where Ω denotes the spatial domain. Equation (1) can be rearranged to estimate pressure gradients by plugging in the measured velocity field, $u_{m}$.


(3) $$R_{m}=-\rho \left(\frac{\partial u_{m}}{\partial t}+\left(u_{m}\cdot \nabla \right)u_{m}\right)+\mu \nabla^{2}u_{m}\;\text{on Ω}$$

#### 1) Pressure Poisson Estimator (PPE):

The pressure Poisson estimator [14], [22] is derived by taking the divergence of (3), simplifying using the assumption of incompressible flow (2), and subsequently solving the resulting Poisson equation with Neumann boundary conditions.

(4) $$\nabla^{2}\hat{p}=-\rho \nabla \cdot \left(u_{m}\cdot \nabla u_{m}\right)\;\text{on Ω}$$

(5) $$\nabla \hat{p}\cdot n=-\rho \left(u_{m}\cdot \nabla u_{m}\right)\cdot n\;\text{on}\;\partial \text{Ω,}$$

where ∂Ω is the boundary of the spatial domain and $\nabla \hat{p}$ is the estimated pressure gradient field.

#### 2) Stokes Estimator (STE):

The Stokes estimator [23], [24] utilizes a divergence-free virtual field, $\tilde{u}$, as a regularizer (6).

(6) $$\nabla \hat{p}-\mu \nabla^{2}\tilde{u}=R_{m}\;\text{on Ω}$$

(7) $$\nabla \cdot \tilde{u}=0\;\text{on Ω}$$

(8) $$\tilde{u}=0\;\text{on}\;\partial \text{Ω}$$

It does not require higher-order derivatives and has been reported to handle boundaries better because it does not prescribe boundary conditions directly on the pressure field [15], as PPE does.

#### 3) Virtual Work-Energy Relative Pressure (vWERP):

Virtual work-energy relative pressure is derived from virtual work-energy equations and is based on the principle of conservation of energy. The virtual work-energy equation is given by multiplying (1) by a virtual field, w, and integrating over the domain Ω between two planes.


(9) $$\begin{array}{c} \int_{\text{Ω}}\rho \frac{\partial u_{m}}{\partial t}\cdot w\;d\text{Ω}+\int_{\text{Ω}}\rho \left(u_{m}\cdot \nabla u_{m}\right)\cdot w\;d\text{Ω}\\ -\int_{\text{Ω}}\mu \text{Δ}u_{m}\cdot w\;d\text{Ω}+\int_{\text{Ω}}\nabla p\cdot w\;d\text{Ω}=0 \end{array}$$

The virtual velocity field w is constructed by solving a Stokes flow problem over the user-specified flow domain. After integrating and rearranging (9), (the details of which can be found in [19]), the pressure drop between two planes is given by

(10) $$\text{Δ}p=\frac{-1}{Q}\left(\frac{\partial }{\partial t}K_{e}+A_{e}+V_{e}\right)$$

where $K_{e}$ denotes the kinetic energy held within the blood flow, $A_{e}$ denotes the rate of energy entering/leaving the system, and $V_{e}$ is the rate of energy dissipation due to viscous forces. Q is the volumetric flow rate between the two planes over which the pressure drop is calculated. *v*WERP has shown substantial improvement over older plane-to-plane estimators, such as the unsteady Bernoulli equation [25] and the original WERP [19].

### B. Implementation of Relative Pressure Estimation Methods

STE, PPE, and *v*WERP were all implemented using inhouse MATLAB codes. All methods utilized a midpoint temporal discretization scheme and second-order spatial derivatives where applicable. The relevant equations for STE and *v*WERP were discretized with a finite difference method (FDM) that used the measured velocity values directly as input [19]. PPE was discretized using a second-order accurate finite volume method (FVM). For PPE, an FVM was chosen over an FDM to reduce errors introduced by boundary normal vector estimation when prescribing Neumann boundary conditions (5) on the pressure field [26]. Finally, the velocity input was nearest-neighbor upsampled depending on the resolution. The 3.0, 2.0, and 1.5 mm images were upsampled by factors of 4, 4, and 2, respectively. This led to an effective resolution of 0.75, 0.50, and 0.75 mm. All velocity gradients, laplacians, and temporal derivatives were calculated using the raw velocity input before upsampling.

### C. In Silico Analysis

#### 1) Model Generation:

A patient-specific CFD model of a TBAD patient was utilized as a test bed to analyze PPE, STE, and *v*WERP. The CFD model allowed for the creation of synthetic 4D flow images at different spatiotemporal resolutions and noise levels to analyze the pressure estimation methods comprehensively. Model geometry was sourced from a clinical CT, segmented in 3D Slicer [27], and meshed with 1.1 mm elements and a 0.9 mm boundary layer in Simmetrix SimModeler [28]. Boundary conditions were prescribed using a 4D flow MRI scan (3T; voxel size = ∼1.5 mm×1.5 mm×2.5 mm; temporal resolution = 47 ms, VENC = 2.0 m/s) of the same patient. Inflow and outflow boundary conditions were imposed at the aortic root, brachiocephalic artery, left common carotid artery, left subclavian artery, and true lumen outlet based on flow rates interpolated from 4D flow to 1.0 ms resolution. Flow resistance was estimated by measuring the area of each outlet. The aortic wall was assumed to be completely rigid. A single cardiac cycle was simulated in CHeart [29]. The solved pressure field, $p_{\text{CFD}}$, was projected from the unstructured CFD mesh onto isotropic 0.50 mm and 0.75 mm grids at a 20 ms sampling rate. These projections served as the ground truth.

#### 2) Spatiotemporal Analysis:

**Fig. 1.**
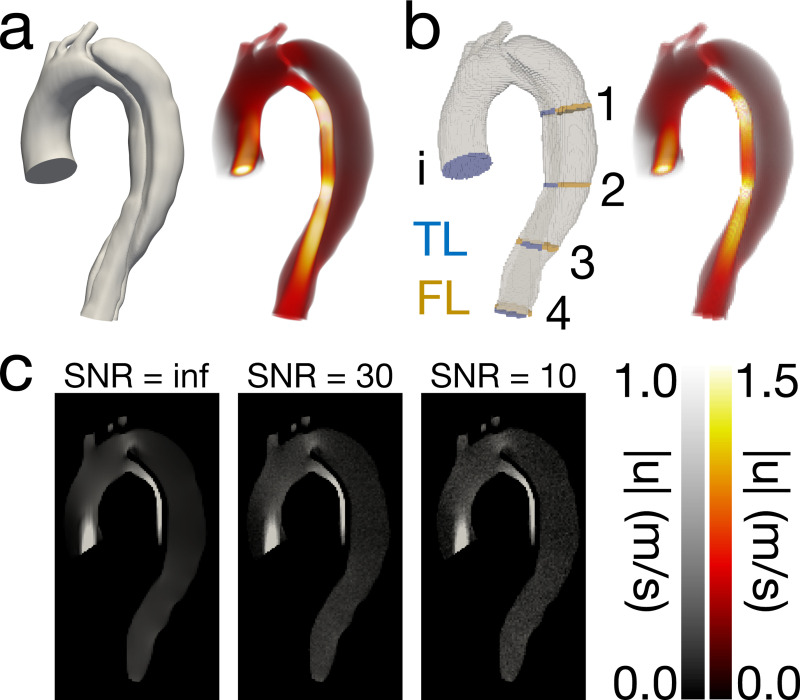
Overview of in silico model at peak systolic flow. (a) Solved velocity field on unstructured FEM mesh. (b) Solved velocity field after projection to a voxelized domain. Planes used for pressure drop analysis are shown in blue and gold. (c) Noisy velocity magnitude images at tested SNR levels. Non-fluid regions are set to zero to aid comparison.

The solved velocity field, $u_{\text{CFD}}$, was projected from the unstructured CFD mesh onto a variety of regular, isotropic grids to mimic MRI image format (Fig. 1ab). Projections were made onto 1.5, 2.0, and 3.0 mm grids at 20 ms, 40 ms, and 60 ms sampling rates for a total of nine spatiotemporal combinations. This allowed for a spatiotemporal analysis of PPE, STE, and *v*WERP to evaluate the robustness of each pressure estimation method across a variety of resolutions. Performance was analyzed by propagating each of the $u_{\text{CFD}}$ fields through each estimator and directly comparing the estimated pressure results ($p_{\text{PPE}}$, $p_{\text{STE}}$) against the ground truth pressure solution, $p_{\text{CFD}}$. Since *v*WERP is a plane-to-plane estimator, *v*WERP-derived pressure drops were compared directly with pressure drops sourced from $p_{\text{CFD}}$. Nine unique planes (for eight separate pressure drops) were generated for this purpose (Fig. 1b).

As fully spatial relative pressure estimation methods, PPE and STE could be compared directly with $p_{\text{CFD}}$. Relative error was evaluated at each timestep with (11) and across the entire time domain with (12).


(11) $$\epsilon_{\text{rel}}(t)=\frac{{\left\|p_{\text{CFD}}(t)-\hat{p}(t)\right\|}_{0,2}}{{\left\|p_{\text{CFD}}\right\|}_{0,\infty }}$$

(12) $$\varepsilon_{\text{rel}}=\frac{{\left\|p_{\text{CFD}}-\hat{p}\right\|}_{0,2}}{{\left\|p_{\text{CFD}}\right\|}_{0,2}}$$

RMSE was also calculated over the entire spatial domain at each timestep and across the entire spatiotemporal domain. In addition to these error metrics, linear regression plots were also generated.

*v*WERP pressure drop estimates were compared directly with pressure drops selected from $p_{\text{CFD}}$. Planes were drawn in ParaView [30]. Pressure drops selected from $p_{\text{STE}}$ and $p_{\text{PPE}}$ were compared with pressure drops selected from $p_{\text{CFD}}$ as well. As in the fully spatial evaluation, linear regression plots were generated.

#### 3) Noise Sensitivity Analysis:

To assess the impact of estimate accuracy, noise fields [31] were added to the projected velocity fields to evaluate the performance of PPE, STE, and *v*WERP. The noise addition methodology was as follows: Velocity fields were first converted to phase data by specifying a synthetic VENC that was 1.1 times the maximum velocity component across the entire time-resolved velocity field. Synthetic magnitude data was created by multiplying the voxelized CFD mask by a non-zero constant (leaving the non-fluid region equal to 0). The synthetic magnitude and phase images were combined to create complex images that were subsequently transformed to k-space via the fast Fourier Transform. Zero-mean, complex Gaussian noise was added to k-space. The variance of the complex Gaussian was defined to achieve a specified SNR using the equation ${\text{SNR}}_{\text{db}}=10\log\frac{P_{x}}{P_{n}}$, where $P_{x}$ is the power of the signal and $P_{n}$ is the power of the noise (which is equivalent to the variance for white Gaussian noise). Finally, the k-space data was transformed back to image space via the inverse Fourier Transform, and the phase images were converted back to velocity images using the previously specified VENC. Twenty-five realizations of SNR = 10 noise fields and SNR = 30 noise fields (Fig. 1c) were calculated for a total of 50 realizations per resolution, resulting in 450 realizations in total. The SNR = inf was performed implicitly during the spatiotemporal analysis. The same error metrics used in the previous spatiotemporal analysis were used for the noise sensitivity analysis, with some modifications to the correlation analysis — instead of performing a linear regression over one pressure field, linear regressions were performed over all 25 noise realizations at once. Finally, error averages, minimums, and maximums were calculated for each spatiotemporal and noise combination (e.g., the average error was calculated over the 25 realizations of the 1.5 mm × 40 ms and SNR = 30 combination). Finally, pressure drops were evaluated for their robustness to noise with (13) and (14).


(13) $$e_{\text{Σ}}=\frac{{\left\|\text{Δ}p_{\text{original}}(t)-\text{Δ}p_{\text{corrupted}}(t)\right\|}_{0,1}}{{\left\|\text{Δ}p_{\text{original}}(t)\right\|}_{0,1}}$$

(14) $$e_{\text{max}}=\frac{{\left\|\text{Δ}p_{\text{original}}\left(t_{n}\right)-\text{Δ}p_{\text{corrupted}}\left(t_{n}\right)\right\|}_{0,\infty }}{{\left\|\text{Δ}p_{\text{original}}\left(t_{n}\right)\right\|}_{0,\infty }}$$

### D. Flow Phantoms

PPE, STE, and *v*WERP were validated with an in vitro flow setup [20]. 4D flow MRI was acquired, and twelve pressure catheter measurements per TBAD tear variation served as ground truth.

#### 1) Flow Setup:

**Fig. 2.**
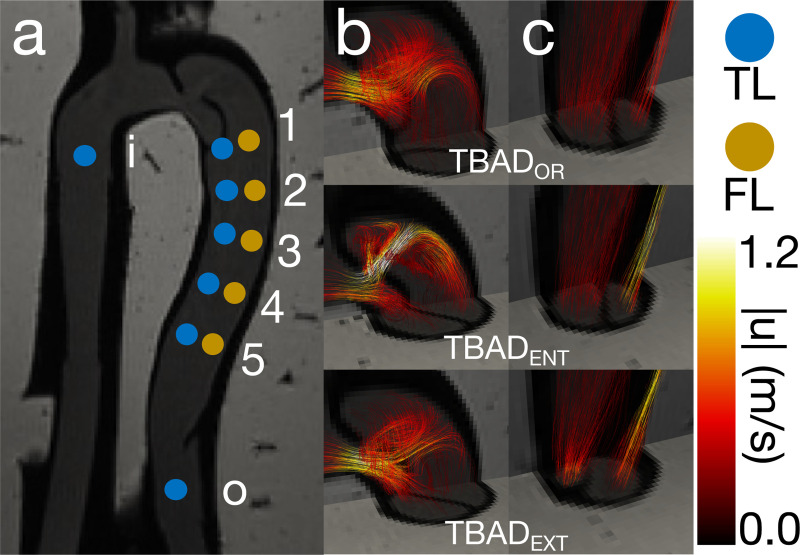
Overview of in vitro flow set up. (a) Catheter measurement points and cut planes across TBAD tear variations. (b) Entry tears across all TBAD tear variations. (c) Exit tears across all TBAD tear variations.

A full description of the flow phantom can be found in [20] (pump model, exact flow specifications, etc.). A brief overview is provided here. The flow phantom geometry was designed as an embedded compliant 3D-printed TBAD derived from a clinical CT exam. Physiologically accurate pulsatile flow and pressure boundary conditions were applied. 4D flow MRI (3T; voxel size = 1.5×1.5×1.5 mm; temporal resolution = 50 ms; VENC = 1.2–1.7 m/s) was acquired across three phantom variations: one baseline phantom (TBAD_OR_) and two additional variations with identical anatomy but with either a reduced size FL entry tear (TBAD_ENT_) or a reduced size FL exit tear (TBAD_EXT_) Fig. 2bc. Twelve catheter pressure measurements were acquired at consistent locations across each TBAD tear variation, for a total of 36 unique measurements (Fig. 2a).

#### 2) Validation Methodology:

Eleven time-resolved pressure drops derived from the twelve pressure catheter locations serve as the ground truth. To validate PPE and STE, the entire pressure field was calculated ($p_{\text{STE}}$, $p_{\text{PPE}}$), and individual pressure drops were found by spatially averaging the pressure values at each catheterization plane (i.e., the specified inlet and outlet planes) and calculating the difference at each time point to produce temporally-resolved, one-dimensional pressure drops. *v*WERP, being a plane-to-plane estimator, natively calculated these one-dimensional pressure drops, so no additional postprocessing was required. Linear regressions were performed to analyze the performance of each pressure estimator.

#### 3) Corruption Analysis:

As with the CFD model, the velocity fields were corrupted prior to pressure estimation in order to evaluate each method’s robustness to noise. Noise, however, is already present in the acquired 4D flow, so any additional perturbations are referred to as “corruptions”. Each velocity component ($u_{x}$, $u_{y}$, $u_{z}$) was corrupted with a zero-mean, truncated Gaussian [32] with standard deviation dependent on the level of additional corruption desired (ΔSNR) according to equation (15).


(15) $$\sigma_{v}=\frac{\sqrt{2}u_{\text{max}}}{\pi \cdot \text{ΔSNR}}$$

Each TBAD tear variation was evaluated over 50 realizations with ΔSNR=30 and ΔSNR=10, for a total of 300 corrupted realizations per pressure estimation method. Pressure drop deviations were calculated for each unique corrupted velocity field realization. This included measures of mean deviation (13) and max deviation (14). Error averages, minimums, and maximums were recorded across each of the 50 realization combos (e.g., TBAD_ENT_ at ΔSNR=10) and are reported in the supplement.

### E. Patient Cases

**Fig. 3.**
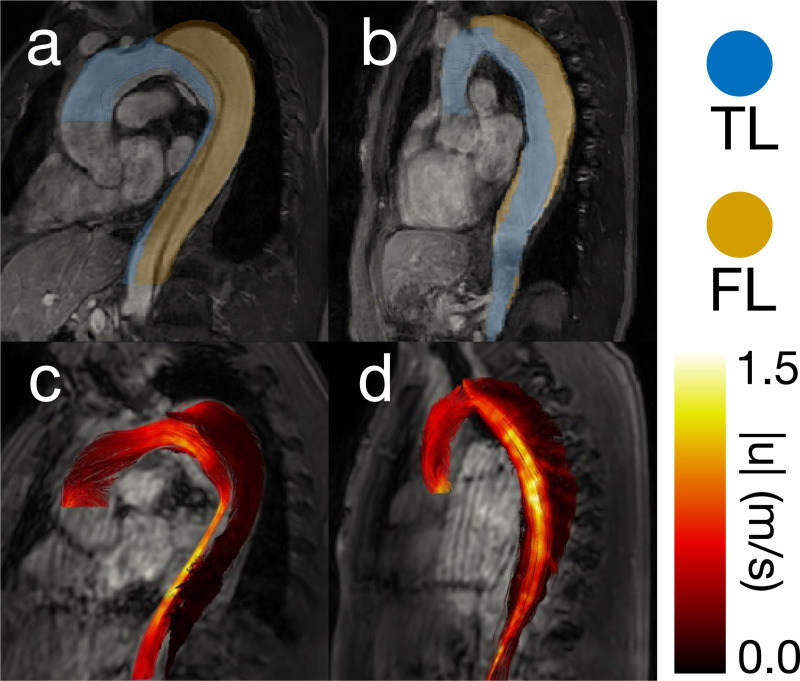
Two representative patient cases. (a) High-growth patient case MRA with 3D segmentation overlaid. The TL segmentation is blue, and the FL segmentation is gold. (b) Low-growth patient case MRA with 3D segmentation overlaid. (c) High-growth patient case 4D flow magnitude with velocity streamlines at peak systole overlaid. (d) Low-growth patient case 4D flow magnitude with velocity streamlines at peak systole overlaid.

The final analysis utilized eight patient cases (Fig. 3ab) to assess the clinical feasibility of pressure estimation methods and to qualitatively evaluate pressure trends against what is known in the literature, including our knowledge of FL growth rate [33]. 4D flow was acquired on 3T MRI scanners (MR750, GE Medical Systems, Milwaukee, WI, USA; Ingenia, Philips, Best, Netherlands) with the following scan parameters: voxel size = ∼1.5 mm × 1.5 mm × 2.5 mm, average temporal resolution = 47 ms, VENC = 2.0 m/s. Agreement between the pressure estimators was also evaluated. 4D flow was acquired from a previous IRB-approved study [34] (HUM00120679). The data was segmented by thresholding patient MRA data and subsequently interpolating the resulting segmentation onto the 4D flow image space (Fig. 3cd). Inlet and outlet planes were generated in a 4D flow MATLAB toolbox [35]. FL GR was measured clinically as the change in maximum aortic diameter. Each pressure estimator was run on all eight in vivo cases. The pressure fields calculated by the fully spatial pressure estimators were analyzed for their coherence. The 4D pressure fields were also converted to pressure drops (inlet–TL outlet and inlet–FL outlet) to allow for comparisons with *v*WERP.

## III. Results

### A. Evaluation of In Silico TBAD Model

#### 1) Spatiotemporal Analysis:

**Fig. 4.**
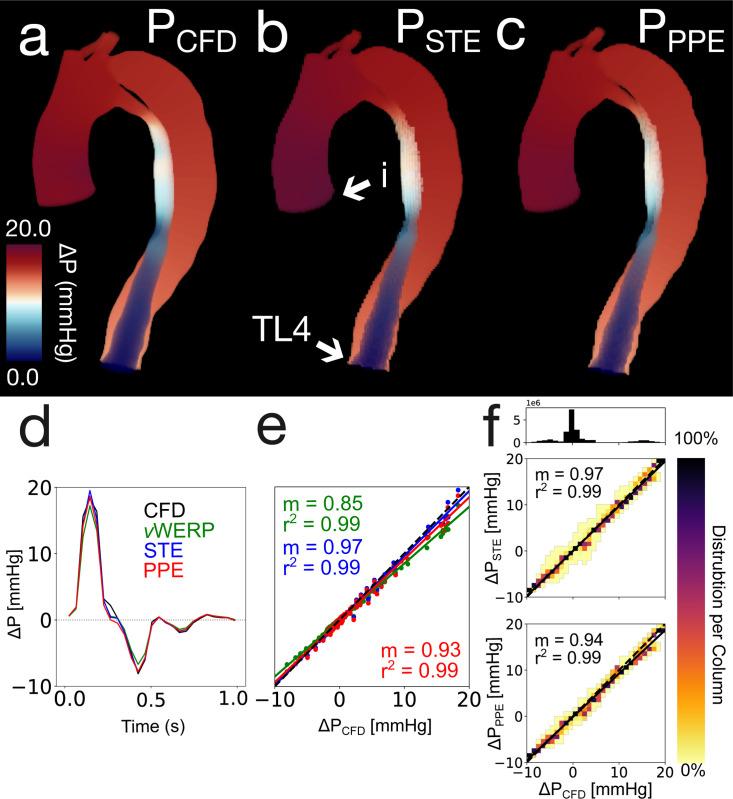
Overview of the in silico 1.5 mm × 40 ms results. (a) CFD ground truth pressure field at peak systole. (b) STE pressure field at peak systole. Aortic inlet (i) and true lumen outlet (TL4) are marked with arrow for subfigure d. (c) PPE pressure field at peak systole (d) Pressure drop from aortic inlet to TL outlet. (e) Linear regression of same pressure drop. (f) Linear regressions performed on STE and PPE pressure fields across all timesteps. The heatmaps indicate the distribution of pressure values in each 2D bin. The number of points in each 2D bin is normalized to the number of points in the entire column. The 1D histogram above the heatmap correlation plots illustrates the number of points present within each column.

Although the spatiotemporal analysis was performed on nine unique spatiotemporal resolutions, only the 1.5 mm × 40 ms results are visualized in Fig. 4. Additional correlation plots and pressure drop visualizations can be found in section 1 of the supplement. Fig. 4abc presents the CFD, PPE, and STE pressure fields, respectively, at peak systole. Relative error values with no added noise are recorded in Table I. Fig. 4f shows the correlation between the pressure fields calculated by STE, PPE, versus the CFD ground truth. The estimated pressure fields were highly consistent — both $p_{\text{STE}}$ and $p_{\text{PPE}}$ were very strongly correlated with $p_{\text{CFD}}$. PPE consistently mildly underestimated the ground truth pressure compared to STE.

Fig. 4e (top) shows the pressure drop from the aortic inlet to the TL outlet as calculated by *v*WERP, STE, and PPE. Table II summarizes the pressure drop linear regression results for the spatiotemporal analysis. All correlation coefficients were near unity for the 1.5 mm and 2.0 mm images but degraded dramatically for the 3.0 mm images. PPE correlation coefficients decreased the most dramatically for the 3.0 mm cases. Additional press drop linear regression plots can be found in subsection 1.5 of the supplement.

#### 2) Sensitivity Analysis Results:

**Fig. 5.**
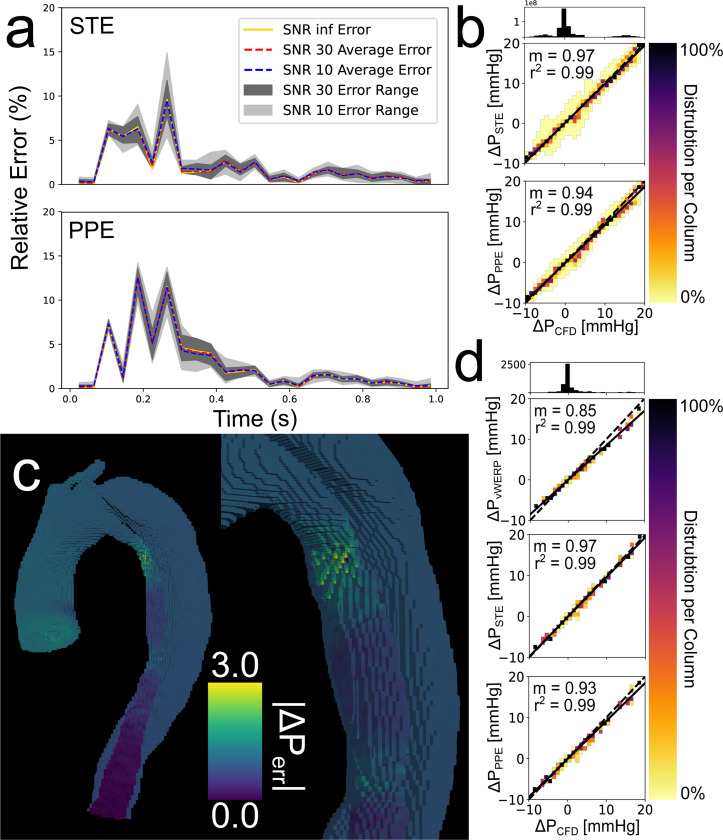
Overview of in silico 1.5 mm × 40 ms sensitivity analysis. (a) Relative error over cardiac cycle. (b) Correlation heatmaps for the fully spatial analysis over all 25 noise realizations. (c) Pressure error map (d) Correlation heatmaps for the plane-to-plane pressure drop analysis across all 25 noise realizations. The heatmaps indicate the distribution of pressure values in each 2D bin. The number of points in each 2D bin is normalized to the number of points in the entire column. The 1D histogram above the heatmap correlation plots illustrates the number of points present within each column.

The sensitivity analysis for the 1.5 mm × 40 ms resolution is summarized in Fig. 5. The relative error over the cardiac cycle (11) is shown in Fig. 5a for STE and PPE. Although both methods had similar maximum errors, STE was slightly more accurate on average. Correlation heatmaps for the fully spatial analysis and the plane-to-plane pressure drop analysis at SNR = 10 are shown in Fig. 5b and d, respectively. A pressure error map for an SNR = 10 1.5 mm × 40 ms case is shown in Fig. 5c. Errors were lowest in the distal true lumen. In general, errors grew when moving proximally from the true lumen outlet. The exception to this trend was in the extremely narrow region of the proximal true lumen, which is shown in more detail in Fig. 5c (right). The range of total relative errors (12) is shown in Table III. Averaged total relative errors can be found in subsection 1.3 of the supplement. Relative errors for STE were lower than for PPE; however, STE generally had larger ranges of errors, indicating that it was more sensitive to noise.

In general, all methods were resilient to noise; only the 3.0 mm pressure traces in supplement subsection 1.6 had a noticeable spread of pressure values due to corrupted velocity fields. Furthermore, the calculated deviation values indicated that the amount of deviation from the baseline (no noise) cases increased substantially for the 3.0 mm cases. Across all resolutions and methods, the deviation values for the 1.5 mm and 2.0 mm cases remained below 4% in almost all cases and frequently below 2%. Of the 3.0 mm cases, the *v*WERP estimations showed the highest deviations from the baseline case, followed by STE, and then PPE a distant third place.

### B. Flow Phantom Analysis

#### 1) Validation of Pressure Estimators:

**Fig. 6.**
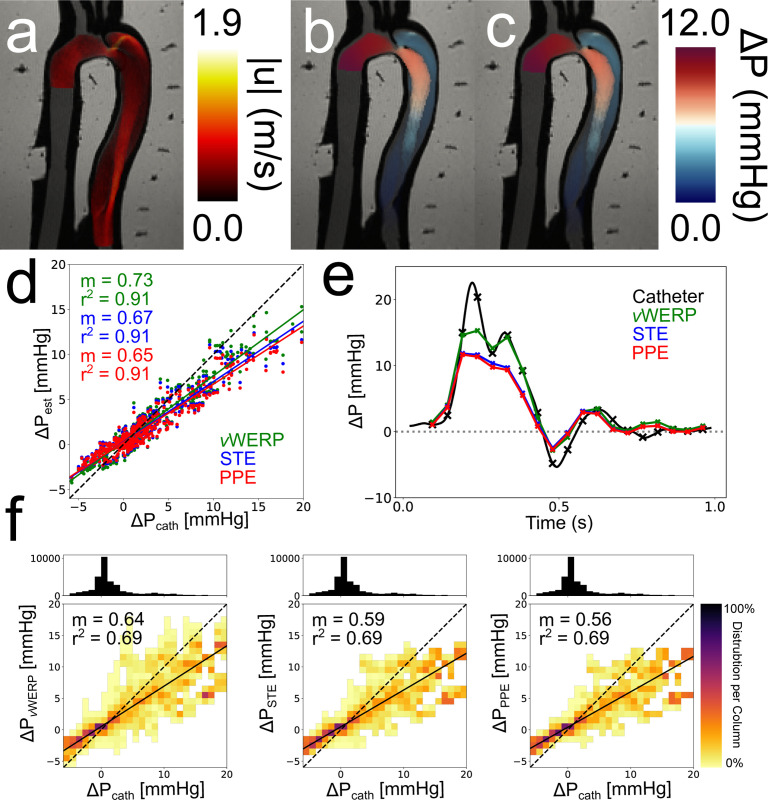
Overview of in vitro flow phantom analysis. (a) TBAD_ENT_ velocity field at peak systole. (b) TBAD_ENT_ STE pressure field at peak systole. (c) TBAD_ENT_ PPE pressure field at peak systole. (d) Linear regression across all models and planes with no corruption. (e) Example pressure trace over the cardiac cycle. The pressure drop was calculated from the model inlet to the model outlet (i to o in Fig. 2). The “x” markers indicate where 4D flow timeframes were present. (f) Correlation heatmaps for each method. Linear regressions were run over all 50 ΔSNR=10 corruption realizations per TBAD tear variation for a total of 150 unique datasets per correlation heatmap. The heatmaps indicate the distribution of pressure values in each 2D bin. The number of points in each 2D bin is normalized to the number of points in the entire column. The 1D histogram above the heatmap correlation plots illustrates the number of points present within each column.

A broad overview of the flow phantom analysis results is given in Fig. 6, with TBAD_ENT_ chosen as the representative TBAD tear variation. More extensive results, including those for TBAD_OR_ and TBAD_ENT_, can be found in the supplement. Qualitatively, the pressure fields estimated by STE and PPE demonstrated the features one would expect from observing the TBAD_ENT_ velocity field (Fig. 6a). For instance, the high pressure impingement zone immediately following the entry tear in Fig. 6bc closely resembles the impingement zone in the corresponding FSI model of TBAD_ENT_, shown in Fig. 4c of Zimmermann et al. [20]. Both the STE (Fig. 6b) and PPE (Fig. 6c) pressure fields exhibited a large pressure drop across the FL entry tear, an increase in pressure where the FL entry tear jet impinged on the wall of the FL, and higher pressure drops from the aortic inlet (i) to each TL measurement point (TL 1–5) compared to each FL measurement point (FL 1–5). The TBAD_OR_ and TBAD_EXT_ pressure fields were similarly coherent with our expectations. Fig. 6df includes data from all TBAD tear variations. Across all TBAD tear variations, *v*WERP, STE, and PPE were strongly correlated $\left(r^{2}=0.91\right)$ with catheter measurements (Fig. 6d), with a tendency to underestimate peak pressures (Fig. 6e). This trend holds across all pressure traces, which can be explored in further detail in subsection 2.3 of the supplement.

#### 2) Corruption Analysis:

Fig. 6f illustrates the effect of the ΔSNR=10 corruption across all 50 noise realizations for each pressure estimation method. Corruption of the velocity fields mildly degraded the average accuracy, while the correlations were moderately weaker $\left(r^{2}=0.69\right)$. The pressure drop mean deviation (13) and max deviation (14) results for TBAD_ENT_ are summarized in Table IV. For the FL and TL rows, deviations were averaged across all numbered pressure drops for that lumen (e.g., aortic inlet to TL1, aortic inlet to TL2, etc.). Additional tables for TBAD_OR_ and TBAD_EXT_ can be found in the supplement in subsection 2.2, and a more in-depth analysis for each pressure trace and the range of estimations can be found in the supplement subsection 2.3. As expected, the average deviation increased with noise. Average deviations were also higher in the true lumen across all models. PPE was significantly more resilient to corruption compared to *v*WERP and STE — the average PPE deviations were roughly half of the other method’s deviations.

### C. Patient Cases

**Fig. 7.**
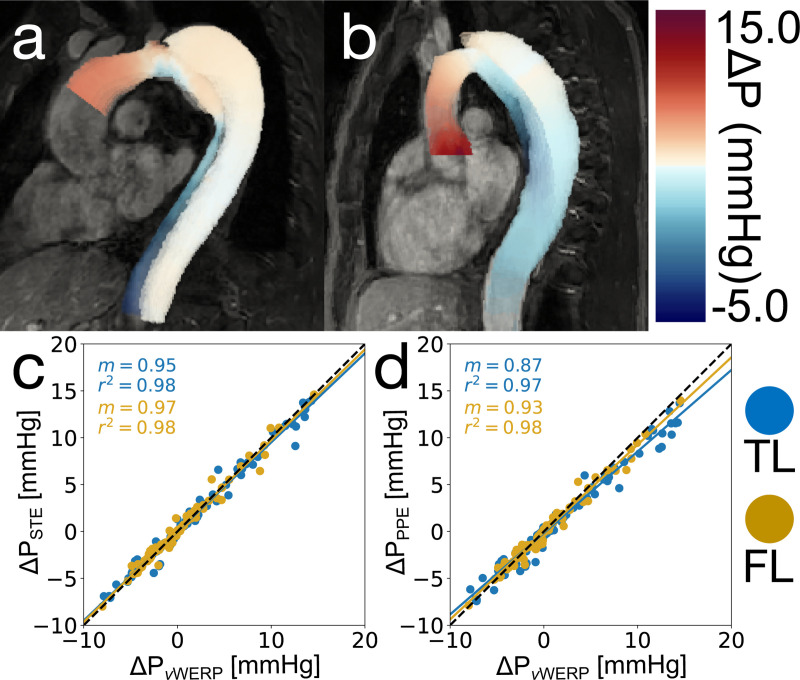
In vivo analysis results. (a) STE pressure field of a high-growth patient case at peak systole. (b) STE pressure field of a low-growth patient case at peak systole. (c) TL and FL correlation plot of STE vs. *v*WERP across all patient cases. (d) TL and FL correlation plot of PPE vs. *v*WERP across all patient cases.

For all eight patient cases, PPE and STE produced pressure fields that were coherent with our expectations — relative pressure was highest in the aortic arch and steadily decreased when moving distally. Pressure drops were visibly higher in the TL, where most of the net flow was present [36]. Pressure drops in the FL were generally lower than in the TL, although the magnitude of this trend was dependent on the calculated growth rate of each patient [33]. As expected, lower FL growth rate patients had a higher pressure drop across the FL, and higher FL growth rate patients had a lower pressure drop across the FL (Fig. 7ab). The PPE, STE, and *v*WERP pressure drops were analyzed for agreement in Fig. 7cd, and additional comparisons can be found in section 3 of the supplement. Correlations between all pressure estimation methods were extremely strong $\left(r^{2}=0.97-0.98\right)$. Pressure drops calculated by *v*WERP tended to be the highest, followed by STE (m=0.95−0.97 when compared to *v*WERP), and finally, PPE (m=0.87−0.93 compared to *v*WERP). This ordering of the results matched the outcome of the in vitro phantom analysis, where *v*WERP also produced the highest pressure drops.

Standard practice was to run the patient cases with nearest-neighbor upsampling by a factor of 2. At this level, pressure estimations were completed in a matter of minutes as timed by MATLAB’s timeit function. Timing results for a representative patient case were: 527.4 s for STE, 151.4 s for PPE, 192.7 s for *v*WERP when calculating the FL pressure drop, and 41.3 s for *v*WERP when calculating the TL pressure drop.

## IV. Discussion

The pressure estimators *v*WERP, STE, and PPE were evaluated across in silico, in vitro, and in vivo TBAD datasets. The in silico analysis was performed on a CFD model with varying spatiotemporal resolution and synthetic noise levels. Notably, the in silico analysis allowed for comparisons between estimated pressure fields and the ground truth CFD solution. This allowed for a novel analysis of the spatial distributions of errors. The in vitro analysis was performed on a compliant TBAD flow phantom with three unique entry and exit tear variants. Twelve pressure catheter measurements were made in each flow phantom tear variation, combining the benefits of acquired 4D flow and significantly more ground truth pressure measurements than would be allowed clinically. The in vivo analysis was performed on eight patient cases. While no ground truth was present, this analysis allowed for a qualitative view of pressure fields and a chance to observe the relative pressure estimators in a clinical context.

### A. Spatial Error Distribution

**Fig. 8.**
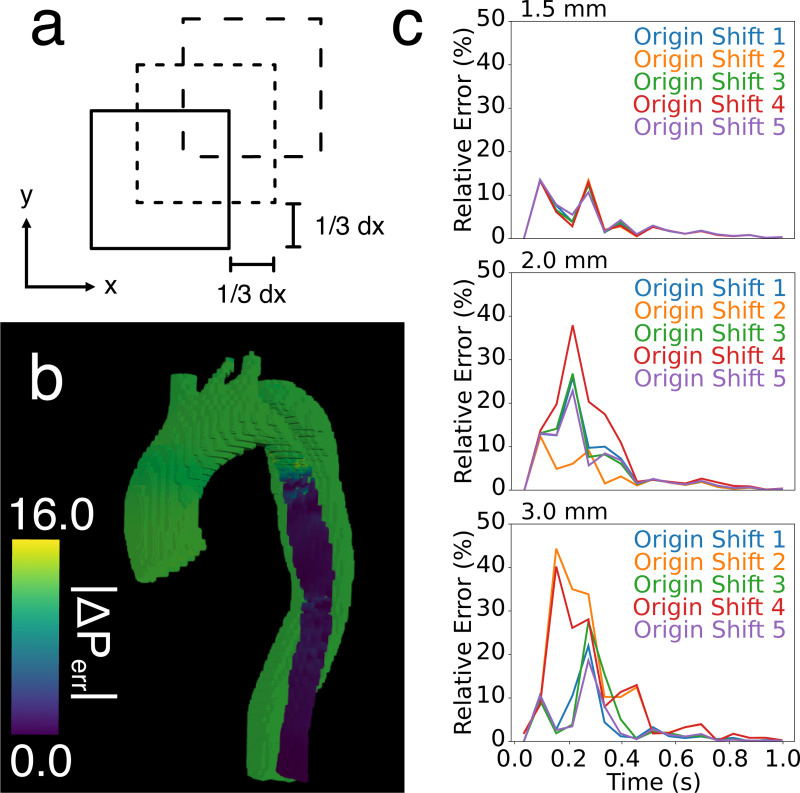
Overview of in silico origin shift analysis for STE. (a) Overview of origin shift method for one voxel in 2D. Each square indicates a different shift in the origin by a subvoxel amount, forcing a different interpolation. (b) Example of the large errors in the FL and proximal TL after and unfavorable shit. (c) Relative errors over the cardiac cycle for five different origin shifts. Temporal resolution was fixed to 60 ms for each variation in spatial resolution.

An important novelty of the fully spatial in silico analysis was the ability to precisely analyze the spatial variation of errors in the estimated pressure fields. In general, errors were highest on the vessel wall boundaries and in areas with high velocity gradients. These two factors combined to become particularly impactful in the 3.0 mm in silico images — an extremely narrow region in the proximal TL had high velocities, leading to sharp velocity gradients and extensive partial voluming. Thus, absolute errors of over 15 mmHg occurred and relative errors of over 100% occurred, as seen in Fig. 8b and Table I. To further corroborate that partial voluming/interpolation was causing high errors, an additional analysis was performed by translating the CFD model by 1/3 of a given voxel size. Each translation forced different interpolations of the boundary, changing TL pressure drops and relative error. One can imagine a 6 mm diameter region that is either more accurately represented by two 3 mm voxels or poorly represented after a 1 mm translation, which causes the same data to be represented over three voxels with partial voluming.

In the 1.5 mm and 2.0 mm cases, the TL pressure drop was resilient to shifting the model; however, the 3.0 mm case gave drastically different pressure drops depending on the shift, ranging from 10.9 mmHg to 18.2 mmHg as the maximum pressure drop. Because the relative pressures were calculated in reference to the TL outlet, errors in the proximal TL shifted the rest of the estimated pressure field by a constant for both STE and PPE. This behavior leads to high errors when compared to the ground truth CFD data (and when compared to the TL pressure drop ground truth), but *not* when compared to the FL pressure drop ground truth. Because the rest of the model proximal to the narrow TL was disrupted by the same constant, the four FL pressure drops we probed remained accurate.

In the patient cases, small segments of 2–8 voxels with erroneous velocity measurements led to small regions (2–8 voxels as well) with massive pressure errors. High (but localized) erroneous velocity gradients led to corresponding localized pressure errors. In response to these findings, care was taken to pre-process patient data to remove 4D flow voxels with magnitudes/orientations significantly different from their neighbors. If STE and PPE are used clinically, care must be taken to appropriately filter out erroneous velocity measurements to ensure errors are not magnified in the corresponding relative pressure fields. Errors generally increased when moving proximally from the TL outlet, which is expected given that the relative pressure fields were shifted in reference to the TL outlet pressure.

### B. Effect of Resolution

Increased spatial resolution generally increased the accuracy of the estimated pressure fields. Accuracy dipped when temporal resolution decreased mainly because the pressure estimators did not have the time frames available to capture the highly transient pressure drop at peak systole. Errors were primarily caused by underestimating pressure extrema (both systolic peaks and diastolic valleys) and a temporal shift in the latter half of the cardiac cycle. This behavior was more drastic in the 4D flow MRI acquisitions compared to the synthetic 4D flow images. The synthetic 4D flow images were constructed with instantaneous velocity measurements, which meant there was no temporal averaging. Temporal averaging, of course, occurred in the 4D flow scans. In the in silico analysis, the estimators may have missed peak pressures, but they accurately estimated the pressures based on the time frames they had access to. Meanwhile, the 4D flow scans both undersampled the peak systolic pressure drop and temporally averaged the time frames that were sampled, leading to more pronounced underestimation.

### C. Effect of Noise

In agreement with Bertoglio et al. [14], the average errors across all noisy realizations were roughly equivalent to the cases with no added noise, experimentally confirming that *v*WERP, STE, and PPE are unbiased estimators. Whereas higher resolution was almost always better in the SNR=inf cases, this trend did not precisely hold in the noisy cases. Low spatial resolution combined with high temporal resolution resulted in a higher spread in estimated pressures, although the average pressures across noise realizations were still close to the SNR=inf cases. Deviations from the baseline pressure drop estimation were also routinely higher in the TL across both in silico and in vitro analyses.

### D. Pros and Cons of Pressure Estimators

Each pressure estimator recovered flow phantom ground truth pressures with a minimum correlation coefficient of 0.9 and a tendency to mildly underestimate peak pressure drops, albeit with varying severity. More clear differences in accuracy were present, however, in the sensitivity and spatiotemporal analyses. Based on our analysis, *v*WERP should be the preferred estimator when calculating one-dimensional pressure drops. It had the highest accuracy in the 4D flow MRI acquisitions and was significantly faster than STE. Although *v*WERP performed worse than PPE and STE in the in silico cases, *v*WERP’s improved performance in the in vitro cases suggests that there are aspects of MRI simulation that we are missing in our synthetic MRI generation methodology. When a fully 3D pressure field is desired, the ideal estimator depends on the SNR and resolution of the 4D flow acquisition. STE should be the standard choice (especially at lower resolutions). However, if a low SNR is expected, PPE may be better than STE. Similar to [15], STE outperformed PPE in terms of accuracy; however, PPE appeared to have a slight edge in its resistance to noise. PPE’s additional resistance to noise may be due to the slightly different implementation of PPE, which was implemented as an FVM rather than an FDM. It should be noted that Nolte et al. [15] used a different implementation of PPE and STE, utilizing backward differences for temporal derivatives (rather than midpoint) and an FEM. Although PPE was faster than STE (roughly 2.5 minutes vs. 8.7 minutes of CPU time), both methods calculated pressure fields in a reasonable amount of time.

### E. Limitations and Future Work

A synthetic MRI generation protocol accepted in the past was utilized [31] but could be improved [37], [38]. Future work would involve integrating more advanced synthetic MRI modeling to determine results in hypothetical clinical scenarios more accurately. The CFD model design and synthetic image generation procedure were both reasonably straightforward. This decision was made because the projection to a regular grid required that an extremely large number of elements (roughly 2.6 million) be present to preserve higher-order derivatives when projecting to the finer grid resolutions. Although this model was sufficiently representative, future work could involve an FSI model with compliant walls. Finally, the in vivo analysis did not include patient catheter data, although such invasive data is not typically acquired (particularly in the FL) in patients with aortic dissection.

## V. Conclusion

In this work, we analyzed *v*WERP, STE, and PPE over in silico, in vitro, and in vivo datasets. Of the fully spatial estimators, STE was shown to be more accurate overall, whereas PPE was shown to be more resilient to noise. *v*WERP did not perform as well as either STE or PPE in the in silico analysis. In the in vitro analysis, STE continued to outperform PPE in accuracy but was mildly less resilient to corruption of the velocity field. *v*WERP moderately outperformed both STE and PPE in accuracy. PPE remained the most resilient to noise. Finally, the pressure estimators were evaluated qualitatively and for agreement over patient cases. PPE and STE agreed strongly, and *v*WERP tended to estimate the largest pressure drop, mimicking the trend of the in vitro analysis. All pressure estimators were remarkably resilient to noise and successfully estimated the ground truth pressure, albeit with tendencies to underestimate. The time to compute the pressure estimations was on the order of minutes, demonstrating the possibility of being integrated into a clinical workflow.

## Supplementary Material

Supplement 1

## Figures and Tables

**TABLE I:** In Silico Total Relative Error $\left(\varepsilon_{\text{REL}}\right)$ for Fully Spatial Pressure Fields

	STE			PPE		

	20 ms	40 ms	60 ms	20 ms	40 ms	60 ms
		
1.5 mm	3.33%	8.76%	16.89%	8.30%	11.98%	16.69%
2.0 mm	19.57%	18.34%	24.83%	6.79%	8.51%	13.69%
3.0 mm	78.98%	80.43%	85.87%	101.76%	103.10%	110.15%

**TABLE II:** In Silico Pressure Drop Slopes and Correlations

		*v*WERP			STE			PPE		

		20 ms	40 ms	60 ms	20 ms	40 ms	60 ms	20 ms	40 ms	60 ms
				
1.5 mm	*m*	0.86	0.85	0.92	0.99	0.92	1.04	0.94	0.93	0.99
	*r* ^2^	1.00	0.99	0.98	1.00	0.99	0.98	1.00	0.99	0.97
2.0 mm	*m*	0.89	0.88	0.96	1.09	1.08	1.17	1.01	0.99	1.06
	*r* ^2^	0.98	0.98	0.99	0.98	0.98	0.99	0.99	0.99	0.99
3.0 mm	*m*	0.36	0.35	0.36	0.54	0.52	0.54	0.42	0.41	0.40
	*r* ^2^	0.36	0.34	0.30	0.39	0.37	0.33	0.21	0.19	0.16

**TABLE III:** In Silico Total Relative Error $\left(\varepsilon_{\text{REL}}\right)$ Range for Sensitivity Analysis

		STE			PPE		

SNR	dx/dt	20 ms	40 ms	60 ms	20 ms	40 ms	60 ms
			
30	1.5 mm	2.08%	1.74%	1.48%	1.61%	2.08%	2.34%
	2.0 mm	3.43%	4.48%	5.20%	2.72%	2.52%	3.14%
	3.0 mm	8.04%	12.50%	16.18%	11.75%	12.97%	16.90%
10	1.5 mm	1.28%	3.24%	3.37%	2.76%	4.00%	4.20%
	2.0 mm	7.06%	5.14%	5.69%	2.59%	2.78%	6.15%
	3.0 mm	23.97%	28.18%	30.06%	15.42%	16.99%	28.98%

**TABLE IV:** In Vitro Average Deviations Across TBAD_ENT_

	*v*WERP				STE				PPE			

	SNR = 30		SNR = 10		SNR = 30		SNR = 10		SNR = 30		SNR = 10	
			
	$e_{\text{Σ}}$	$e_{\text{max}}$	$e_{\text{Σ}}$	$e_{\text{max}}$	$e_{\text{Σ}}$	$e_{\text{max}}$	$e_{\text{Σ}}$	$e_{\text{max}}$	$e_{\text{Σ}}$	$e_{\text{max}}$	$e_{\text{Σ}}$	$e_{\text{max}}$
						
FL	1.52%	1.45%	5.41%	4.73%	1.20%	1.22%	4.42%	3.83%	0.57%	0.60%	1.81%	1.85%
TL	2.95%	2.27%	12.69%	8.90%	2.76%	2.15%	10.80%	7.72%	1.38%	1.11%	4.49%	3.50%
Outlet	2.38%	2.50%	8.55%	8.25%	1.22%	1.16%	4.73%	4.15%	0.58%	0.57%	1.88%	1.75%

## References

[1] Pibarot P. and Dumesnil J. G., “Improving Assessment of Aortic Stenosis,” Journal of the American College of Cardiology, vol. 60, no. 3, pp. 169–180, Jul. 2012. 22789881 10.1016/j.jacc.2011.11.078

[2] Kim Y. Y., Andrade L., and Cook S. C., “Aortic Coarctation,” Cardiology Clinics, vol. 38, no. 3, pp. 337–351, Aug. 2020. 32622489 10.1016/j.ccl.2020.04.003

[3] Neuss M., Schau T., Isotani A., Pilz M., Schöpp M., and Butter C., “Elevated Mitral Valve Pressure Gradient After MitraClip Implantation Deteriorates Long-Term Outcome in Patients With Severe Mitral Regurgitation and Severe Heart Failure,” JACC: Cardiovascular Interventions, vol. 10, no. 9, pp. 931–939, May 2017. 28473116 10.1016/j.jcin.2016.12.280

[4] Bäumler K., Rolf-Pissarczyk M., Schussnig R., Fries T.-P., Mistelbauer G., Pfaller M. R., Marsden A. L., Fleischmann D., and Holzapfel G. A., “Assessment of aortic dissection remodeling with patient-specific fluid-structure interaction models,” IEEE Trans. Biomed. Eng., pp. 1–17, 2024. 10.1109/TBME.2024.3480362 PMC12994412 39401111

[5] Anjum I., Khan M. A., Aadil M., Faraz A., Farooqui M., and Hashmi A., “Transradial vs. Transfemoral Approach in Cardiac Catheterization: A Literature Review,” Cureus, Jun. 2017. 10.7759/cureus.1309 PMC5493462 28690943

[6] Harris P. and Kuppurao L., “Quantitative Doppler echocardiography,” BJA Education, vol. 16, no. 2, pp. 46–52, Feb. 2016.

[7] Markl M., Frydrychowicz A., Kozerke S., Hope M., and Wieben O., “4D flow MRI,” Magnetic Resonance Imaging, vol. 36, no. 5, pp. 1015–1036, Nov. 2012. 10.1002/jmri.23632 23090914

[8] Van Ooij P., Powell A. L., Potters W. V., Carr J. C., Markl M., and Barker A. J., “Reproducibility and interobserver variability of systolic blood flow velocity and 3D wall shear stress derived from 4D flow MRI in the healthy aorta: Reproducibility of 3D WSS in 4D Flow MRI,” J. Magn. Reson. Imaging, vol. 43, no. 1, pp. 236–248, Jan. 2016. 26140480 10.1002/jmri.24959 PMC4807608

[9] Binter C., Gotschy A., Sundermann S. H., Frank M., Tanner F. C., Lüscher T. F., Manka R., and Kozerke S., “Turbulent Kinetic Energy Assessed by Multipoint 4-Dimensional Flow Magnetic Resonance Imaging Provides Additional Information Relative to Echocardiography for the Determination of Aortic Stenosis Severity,” Circ: Cardiovascular Imaging, vol. 10, no. 6, p. e005486, Jun. 2017. 28611119 10.1161/CIRCIMAGING.116.005486

[10] Azarine A., Garçon P., Stansal A., Canepa N., Angelopoulos G., Silvera S., Sidi D., Marteau V., and Zins M., “Four-dimensional Flow MRI: Principles and Cardiovascular Applications,” RadioGraphics, vol. 39, no. 3, pp. 632–648, May 2019. 30901284 10.1148/rg.2019180091

[11] Nienaber C. A., Clough R. E., Sakalihasan N., Suzuki T., Gibbs R., Mussa F., Jenkins M. P., Thompson M. M., Evangelista A., Yeh J. S. M., Cheshire N., Rosendahl U., and Pepper J., “Aortic dissection,” Nat Rev Dis Primers, vol. 2, no. 1, p. 16053, Jul. 2016. 27440162 10.1038/nrdp.2016.53

[12] François C. J., Markl M., Schiebler M. L., Niespodzany E., Landgraf B. R., Schlensak C., and Frydrychowicz A., “Four-dimensional, flow-sensitive magnetic resonance imaging of blood flow patterns in thoracic aortic dissections,” The Journal of Thoracic and Cardiovascular Surgery, vol. 145, no. 5, pp. 1359–1366, May 2013. 22841438 10.1016/j.jtcvs.2012.07.019 PMC3514600

[13] Callaghan F. M., Kozor R., Sherrah A. G., Vallely M., Celermajer D., Figtree G. A., and Grieve S. M., “Use of multi-velocity encoding 4D flow MRI to improve quantification of flow patterns in the aorta: Multi-VENC 4D Flow,” J. Magn. Reson. Imaging, vol. 43, no. 2, pp. 352–363, Feb. 2016. 26130421 10.1002/jmri.24991

[14] Bertoglio C., Nuñez R., Galarce F., Nordsletten D., and Osses A., “Relative pressure estimation from velocity measurements in blood flows: State-of-the-art and new approaches: Relative pressure estimation,” Int J Numer Meth Biomed Engng, vol. 34, no. 2, p. e2925, Feb. 2018. 10.1002/cnm.2925 28884520

[15] Nolte D., Urbina J., Sotelo J., Sok L., Montalba C., Valverde I., Osses A., Uribe S., and Bertoglio C., “Validation of 4D Flow based relative pressure maps in aortic flows,” Medical Image Analysis, vol. 74, p. 102195, Dec. 2021. 34419837 10.1016/j.media.2021.102195

[16] Nath R., Kazemi A., Callahan S., Stoddard M. F., and Amini A. A., “4Dflow-VP-Net: A deep convolutional neural network for noninvasive estimation of relative pressures in stenotic flows from 4D flow MRI,” Magnetic Resonance in Med, vol. 90, no. 5, pp. 2175–2189, Nov. 2023. 10.1002/mrm.29791 PMC10615364 37496183

[17] Casas B., Lantz J., Dyverfeldt P., and Ebbers T., “4D Flow MRI-based pressure loss estimation in stenotic flows: Evaluation using numerical simulations,” Magnetic Resonance in Med, vol. 75, no. 4, pp. 1808–1821, Apr. 2016. 10.1002/mrm.25772 26016805

[18] Saitta S., Pirola S., Piatti F., Votta E., Lucherini F., Pluchinotta F., Carminati M., Lombardi M., Geppert C., Cuomo F., Figueroa C. A., Xu X. Y., and Redaelli A., “Evaluation of 4D flow MRI-based noninvasive pressure assessment in aortic coarctations,” Journal of Biomechanics, vol. 94, pp. 13–21, Sep. 2019. 31326119 10.1016/j.jbiomech.2019.07.004

[19] Marlevi D., Ruijsink B., Balmus M., Dillon-Murphy D., Fovargue D., Pushparajah K., Bertoglio C., Colarieti-Tosti M., Larsson M., Lamata P., Figueroa C. A., Razavi R., and Nordsletten D. A., “Estimation of Cardiovascular Relative Pressure Using Virtual Work-Energy,” Sci Rep, vol. 9, no. 1, p. 1375, Feb. 2019. 30718699 10.1038/s41598-018-37714-0 PMC6362021

[20] Zimmermann J., Bäumler K., Loecher M., Cork T. E., Marsden A. L., Ennis D. B., and Fleischmann D., “Hemodynamic effects of entry and exit tear size in aortic dissection evaluated with in vitro magnetic resonance imaging and fluid–structure interaction simulation,” Sci Rep, vol. 13, no. 1, p. 22557, Dec. 2023. 38110526 10.1038/s41598-023-49942-0 PMC10728172

[21] Kundu P. K., Cohen I. M., Dowling D. R., and Capecelatro J., Fluid Mechanics, 7th ed., ser. MATLAB Examples. London San Diego, CA Cambridge, MA: Academic Press, 2025.

[22] Ebbers T., Wigström L., Bolger A. F., Wranne B., and Karlsson M., “Noninvasive Measurement of Time-Varying Three-Dimensional Relative Pressure Fields Within the Human Heart,” Journal of Biomechanical Engineering, vol. 124, no. 3, pp. 288–293, Jun. 2002. 12071263 10.1115/1.1468866

[23] Švihlová H, Hron J, Málek J, Rajagopal K, and Rajagopal K, “Determination of pressure data from velocity data with a view toward its application in cardiovascular mechanics. Part 1. Theoretical considerations,” International Journal of Engineering Science, vol. 105, pp. 108–127, Aug. 2016.

[24] Švihlová H., Hron J., Málek J., Rajagopal K., and Rajagopal K., “Determination of pressure data from velocity data with a view towards its application in cardiovascular mechanics. Part 2. A study of aortic valve stenosis,” International Journal of Engineering Science, vol. 114, pp. 1–15, May 2017.

[25] Nguyen T.-Q., Traberg M. S., Bjerring Olesen J., Moshavegh R., Moller-Sorensen P. H., Lonn L., Jensen J. A., Bachmann Nielsen M., and Hansen K. L., “Pressure Difference Estimation in Carotid Bulbs using Vector Flow Imaging - A Phantom Study,” in 2019 IEEE International Ultrasonics Symposium (IUS). Glasgow, United Kingdom: IEEE, Oct. 2019, pp. 872–875.

[26] Lechner V., “Image-based Mapping of Regional Relative Pressures Using the Pressure Poisson Equation: Evaluations on Dynamically Varying Domains in a Cardiovascular Setting,” Master’s thesis, KTH Royal Institute of Technology, 2023.

[27] Jolesz F. A., Ed., Intraoperative Imaging and Image-Guided Therapy. New York, NY: Springer New York, 2014.

[28] “SimModeler,” Simmetrix Inc.

[29] Lee J., Cookson A., Roy I., Kerfoot E., Asner L., Vigueras G., Sochi T., Deparis S., Michler C., Smith N. P., and Nordsletten D. A., “Multiphysics Computational Modeling in CHeart,” SIAM J. Sci. Comput., vol. 38, no. 3, pp. C150–C178, Jan. 2016.

[30] Ahrens J., Geveci B., and Law C., “ParaView: An End-User Tool for Large-Data Visualization,” in Visualization Handbook. Elsevier, 2005, pp. 717–731.

[31] Ferdian E., Suinesiaputra A., Dubowitz D. J., Zhao D., Wang A., Cowan B., and Young A. A., “4DFlowNet: Super-Resolution 4D Flow MRI Using Deep Learning and Computational Fluid Dynamics,” Front. Phys., vol. 8, p. 138, May 2020.

[32] Robert C. P., “Simulation of truncated normal variables,” Stat Comput, vol. 5, no. 2, pp. 121–125, Jun. 1995.

[33] Marlevi D., Sotelo J. A., Grogan-Kaylor R., Ahmed Y., Uribe S., Patel H. J., Edelman E. R., Nordsletten D. A., and Burris N. S., “False lumen pressure estimation in type B aortic dissection using 4D flow cardiovascular magnetic resonance: Comparisons with aortic growth,” J Cardiovasc Magn Reson, vol. 23, no. 1, p. 51, Dec. 2021. 33980249 10.1186/s12968-021-00741-4 PMC8117268

[34] Burris N. S., Nordsletten D. A., Sotelo J. A., Grogan-Kaylor R., Houben I. B., Figueroa C. A., Uribe S., and Patel H. J., “False lumen ejection fraction predicts growth in type B aortic dissection: Preliminary results,” European Journal of Cardio-Thoracic Surgery, vol. 57, no. 5, pp. 896–903, May 2020. 31821480 10.1093/ejcts/ezz343 PMC8453376

[35] Sotelo J., “JulioSoteloParraguez/4D-Flow-Matlab-Toolbox,” Feb. 2024.

[36] Xu H., Xiong J., Han X., Mei Y., Shi Y., Wang D., Zhang M., and Chen D., “Computed tomography-based hemodynamic index for aortic dissection,” The Journal of Thoracic and Cardiovascular Surgery, vol. 162, no. 2, pp. e165–e176, Aug. 2021. 32217023 10.1016/j.jtcvs.2020.02.034

[37] Dirix P., Buoso S., Peper E. S., and Kozerke S., “Synthesis of patient-specific multipoint 4D flow MRI data of turbulent aortic flow downstream of stenotic valves,” Sci Rep, vol. 12, no. 1, p. 16004, Sep. 2022. 36163357 10.1038/s41598-022-20121-x PMC9513106

[38] Weine J., McGrath C., Dirix P., Buoso S., and Kozerke S., “CMRSIM –A python package for cardiovascular MR simulations incorporating complex motion and flow,” Magnetic Resonance in Med, p. mrm.30010, Jan. 2024. 10.1002/mrm.30010 38234037

