# Supplementary material for: Comprehensive Analysis of Relative Pressure Estimation Methods Utilizing 4D Flow MRI
Source: ArXiv. 2025 Mar 4:arXiv:2503.02847v1. Preprint. [Version 1] (PMC11908371)
Supplement: Supplementary file 1 [file NIHPP2503.02847v1-supplement-1.pdf]

Supplement to Comprehensive Analysis of Relative Pressure Estimation Methods Utilizing 4D Flow MRI

Brandon Hardy

March 5, 2025

arXiv:2503.02847v1 [physics.flu-dyn] 4 Mar 2025

Contents

1 CFD Model **2**
- 1.1 Fully Spatial Correlation Plots . . . 2
- 1.2 Fully Spatial Relative Error Plots . . . 5
- 1.3 Relative Error Tables . . . 7
- 1.4 Pressure Drop Baseline Scatter Plots . . . 9
- 1.5 Pressure Drop Correlation Plots . . . 10
- 1.6 Pressure Drop Figures . . . 15
  - 1.6.1 vWERP . . . 15
  - 1.6.2 STE . . . 19
  - 1.6.3 PPE . . . 23
- 1.7 Deviation Tables . . . 27

2 In Vitro Model **30**
- 2.1 Pressure Drop Correlation Plots . . . 30
- 2.2 Deviation Tables . . . 31
- 2.3 Pressure Drop Figures . . . 34
  - 2.3.1 vWERP . . . 34
  - 2.3.2 STE . . . 36
  - 2.3.3 PPE . . . 38

3 Patient Cases **40**
- 3.1 Individual Patients . . . 40
- 3.2 Pressure Drop Correlation Plots . . . 42

1 CFD Model

1.1 Fully Spatial Correlation Plots

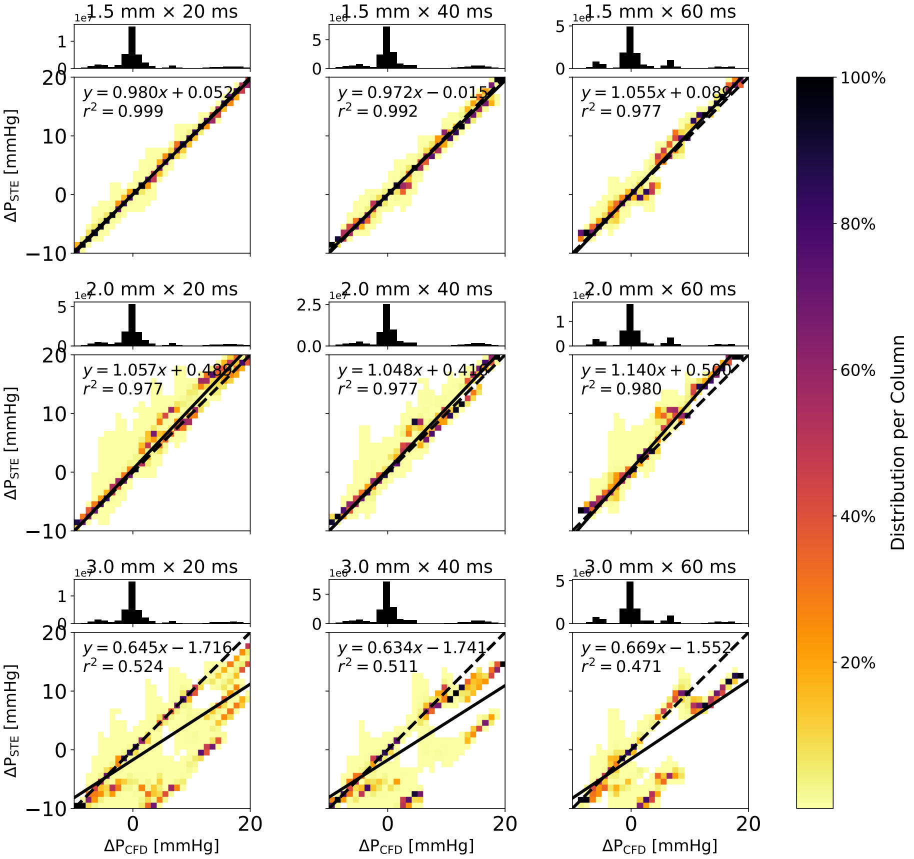

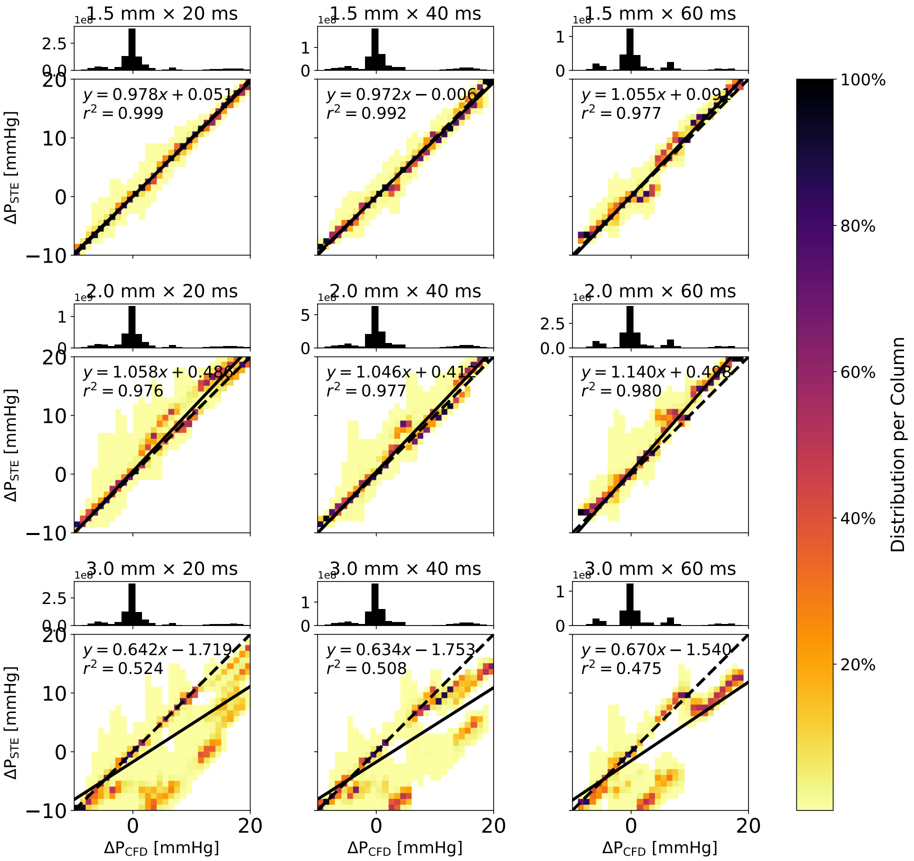
1.5 mm × 20 ms
y = 0.978x + 0.051
r² = 0.999
1.5 mm × 40 ms
y = 0.972x − 0.006
r² = 0.992
1.5 mm × 60 ms
y = 1.055x + 0.09
r² = 0.977
2.0 mm × 20 ms
y = 1.058x + 0.486
r² = 0.976
2.0 mm × 40 ms
y = 1.046x + 0.41
r² = 0.977
2.0 mm × 60 ms
y = 1.140x + 0.498
r² = 0.980
3.0 mm × 20 ms
y = 0.642x − 1.719
r² = 0.524
3.0 mm × 40 ms
y = 0.634x − 1.753
r² = 0.508
3.0 mm × 60 ms
y = 0.670x − 1.540
r² = 0.475
ΔP_STE [mmHg]
ΔP_CFD [mmHg]
Distribution per Column
100%
80%
60%
40%
20%

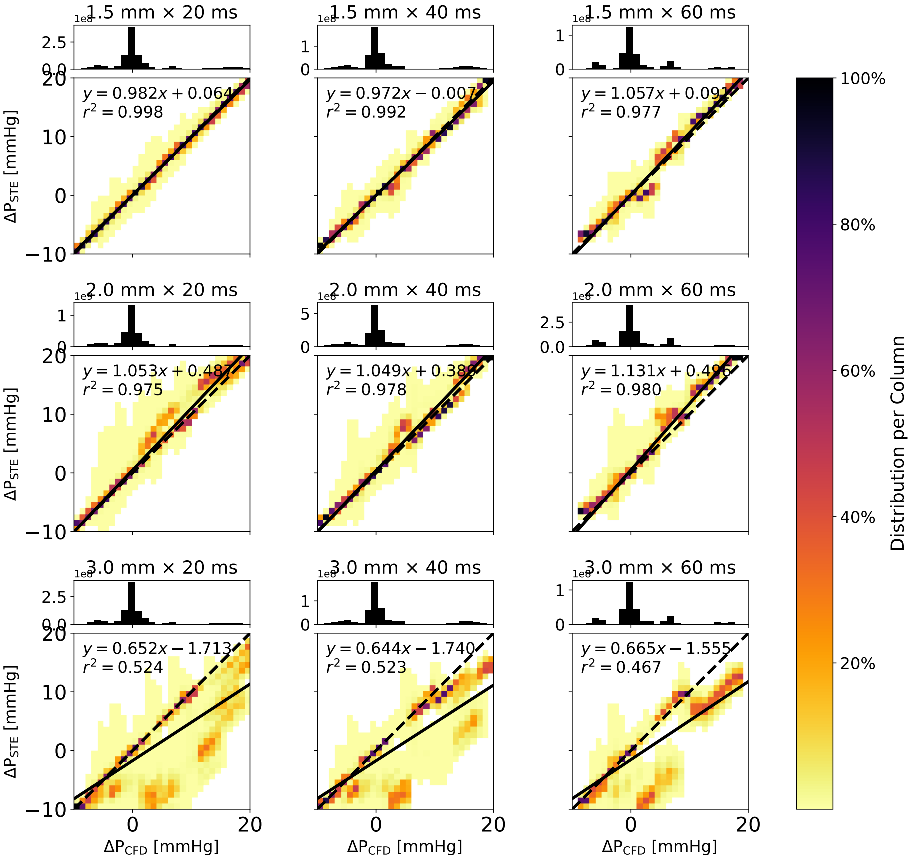
1.5 mm × 20 ms
y = 0.982x + 0.064
r² = 0.998
1.5 mm × 40 ms
y = 0.972x − 0.007
r² = 0.992
1.5 mm × 60 ms
y = 1.057x + 0.09
r² = 0.977
2.0 mm × 20 ms
y = 1.053x + 0.487
r² = 0.975
2.0 mm × 40 ms
y = 1.049x + 0.38
r² = 0.978
2.0 mm × 60 ms
y = 1.131x + 0.496
r² = 0.980
3.0 mm × 20 ms
y = 0.652x − 1.713
r² = 0.524
3.0 mm × 40 ms
y = 0.644x − 1.740
r² = 0.523
3.0 mm × 60 ms
y = 0.665x − 1.555
r² = 0.467
ΔP_STE [mmHg]
ΔP_CFD [mmHg]
Distribution per Column
100%
80%
60%
40%
20%

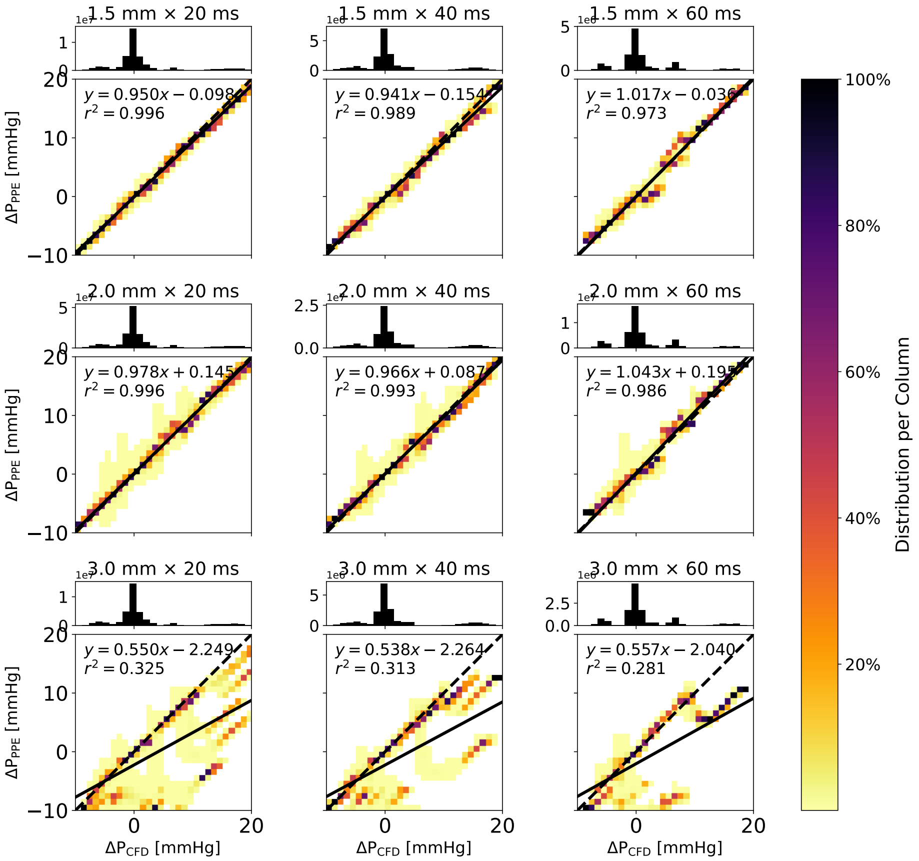
1.5 mm × 20 ms
y = 0.950x − 0.098
r² = 0.996
1.5 mm × 40 ms
y = 0.941x − 0.154
r² = 0.989
1.5 mm × 60 ms
y = 1.017x − 0.036
r² = 0.973
2.0 mm × 20 ms
y = 0.978x + 0.145
r² = 0.996
2.0 mm × 40 ms
y = 0.966x + 0.087
r² = 0.993
2.0 mm × 60 ms
y = 1.043x + 0.195
r² = 0.986
3.0 mm × 20 ms
y = 0.550x − 2.249
r² = 0.325
3.0 mm × 40 ms
y = 0.538x − 2.264
r² = 0.313
3.0 mm × 60 ms
y = 0.557x − 2.040
r² = 0.281
ΔP_PPE [mmHg]
ΔP_CFD [mmHg]
Distribution per Column
100%
80%
60%
40%
20%

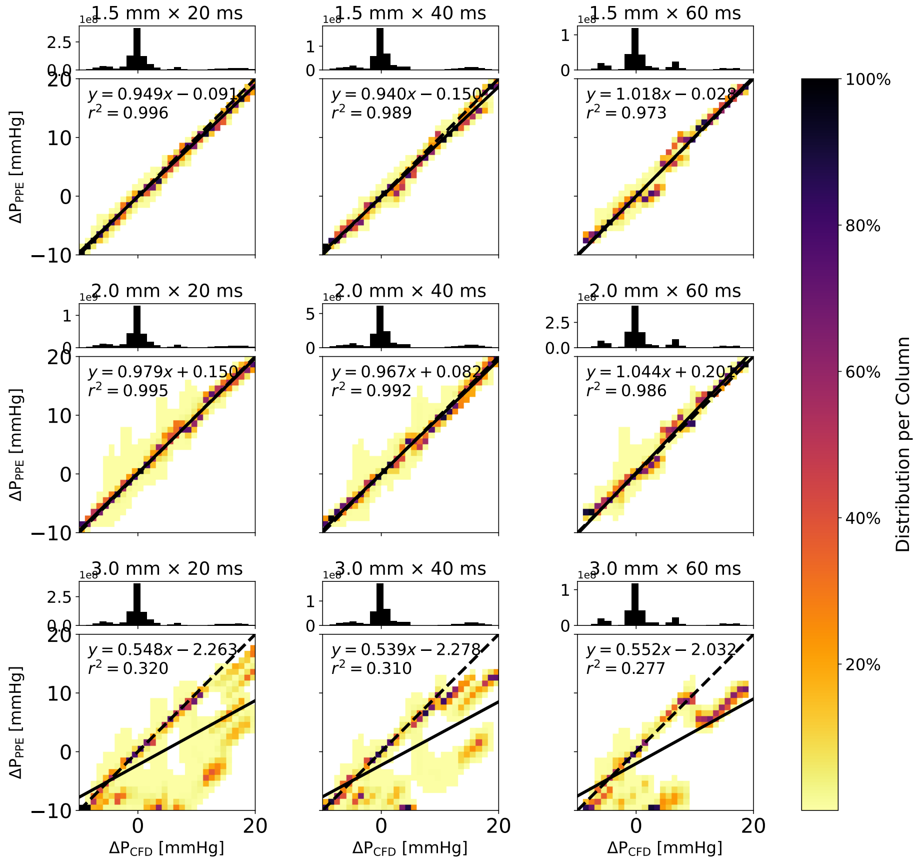
1.5 mm × 20 ms
y = 0.949x − 0.091
r² = 0.996
1.5 mm × 40 ms
y = 0.940x − 0.150
r² = 0.989
1.5 mm × 60 ms
y = 1.018x − 0.028
r² = 0.973
2.0 mm × 20 ms
y = 0.979x + 0.150
r² = 0.995
2.0 mm × 40 ms
y = 0.967x + 0.082
r² = 0.992
2.0 mm × 60 ms
y = 1.044x + 0.201
r² = 0.986
3.0 mm × 20 ms
y = 0.548x − 2.263
r² = 0.320
3.0 mm × 40 ms
y = 0.539x − 2.278
r² = 0.310
3.0 mm × 60 ms
y = 0.552x − 2.032
r² = 0.277
ΔP_PPE [mmHg]
ΔP_CFD [mmHg]
Distribution per Column
100%
80%
60%
40%
20%

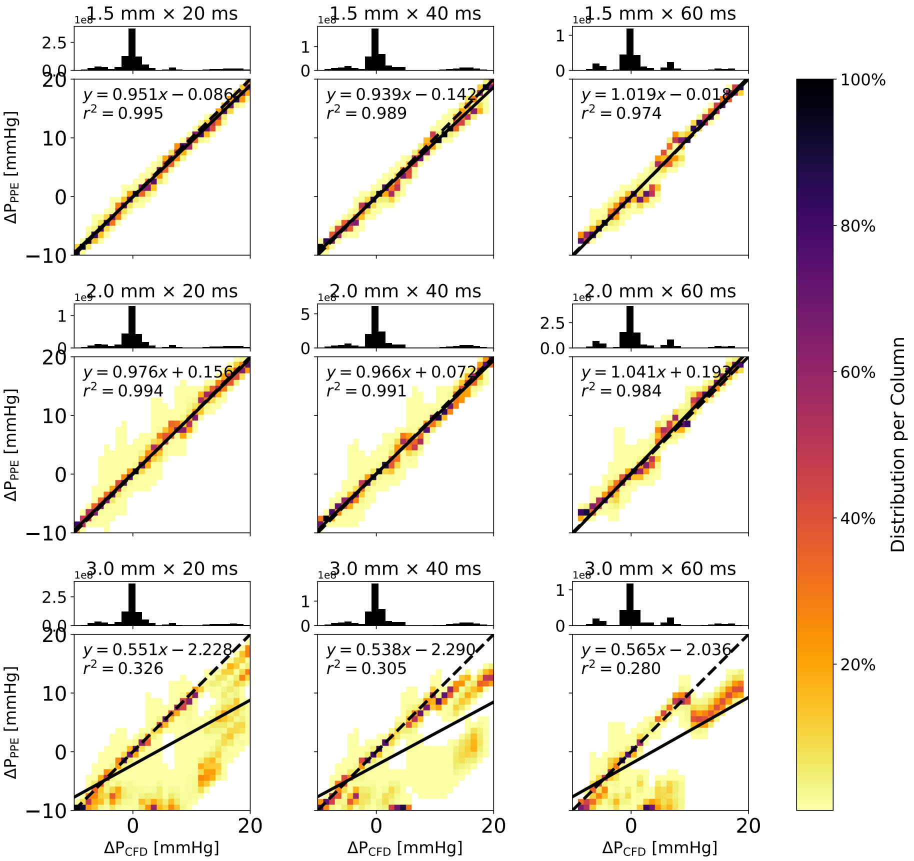

1.2 Fully Spatial Relative Error Plots

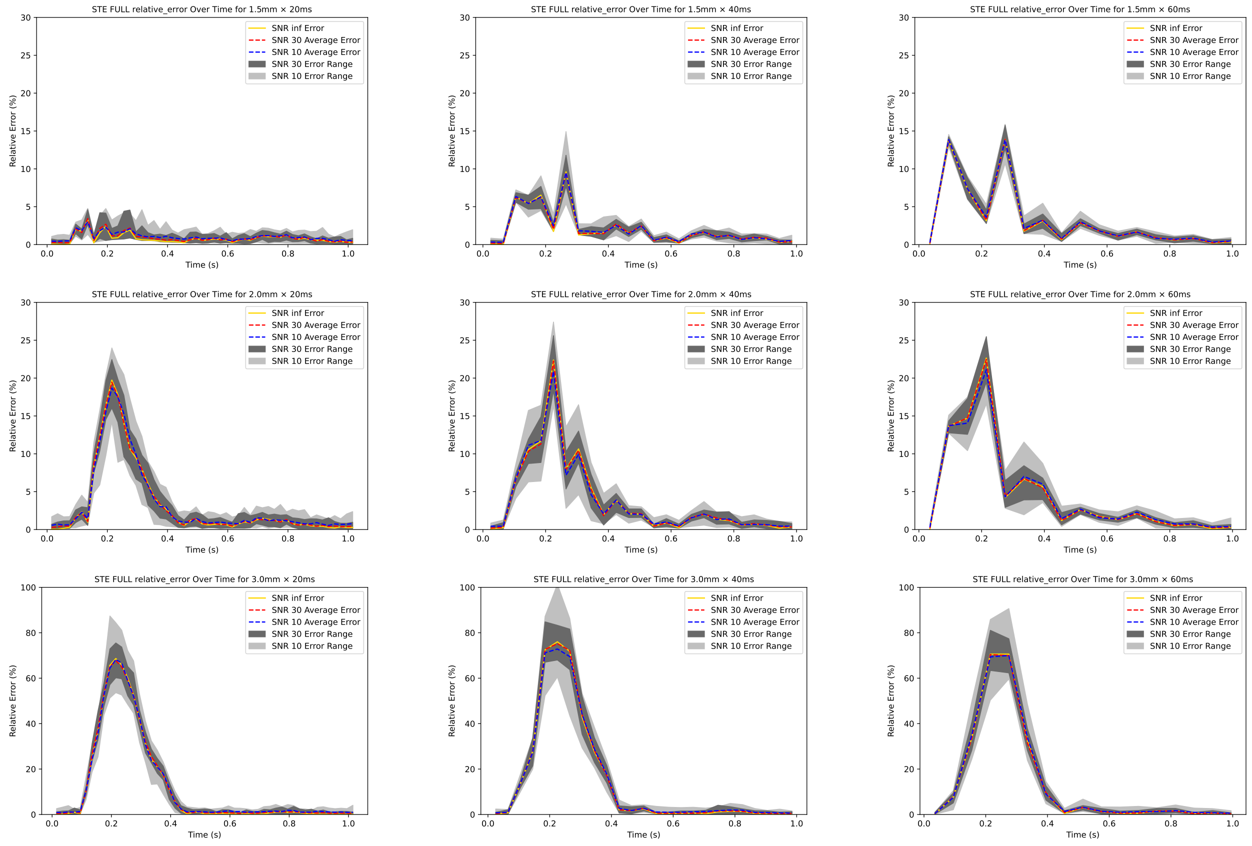

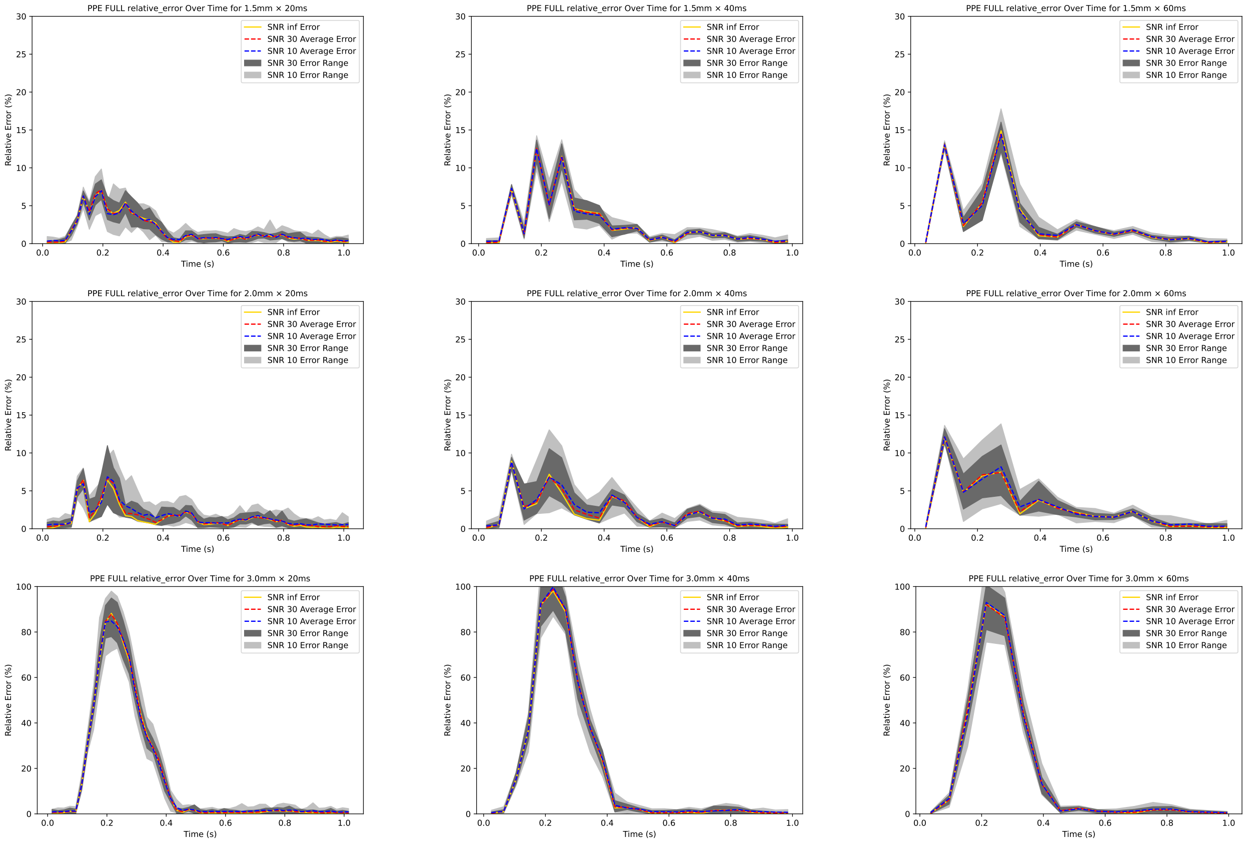
PPE FULL relative_error Over Time for 1.5mm × 20ms
PPE FULL relative_error Over Time for 1.5mm × 40ms
PPE FULL relative_error Over Time for 1.5mm × 60ms
PPE FULL relative_error Over Time for 2.0mm × 20ms
PPE FULL relative_error Over Time for 2.0mm × 40ms
PPE FULL relative_error Over Time for 2.0mm × 60ms
PPE FULL relative_error Over Time for 3.0mm × 20ms
PPE FULL relative_error Over Time for 3.0mm × 40ms
PPE FULL relative_error Over Time for 3.0mm × 60ms
SNR inf Error
SNR 30 Average Error
SNR 10 Average Error
SNR 30 Error Range
SNR 10 Error Range
Relative Error (%)
Time (s)

1.3 Relative Error Tables

Average Total Relative Error for Fully Spatial Analysis

SNR inf STE

	20ms	40ms	60ms
1.5mm	3.33%	8.76%	16.89%
2.0mm	19.57%	18.34%	24.83%
3.0mm	78.98%	80.43%	85.87%

SNR inf PPE

	20ms	40ms	60ms
1.5mm	8.30%	11.98%	16.69%
2.0mm	6.79%	8.51%	13.69%
3.0mm	101.76%	103.10%	110.15%

SNR 30 STE

	20ms	40ms	60ms
1.5mm	3.88%	8.76%	16.97%
2.0mm	19.62%	18.19%	24.80%
3.0mm	78.99%	80.72%	85.36%

SNR 30 PPE

	20ms	40ms	60ms
1.5mm	8.41%	12.04%	16.55%
2.0mm	7.48%	8.86%	13.98%
3.0mm	102.44%	103.68%	110.18%

SNR 10 STE

	20ms	40ms	60ms
1.5mm	4.28%	8.98%	17.13%
2.0mm	19.77%	17.96%	24.14%
3.0mm	78.99%	79.23%	86.02%

SNR 10 PPE

	20ms	40ms	60ms
1.5mm	8.45%	12.00%	16.47%
2.0mm	8.04%	9.40%	14.37%
3.0mm	101.39%	104.40%	110.51%

SNR

Method

Figure 3: Table showing averaged total relative error across all noise realizations. Note that since $n_{\text{realize}} = 1$ for SNR=inf, the average is equivalent to the total relative error for this single case.

Total Relative Error Range for Fully Spatial Analysis

SNR

SNR 30 STE

	20ms	40ms	60ms
1.5mm	2.08%	1.74%	1.48%
2.0mm	3.43%	4.84%	5.20%
3.0mm	8.04%	12.50%	16.18%

SNR 30 PPE

	20ms	40ms	60ms
1.5mm	1.61%	2.08%	2.34%
2.0mm	2.72%	2.52%	3.14%
3.0mm	11.75%	12.97%	16.90%

SNR 10 STE

	20ms	40ms	60ms
1.5mm	1.28%	3.24%	3.37%
2.0mm	7.06%	5.14%	5.69%
3.0mm	23.97%	28.18%	30.06%

SNR 10 PPE

	20ms	40ms	60ms
1.5mm	2.76%	4.00%	4.20%
2.0mm	2.59%	2.78%	6.15%
3.0mm	15.42%	16.99%	28.98%

Method

1.4 Pressure Drop Baseline Scatter Plots

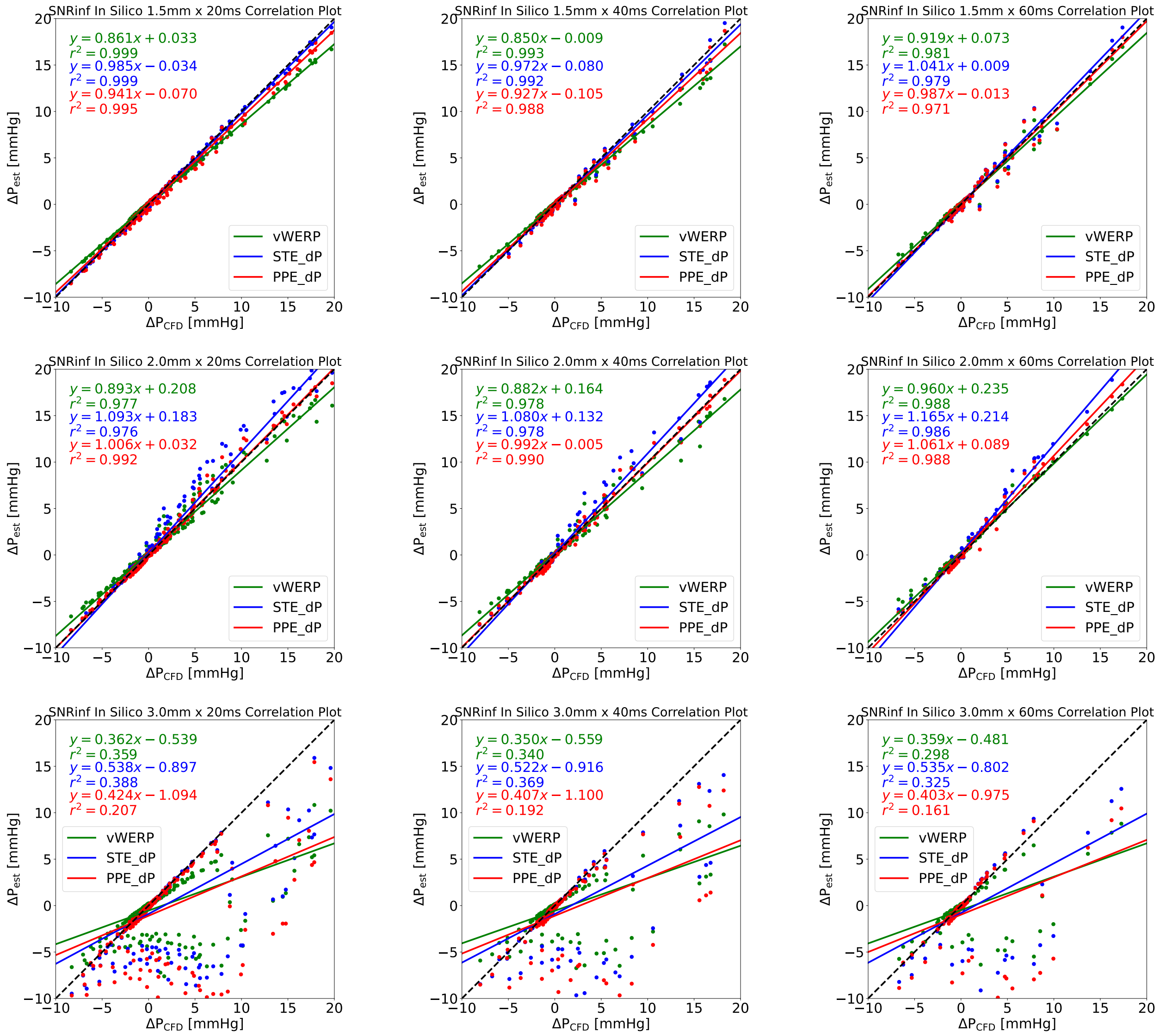

1.5 Pressure Drop Correlation Plots

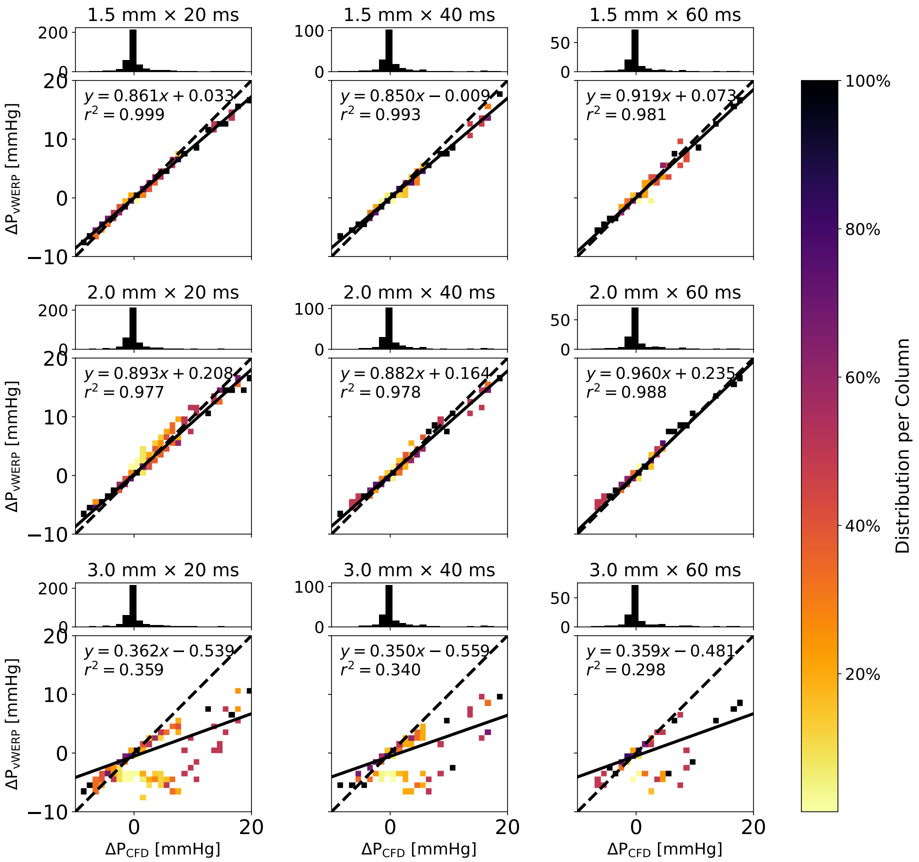

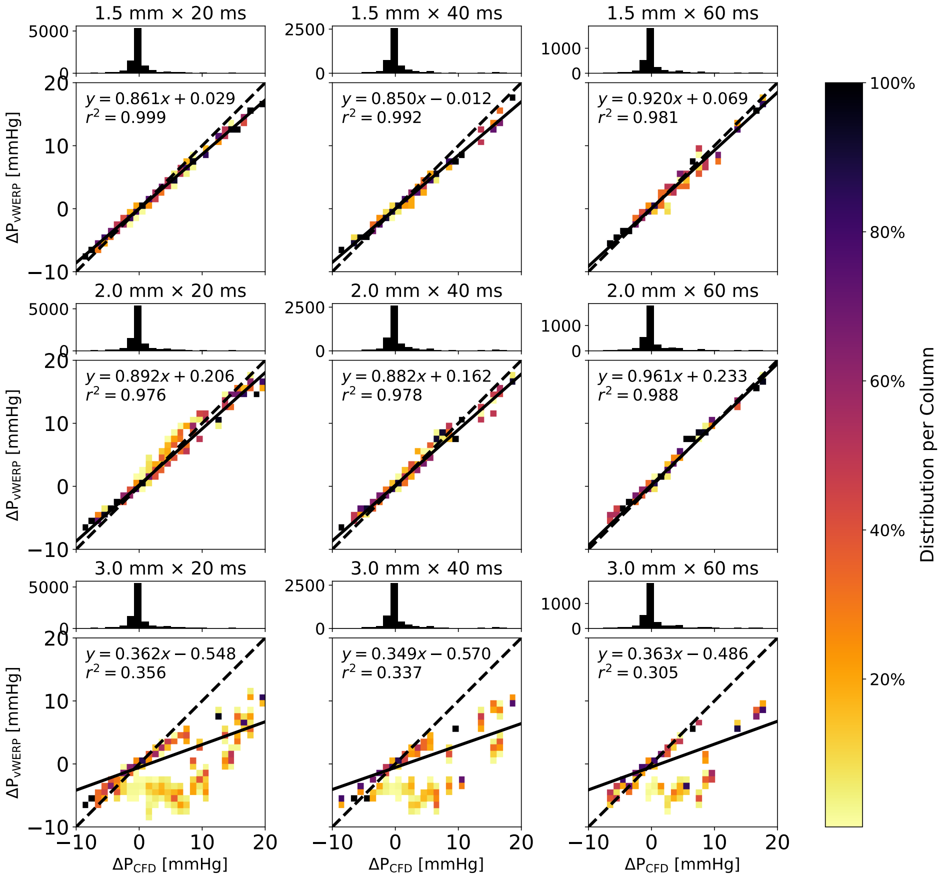

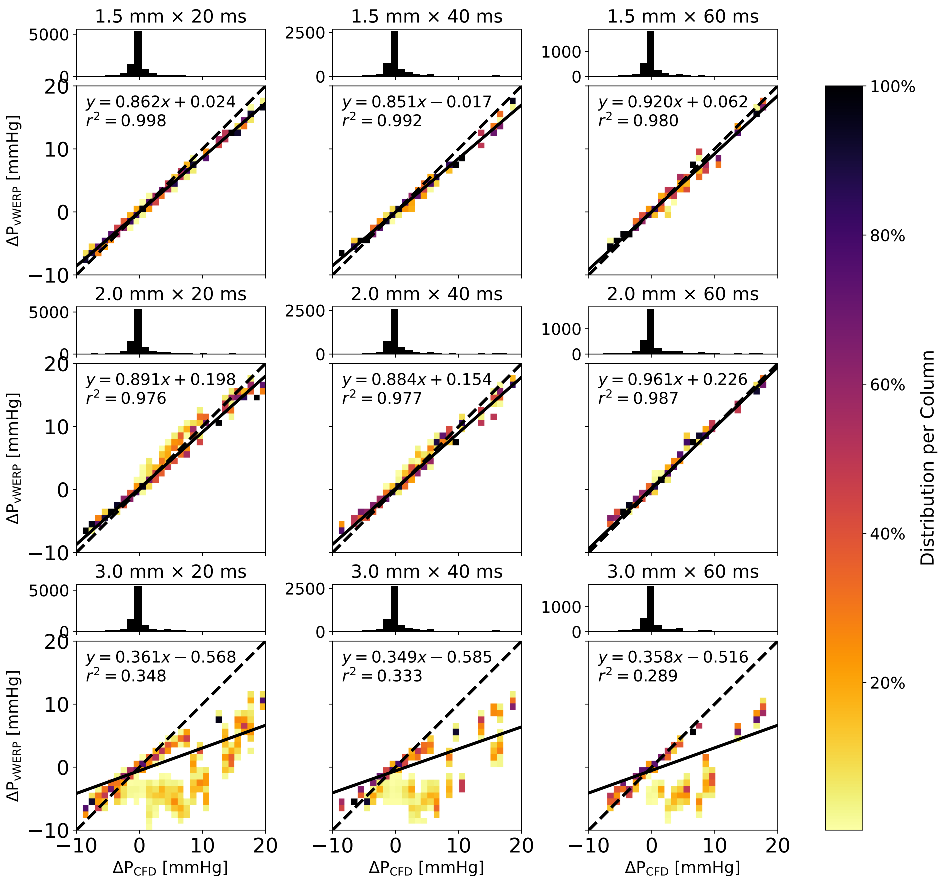
1.5 mm × 20 ms
1.5 mm × 40 ms
1.5 mm × 60 ms
2.0 mm × 20 ms
2.0 mm × 40 ms
2.0 mm × 60 ms
3.0 mm × 20 ms
3.0 mm × 40 ms
3.0 mm × 60 ms
$y = 0.862x + 0.024$
$r^2 = 0.998$
$y = 0.851x - 0.017$
$r^2 = 0.992$
$y = 0.920x + 0.062$
$r^2 = 0.980$
$y = 0.891x + 0.198$
$r^2 = 0.976$
$y = 0.884x + 0.154$
$r^2 = 0.977$
$y = 0.961x + 0.226$
$r^2 = 0.987$
$y = 0.361x - 0.568$
$r^2 = 0.348$
$y = 0.349x - 0.585$
$r^2 = 0.333$
$y = 0.358x - 0.516$
$r^2 = 0.289$
ΔP_{vWERP} [mmHg]
ΔP_{CFD} [mmHg]
Distribution per Column
100%
80%
60%
40%
20%

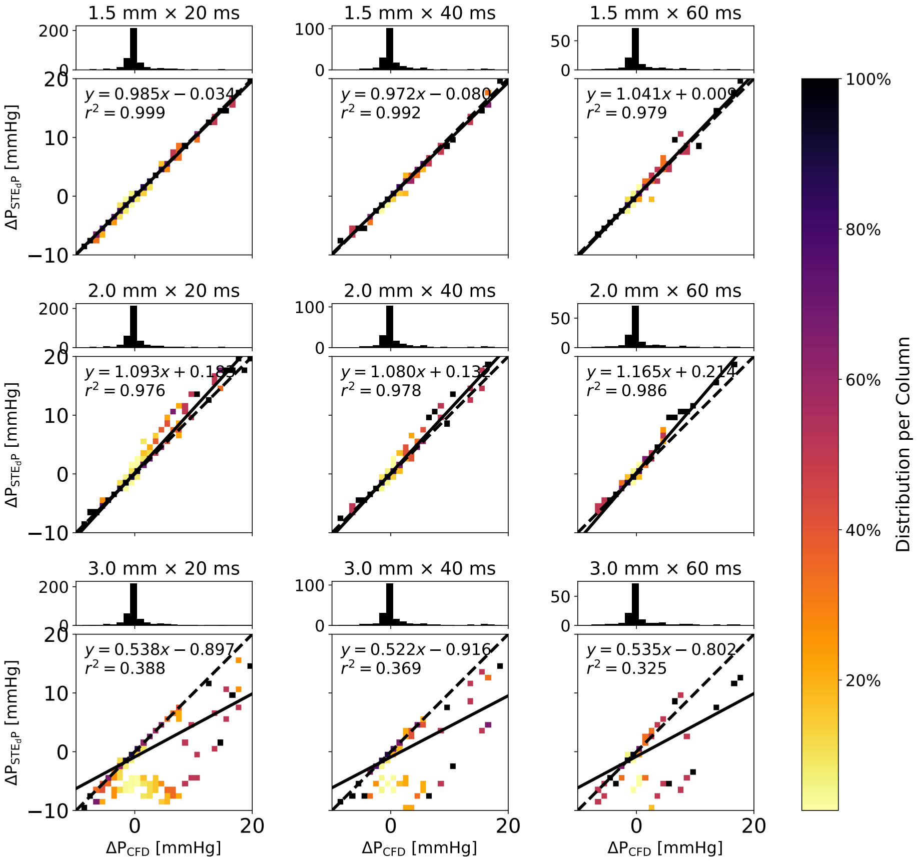
1.5 mm × 20 ms
1.5 mm × 40 ms
1.5 mm × 60 ms
2.0 mm × 20 ms
2.0 mm × 40 ms
2.0 mm × 60 ms
3.0 mm × 20 ms
3.0 mm × 40 ms
3.0 mm × 60 ms
$y = 0.985x - 0.034$
$r^2 = 0.999$
$y = 0.972x - 0.080$
$r^2 = 0.992$
$y = 1.041x + 0.009$
$r^2 = 0.979$
$r^2 = 0.976$
$r^2 = 0.978$
$r^2 = 0.986$
$y = 0.538x - 0.897$
$r^2 = 0.388$
$y = 0.522x - 0.916$
$r^2 = 0.369$
$y = 0.535x - 0.802$
$r^2 = 0.325$
ΔP_{STE_dP} [mmHg]
ΔP_{CFD} [mmHg]
Distribution per Column
100%
80%
60%
40%
20%

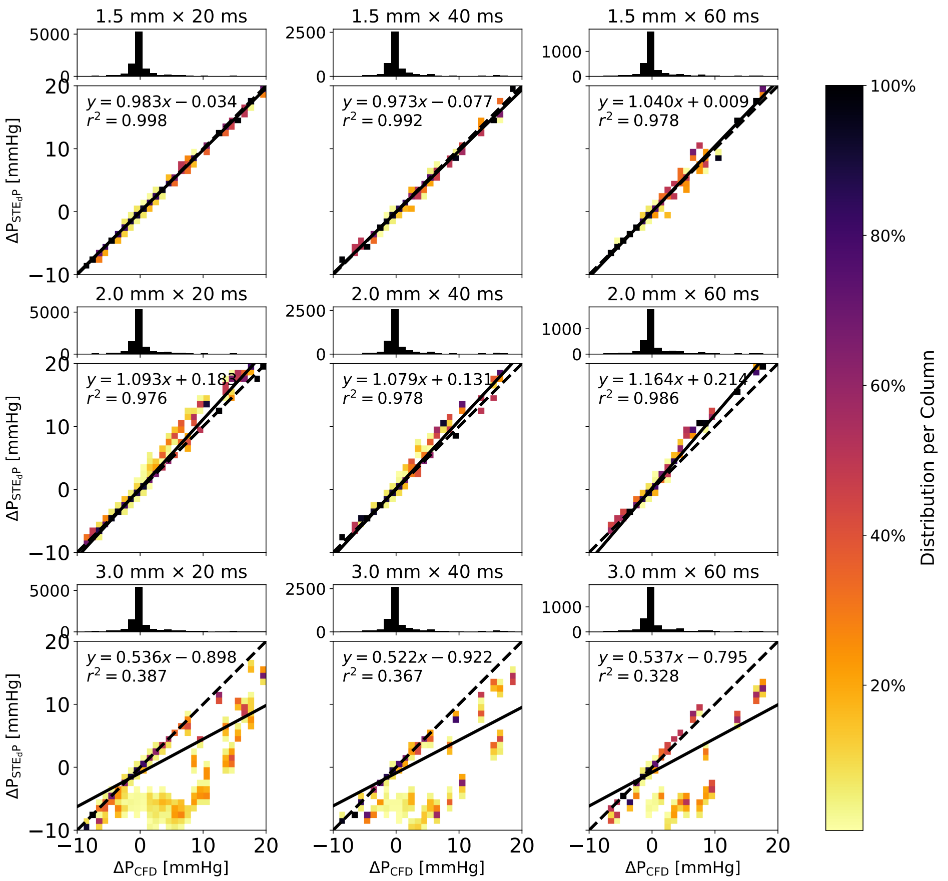
1.5 mm × 20 ms
1.5 mm × 40 ms
1.5 mm × 60 ms
$y = 0.983x - 0.034$
$r^2 = 0.998$
$y = 0.973x - 0.077$
$r^2 = 0.992$
$y = 1.040x + 0.009$
$r^2 = 0.978$
2.0 mm × 20 ms
2.0 mm × 40 ms
2.0 mm × 60 ms
$y = 1.093x + 0.183$
$r^2 = 0.976$
$y = 1.079x + 0.131$
$r^2 = 0.978$
$y = 1.164x + 0.214$
$r^2 = 0.986$
3.0 mm × 20 ms
3.0 mm × 40 ms
3.0 mm × 60 ms
$y = 0.536x - 0.898$
$r^2 = 0.387$
$y = 0.522x - 0.922$
$r^2 = 0.367$
$y = 0.537x - 0.795$
$r^2 = 0.328$
ΔP_{STE_dP} [mmHg]
ΔP_{CFD} [mmHg]
Distribution per Column
100%
80%
60%
40%
20%

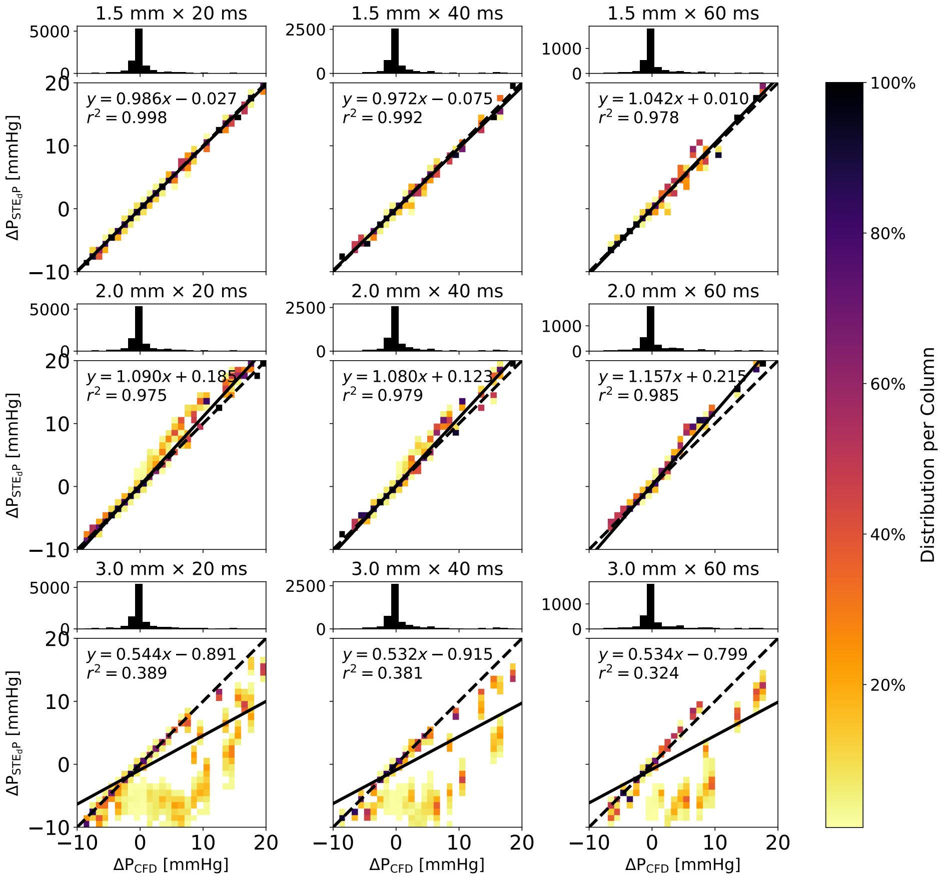
1.5 mm × 20 ms
1.5 mm × 40 ms
1.5 mm × 60 ms
$y = 0.986x - 0.027$
$r^2 = 0.998$
$y = 0.972x - 0.075$
$r^2 = 0.992$
$y = 1.042x + 0.010$
$r^2 = 0.978$
2.0 mm × 20 ms
2.0 mm × 40 ms
2.0 mm × 60 ms
$y = 1.090x + 0.185$
$r^2 = 0.975$
$y = 1.080x + 0.123$
$r^2 = 0.979$
$y = 1.157x + 0.215$
$r^2 = 0.985$
3.0 mm × 20 ms
3.0 mm × 40 ms
3.0 mm × 60 ms
$y = 0.544x - 0.891$
$r^2 = 0.389$
$y = 0.532x - 0.915$
$r^2 = 0.381$
$y = 0.534x - 0.799$
$r^2 = 0.324$
ΔP_{STE_dP} [mmHg]
ΔP_{CFD} [mmHg]
Distribution per Column
100%
80%
60%
40%
20%

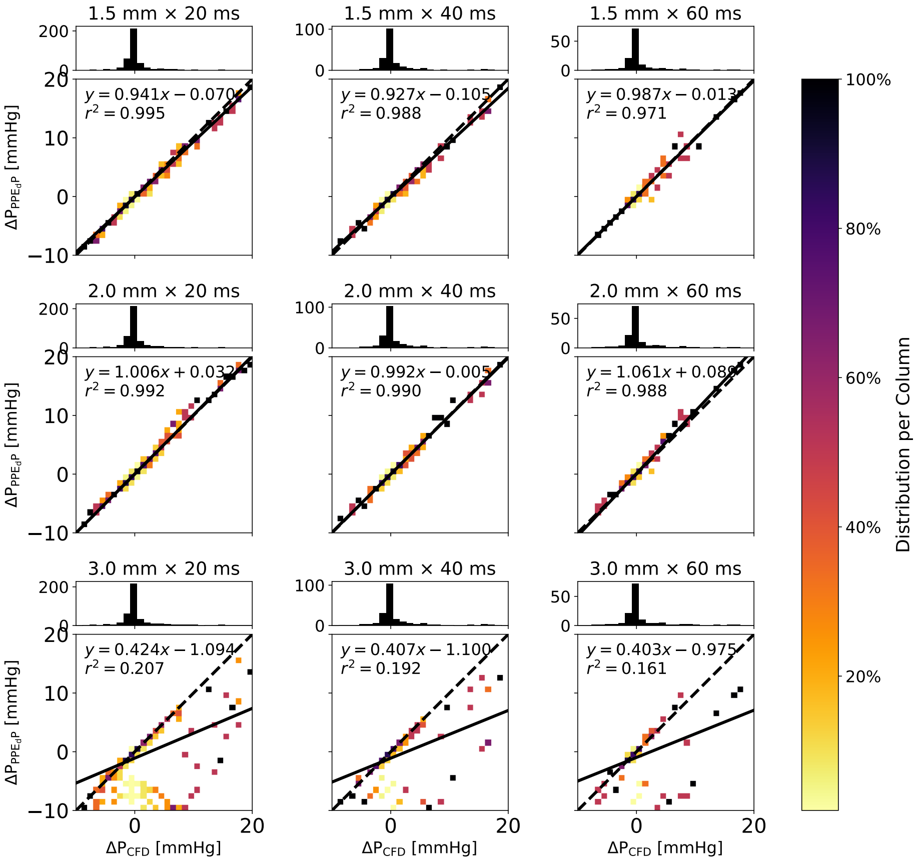
1.5 mm × 20 ms
1.5 mm × 40 ms
1.5 mm × 60 ms
y = 0.941x − 0.070
r² = 0.995
y = 0.927x − 0.105
r² = 0.988
y = 0.987x − 0.013
r² = 0.971
2.0 mm × 20 ms
2.0 mm × 40 ms
2.0 mm × 60 ms
y = 1.006x + 0.032
r² = 0.992
y = 0.992x − 0.005
r² = 0.990
y = 1.061x + 0.080
r² = 0.988
3.0 mm × 20 ms
3.0 mm × 40 ms
3.0 mm × 60 ms
y = 0.424x − 1.094
r² = 0.207
y = 0.407x − 1.100
r² = 0.192
y = 0.403x − 0.975
r² = 0.161
ΔP_PPEdP [mmHg]
ΔP_CFD [mmHg]
Distribution per Column
100%
80%
60%
40%
20%

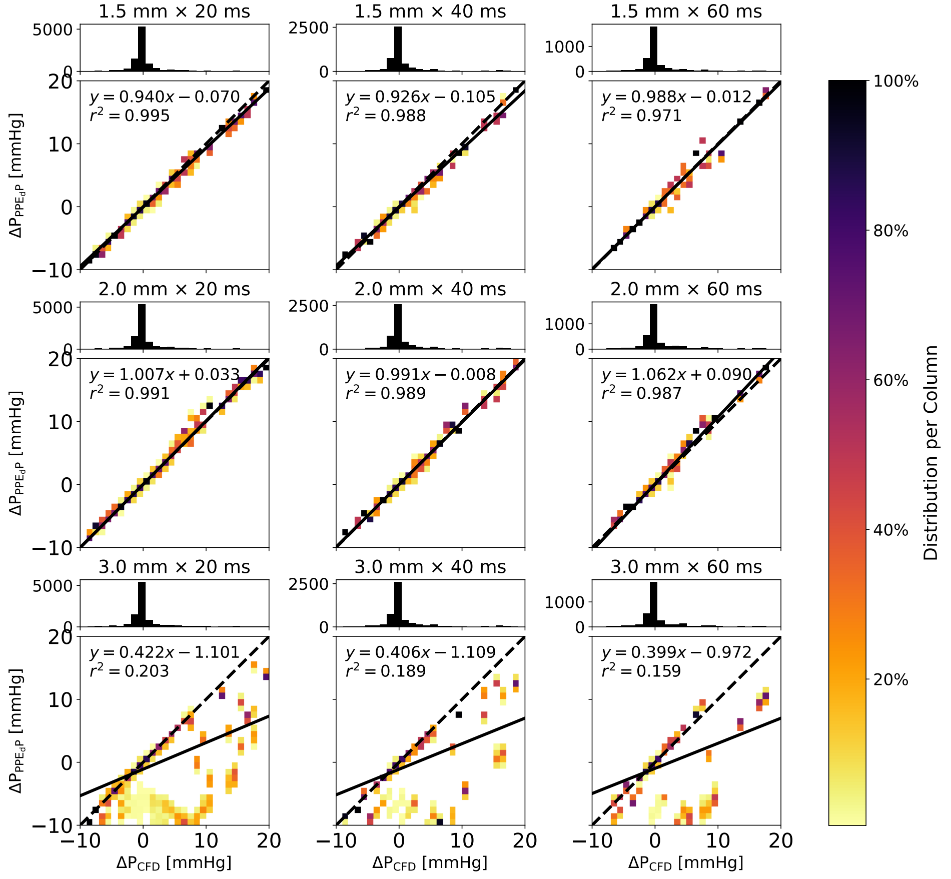
1.5 mm × 20 ms
1.5 mm × 40 ms
1.5 mm × 60 ms
y = 0.940x − 0.070
r² = 0.995
y = 0.926x − 0.105
r² = 0.988
y = 0.988x − 0.012
r² = 0.971
2.0 mm × 20 ms
2.0 mm × 40 ms
2.0 mm × 60 ms
y = 1.007x + 0.033
r² = 0.991
y = 0.991x − 0.008
r² = 0.989
y = 1.062x + 0.090
r² = 0.987
3.0 mm × 20 ms
3.0 mm × 40 ms
3.0 mm × 60 ms
y = 0.422x − 1.101
r² = 0.203
y = 0.406x − 1.109
r² = 0.189
y = 0.399x − 0.972
r² = 0.159
ΔP_PPEdP [mmHg]
ΔP_CFD [mmHg]
Distribution per Column
100%
80%
60%
40%
20%

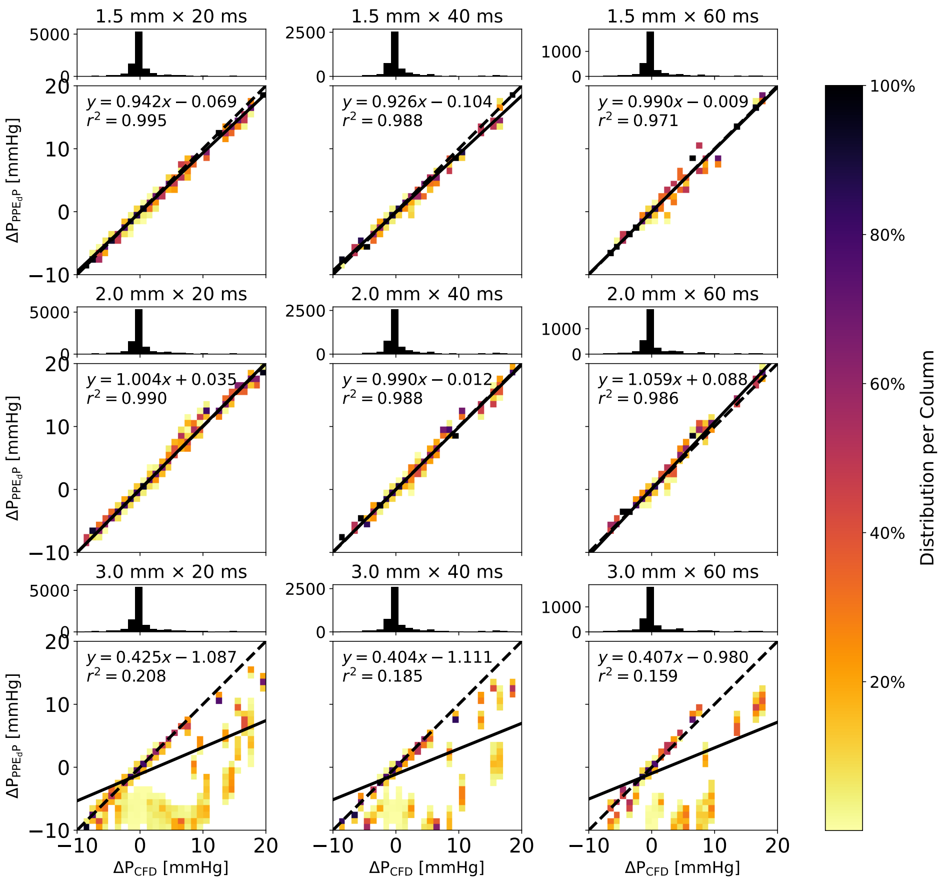

1.5 mm × 20 ms
1.5 mm × 40 ms
1.5 mm × 60 ms
y = 0.942x − 0.069
r² = 0.995
y = 0.926x − 0.104
r² = 0.988
y = 0.990x − 0.009
r² = 0.971
2.0 mm × 20 ms
2.0 mm × 40 ms
2.0 mm × 60 ms
y = 1.004x + 0.035
r² = 0.990
y = 0.990x − 0.012
r² = 0.988
y = 1.059x + 0.088
r² = 0.986
3.0 mm × 20 ms
3.0 mm × 40 ms
3.0 mm × 60 ms
y = 0.425x − 1.087
r² = 0.208
y = 0.404x − 1.111
r² = 0.185
y = 0.407x − 0.980
r² = 0.159
ΔP_PPEdP [mmHg]
ΔP_CFD [mmHg]
Distribution per Column
100%
80%
60%
40%
20%

1.6 Pressure Drop Figures

1.6.1 vWERP

FL1

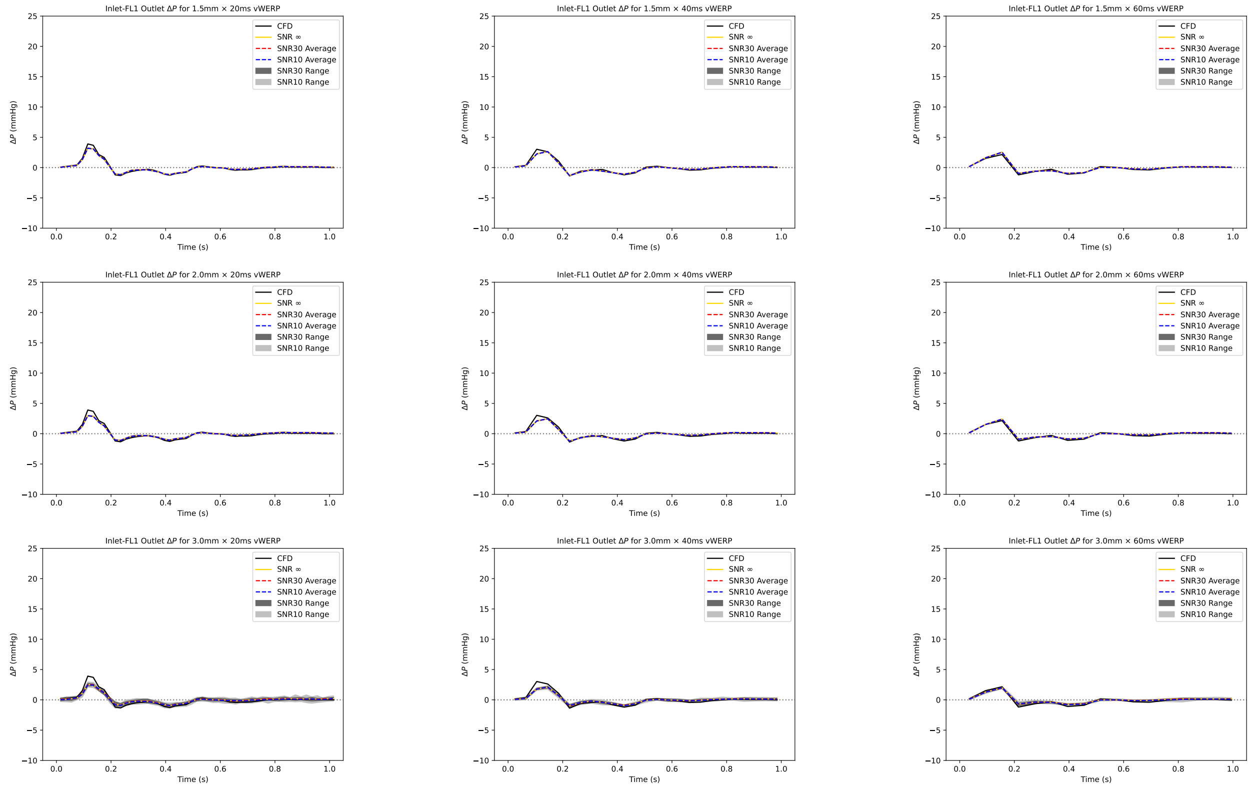

FL2

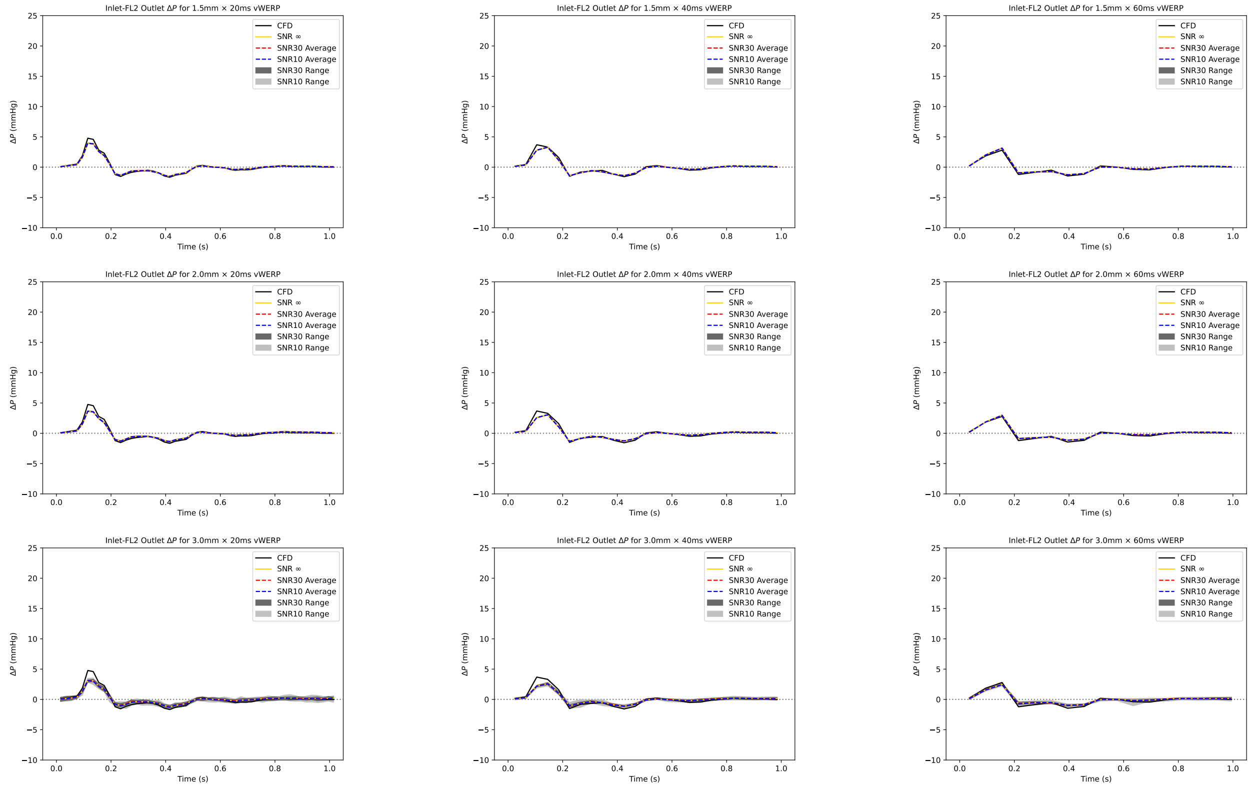

FL3

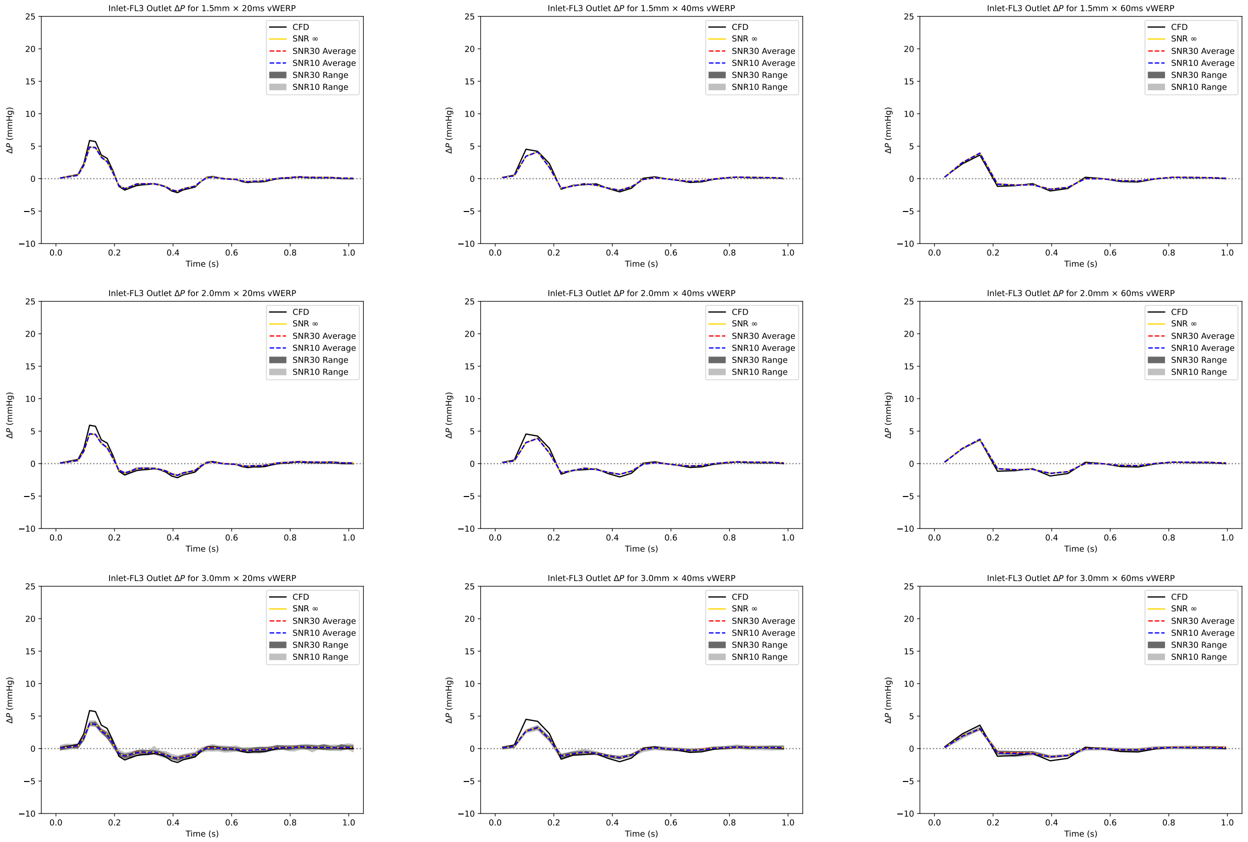
Inlet-FL3 Outlet ΔP for 1.5mm × 20ms vWERP
Inlet-FL3 Outlet ΔP for 1.5mm × 40ms vWERP
Inlet-FL3 Outlet ΔP for 1.5mm × 60ms vWERP
Inlet-FL3 Outlet ΔP for 2.0mm × 20ms vWERP
Inlet-FL3 Outlet ΔP for 2.0mm × 40ms vWERP
Inlet-FL3 Outlet ΔP for 2.0mm × 60ms vWERP
Inlet-FL3 Outlet ΔP for 3.0mm × 20ms vWERP
Inlet-FL3 Outlet ΔP for 3.0mm × 40ms vWERP
Inlet-FL3 Outlet ΔP for 3.0mm × 60ms vWERP
CFD
SNR ∞
SNR30 Average
SNR10 Average
SNR30 Range
SNR10 Range
ΔP (mmHg)
Time (s)

FL4

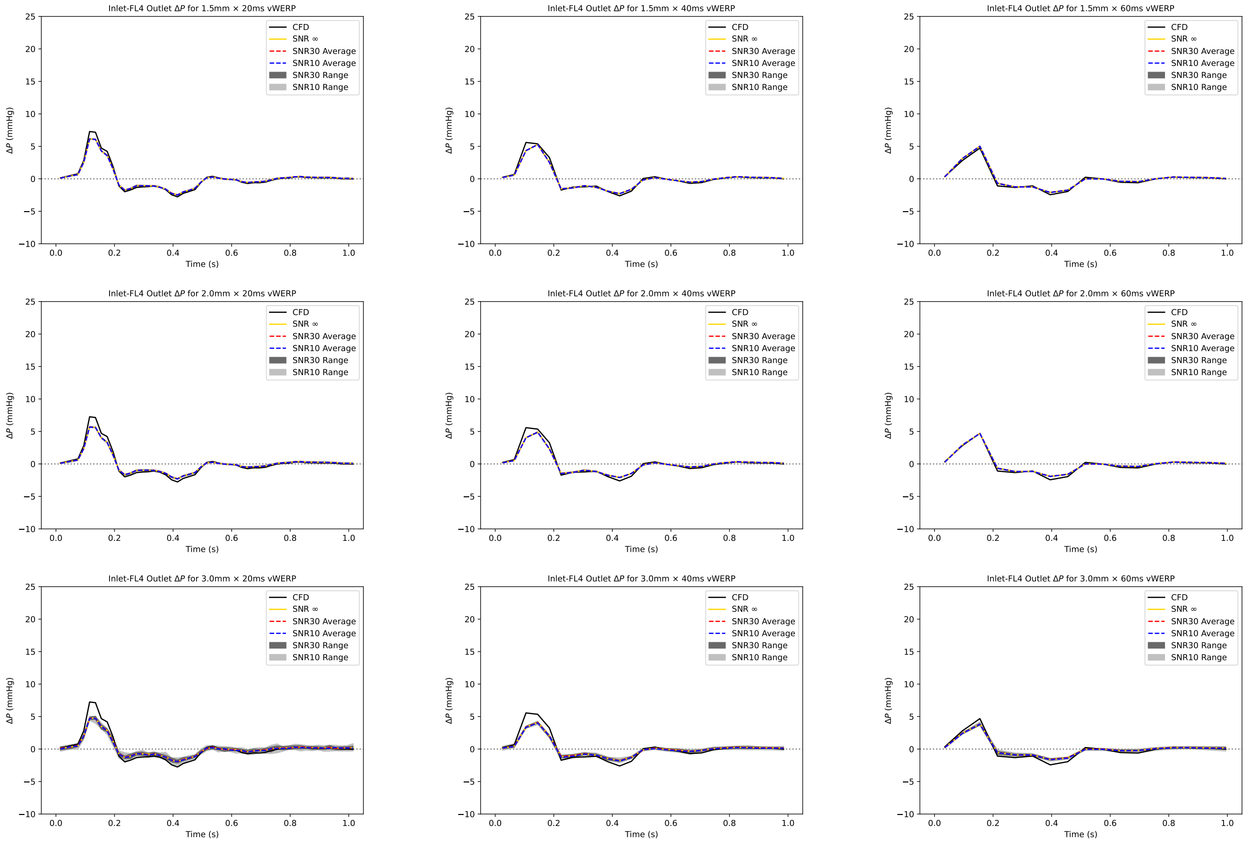
Inlet-FL4 Outlet ΔP for 1.5mm × 20ms vWERP
Inlet-FL4 Outlet ΔP for 1.5mm × 40ms vWERP
Inlet-FL4 Outlet ΔP for 1.5mm × 60ms vWERP
Inlet-FL4 Outlet ΔP for 2.0mm × 20ms vWERP
Inlet-FL4 Outlet ΔP for 2.0mm × 40ms vWERP
Inlet-FL4 Outlet ΔP for 2.0mm × 60ms vWERP
Inlet-FL4 Outlet ΔP for 3.0mm × 20ms vWERP
Inlet-FL4 Outlet ΔP for 3.0mm × 40ms vWERP
Inlet-FL4 Outlet ΔP for 3.0mm × 60ms vWERP
CFD
SNR ∞
SNR30 Average
SNR10 Average
SNR30 Range
SNR10 Range
ΔP (mmHg)
Time (s)

TL1

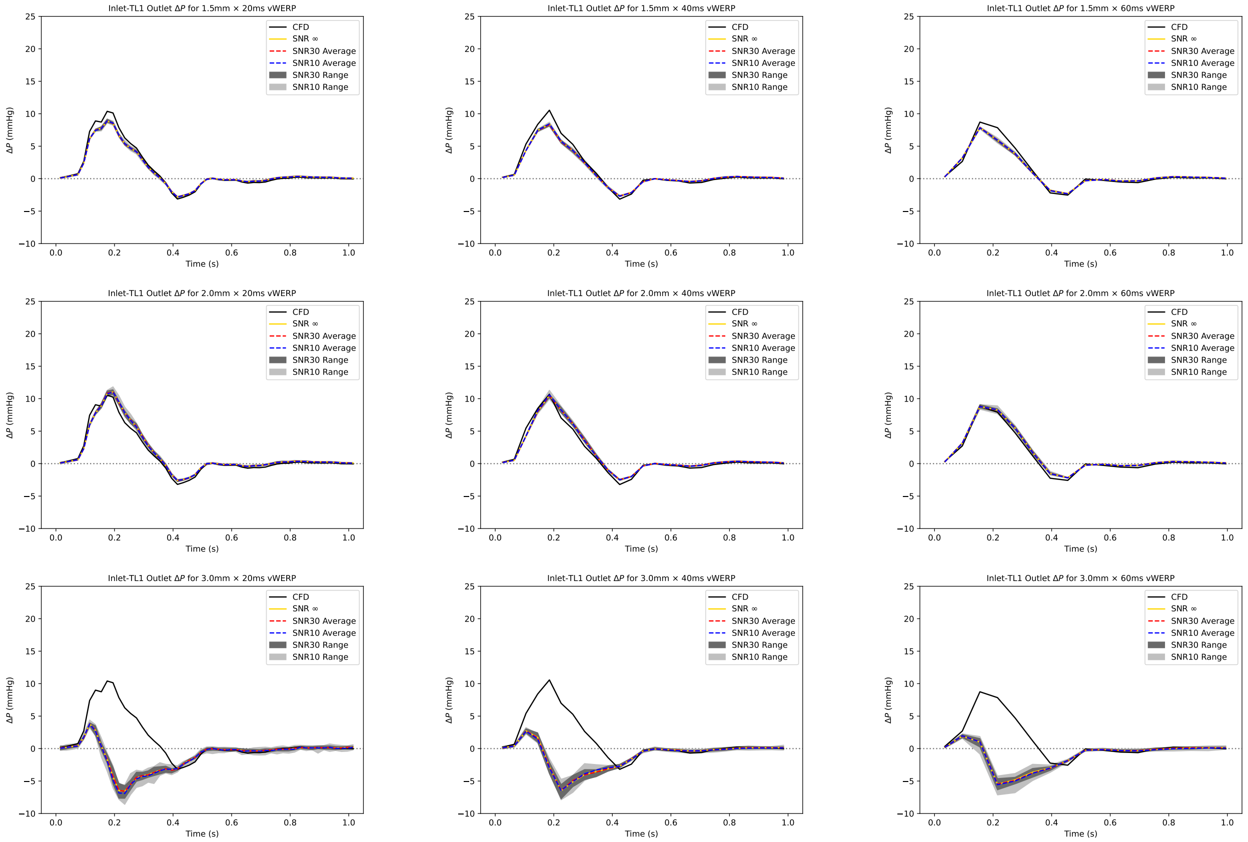
Inlet-TL1 Outlet ΔP for 1.5mm × 20ms vWERP
Inlet-TL1 Outlet ΔP for 1.5mm × 40ms vWERP
Inlet-TL1 Outlet ΔP for 1.5mm × 60ms vWERP
Inlet-TL1 Outlet ΔP for 2.0mm × 20ms vWERP
Inlet-TL1 Outlet ΔP for 2.0mm × 40ms vWERP
Inlet-TL1 Outlet ΔP for 2.0mm × 60ms vWERP
Inlet-TL1 Outlet ΔP for 3.0mm × 20ms vWERP
Inlet-TL1 Outlet ΔP for 3.0mm × 40ms vWERP
Inlet-TL1 Outlet ΔP for 3.0mm × 60ms vWERP
CFD
SNR ∞
SNR30 Average
SNR10 Average
SNR30 Range
SNR10 Range
ΔP (mmHg)
Time (s)

TL2

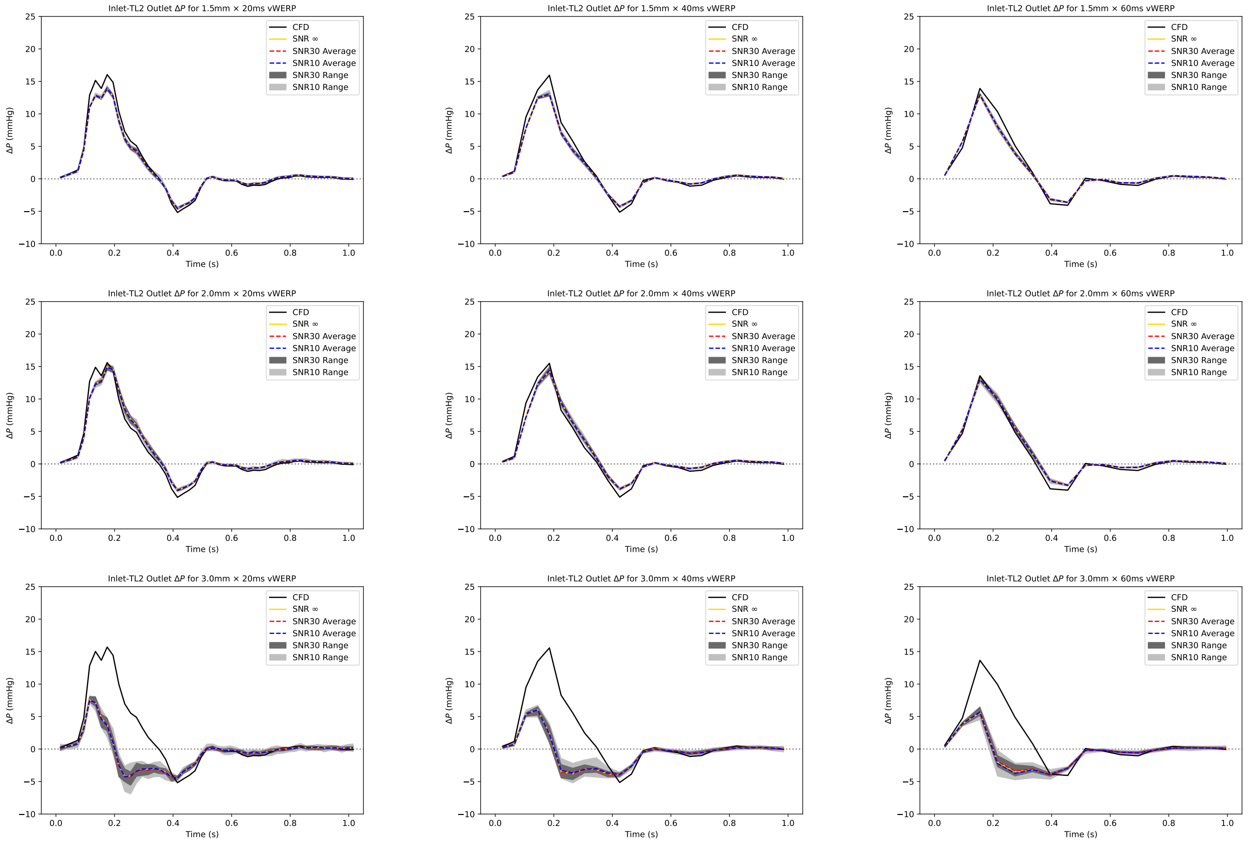
Inlet-TL2 Outlet ΔP for 1.5mm × 20ms vWERP
Inlet-TL2 Outlet ΔP for 1.5mm × 40ms vWERP
Inlet-TL2 Outlet ΔP for 1.5mm × 60ms vWERP
Inlet-TL2 Outlet ΔP for 2.0mm × 20ms vWERP
Inlet-TL2 Outlet ΔP for 2.0mm × 40ms vWERP
Inlet-TL2 Outlet ΔP for 2.0mm × 60ms vWERP
Inlet-TL2 Outlet ΔP for 3.0mm × 20ms vWERP
Inlet-TL2 Outlet ΔP for 3.0mm × 40ms vWERP
Inlet-TL2 Outlet ΔP for 3.0mm × 60ms vWERP
CFD
SNR ∞
SNR30 Average
SNR10 Average
SNR30 Range
SNR10 Range
ΔP (mmHg)
Time (s)

TL3

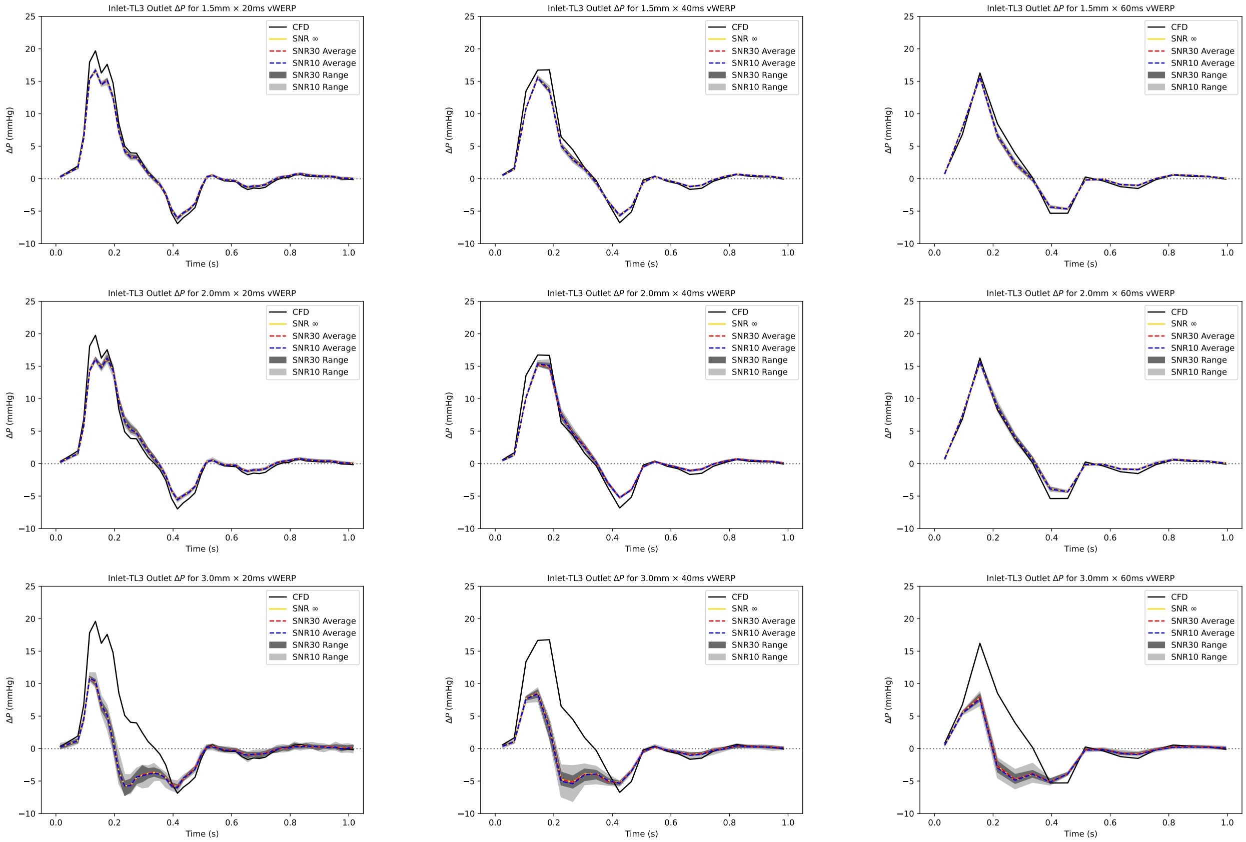
Inlet-TL3 Outlet ΔP for 1.5mm × 20ms vWERP
Inlet-TL3 Outlet ΔP for 1.5mm × 40ms vWERP
Inlet-TL3 Outlet ΔP for 1.5mm × 60ms vWERP
Inlet-TL3 Outlet ΔP for 2.0mm × 20ms vWERP
Inlet-TL3 Outlet ΔP for 2.0mm × 40ms vWERP
Inlet-TL3 Outlet ΔP for 2.0mm × 60ms vWERP
Inlet-TL3 Outlet ΔP for 3.0mm × 20ms vWERP
Inlet-TL3 Outlet ΔP for 3.0mm × 40ms vWERP
Inlet-TL3 Outlet ΔP for 3.0mm × 60ms vWERP
CFD
SNR ∞
SNR30 Average
SNR10 Average
SNR30 Range
SNR10 Range
ΔP (mmHg)
Time (s)

TL4

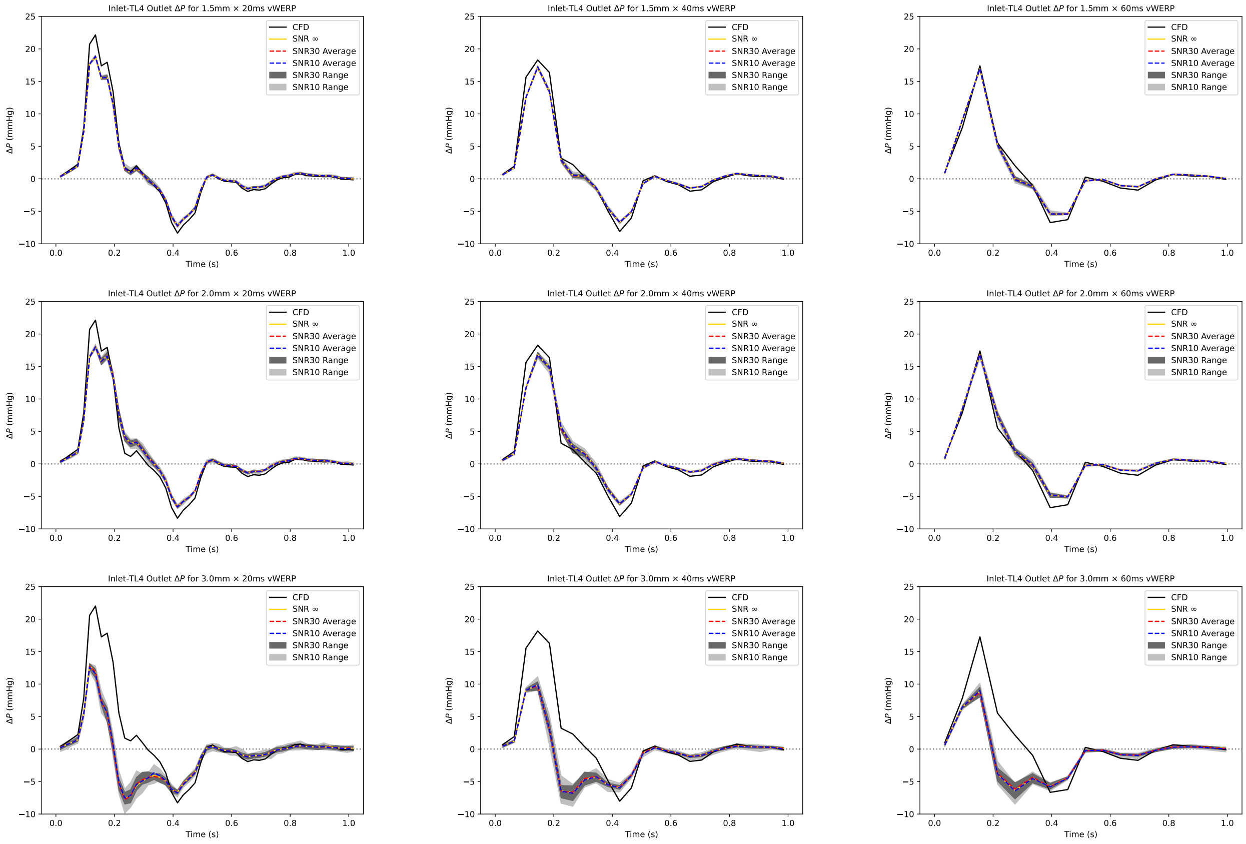
Inlet-TL4 Outlet ΔP for 1.5mm × 20ms vWERP
Inlet-TL4 Outlet ΔP for 1.5mm × 40ms vWERP
Inlet-TL4 Outlet ΔP for 1.5mm × 60ms vWERP
Inlet-TL4 Outlet ΔP for 2.0mm × 20ms vWERP
Inlet-TL4 Outlet ΔP for 2.0mm × 40ms vWERP
Inlet-TL4 Outlet ΔP for 2.0mm × 60ms vWERP
Inlet-TL4 Outlet ΔP for 3.0mm × 20ms vWERP
Inlet-TL4 Outlet ΔP for 3.0mm × 40ms vWERP
Inlet-TL4 Outlet ΔP for 3.0mm × 60ms vWERP
CFD
SNR ∞
SNR30 Average
SNR10 Average
SNR30 Range
SNR10 Range
ΔP (mmHg)
Time (s)

1.6.2 STE

FL1

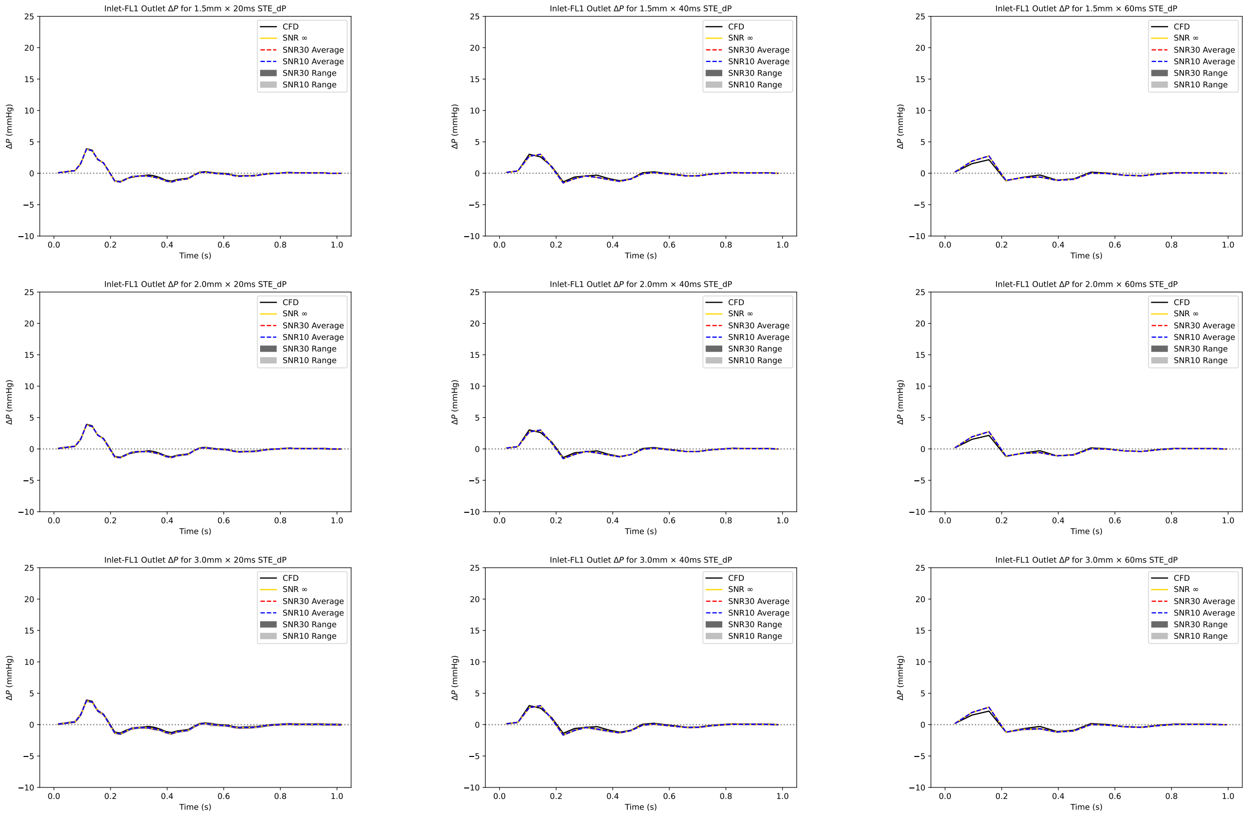

FL2

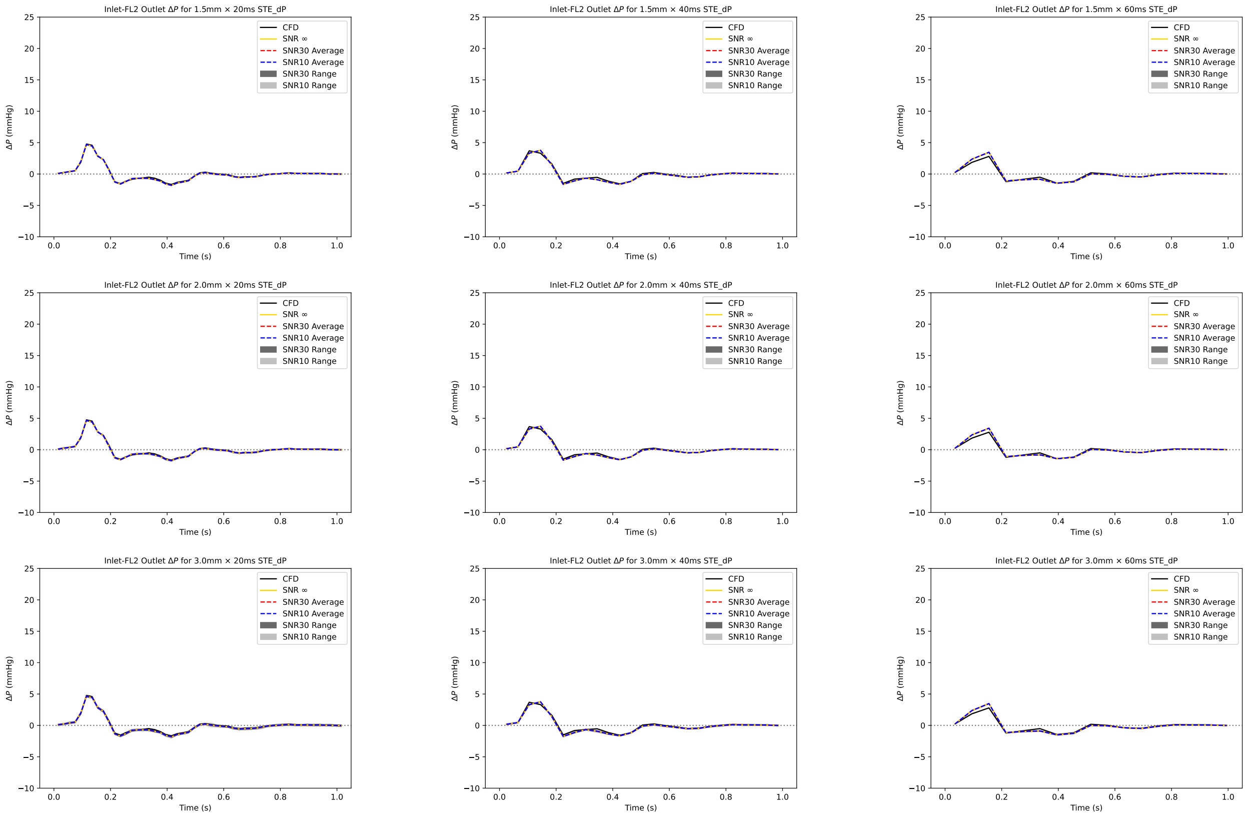

FL3

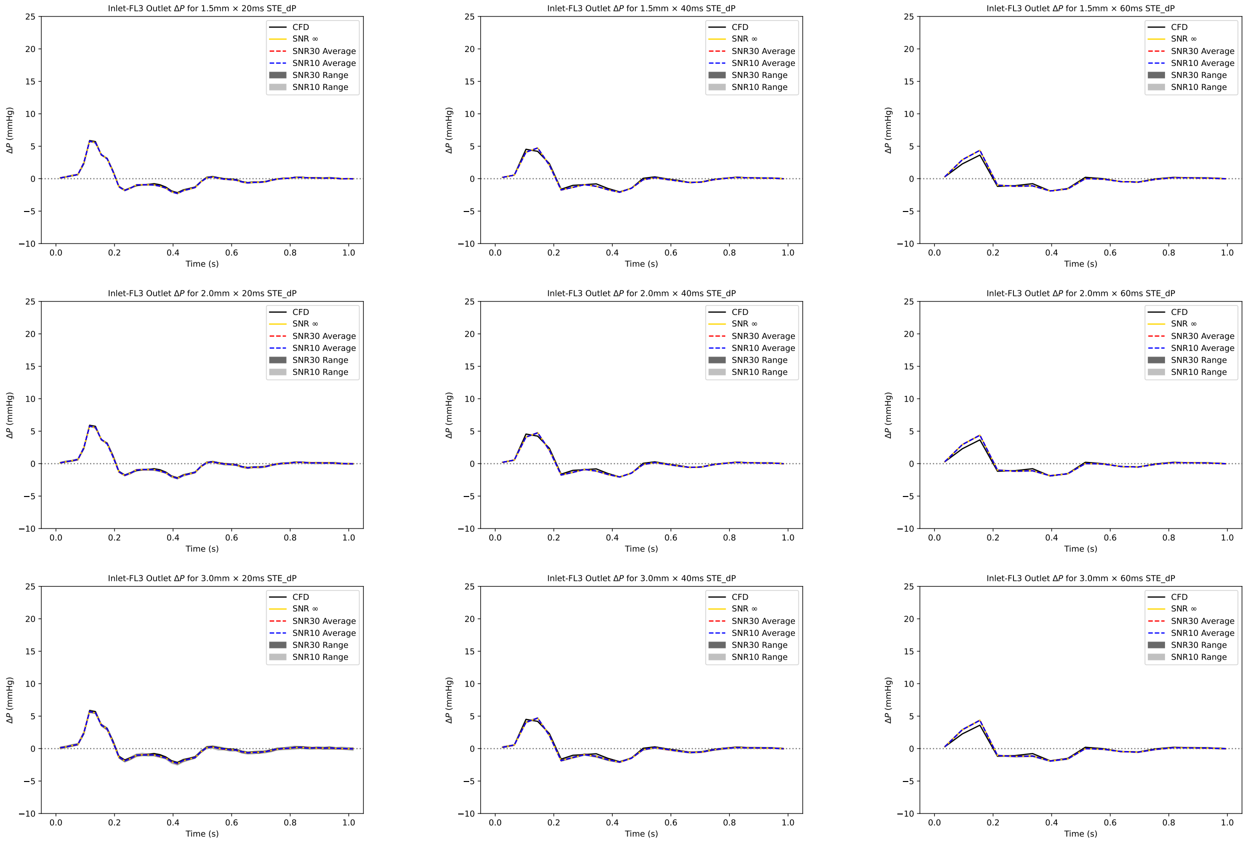
Inlet-FL3 Outlet ΔP for 1.5mm × 20ms STE_dP
Inlet-FL3 Outlet ΔP for 1.5mm × 40ms STE_dP
Inlet-FL3 Outlet ΔP for 1.5mm × 60ms STE_dP
Inlet-FL3 Outlet ΔP for 2.0mm × 20ms STE_dP
Inlet-FL3 Outlet ΔP for 2.0mm × 40ms STE_dP
Inlet-FL3 Outlet ΔP for 2.0mm × 60ms STE_dP
Inlet-FL3 Outlet ΔP for 3.0mm × 20ms STE_dP
Inlet-FL3 Outlet ΔP for 3.0mm × 40ms STE_dP
Inlet-FL3 Outlet ΔP for 3.0mm × 60ms STE_dP
CFD
SNR ∞
SNR30 Average
SNR10 Average
SNR30 Range
SNR10 Range
ΔP (mmHg)
Time (s)

FL4

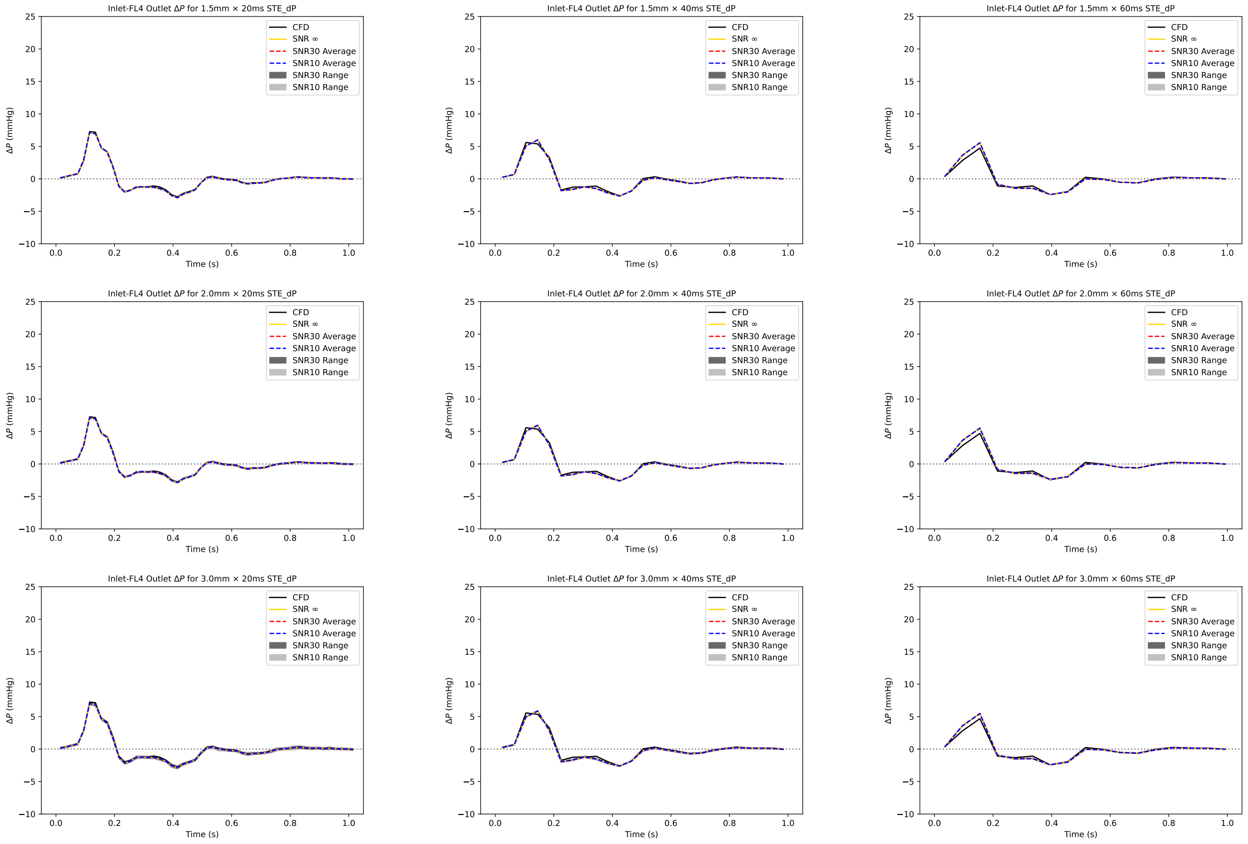
Inlet-FL4 Outlet ΔP for 1.5mm × 20ms STE_dP
Inlet-FL4 Outlet ΔP for 1.5mm × 40ms STE_dP
Inlet-FL4 Outlet ΔP for 1.5mm × 60ms STE_dP
Inlet-FL4 Outlet ΔP for 2.0mm × 20ms STE_dP
Inlet-FL4 Outlet ΔP for 2.0mm × 40ms STE_dP
Inlet-FL4 Outlet ΔP for 2.0mm × 60ms STE_dP
Inlet-FL4 Outlet ΔP for 3.0mm × 20ms STE_dP
Inlet-FL4 Outlet ΔP for 3.0mm × 40ms STE_dP
Inlet-FL4 Outlet ΔP for 3.0mm × 60ms STE_dP
CFD
SNR ∞
SNR30 Average
SNR10 Average
SNR30 Range
SNR10 Range
ΔP (mmHg)
Time (s)

TL1

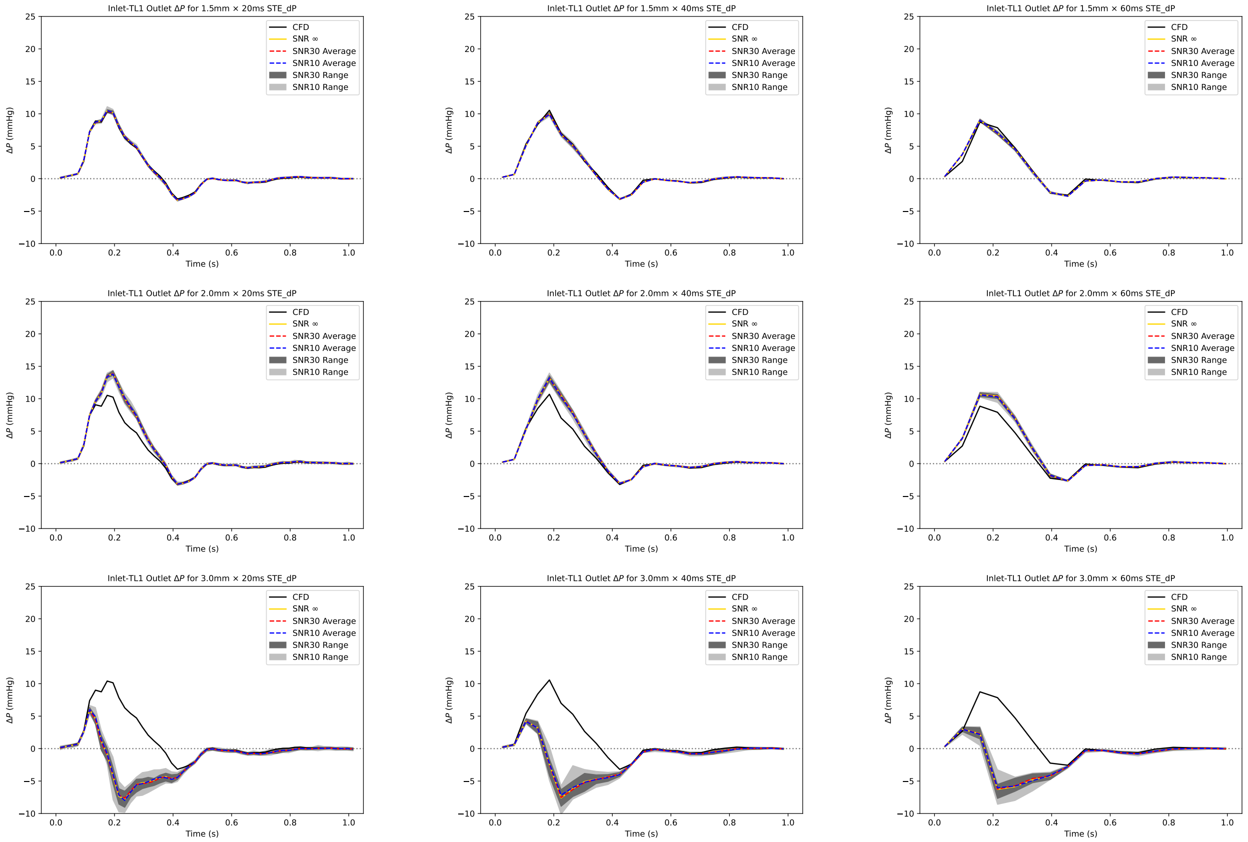

Inlet-TL1 Outlet ΔP for 1.5mm × 20ms STE_dP
Inlet-TL1 Outlet ΔP for 1.5mm × 40ms STE_dP
Inlet-TL1 Outlet ΔP for 1.5mm × 60ms STE_dP
Inlet-TL1 Outlet ΔP for 2.0mm × 20ms STE_dP
Inlet-TL1 Outlet ΔP for 2.0mm × 40ms STE_dP
Inlet-TL1 Outlet ΔP for 2.0mm × 60ms STE_dP
Inlet-TL1 Outlet ΔP for 3.0mm × 20ms STE_dP
Inlet-TL1 Outlet ΔP for 3.0mm × 40ms STE_dP
Inlet-TL1 Outlet ΔP for 3.0mm × 60ms STE_dP
CFD
SNR ∞
SNR30 Average
SNR10 Average
SNR30 Range
SNR10 Range
ΔP (mmHg)
Time (s)

TL2

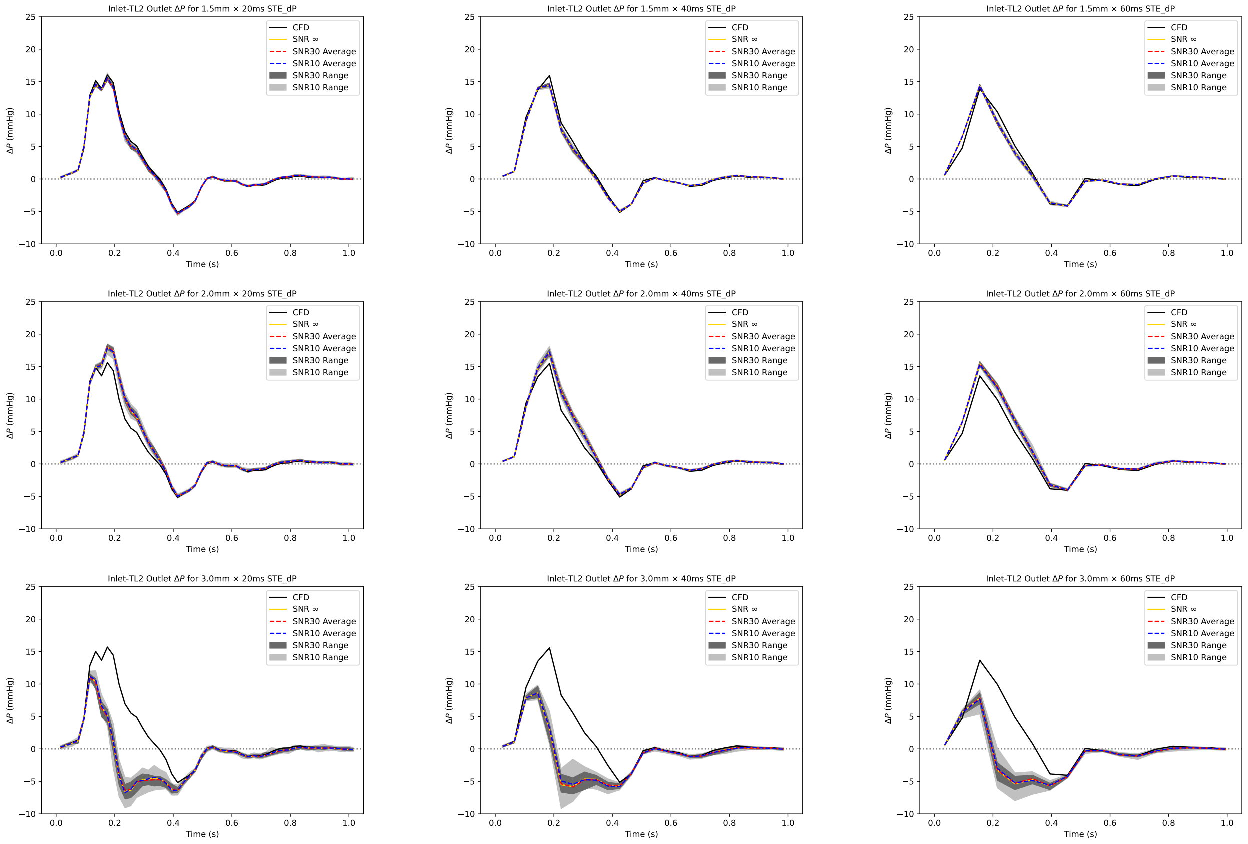

Inlet-TL2 Outlet ΔP for 1.5mm × 20ms STE_dP
Inlet-TL2 Outlet ΔP for 1.5mm × 40ms STE_dP
Inlet-TL2 Outlet ΔP for 1.5mm × 60ms STE_dP
Inlet-TL2 Outlet ΔP for 2.0mm × 20ms STE_dP
Inlet-TL2 Outlet ΔP for 2.0mm × 40ms STE_dP
Inlet-TL2 Outlet ΔP for 2.0mm × 60ms STE_dP
Inlet-TL2 Outlet ΔP for 3.0mm × 20ms STE_dP
Inlet-TL2 Outlet ΔP for 3.0mm × 40ms STE_dP
Inlet-TL2 Outlet ΔP for 3.0mm × 60ms STE_dP
CFD
SNR ∞
SNR30 Average
SNR10 Average
SNR30 Range
SNR10 Range
ΔP (mmHg)
Time (s)

TL3

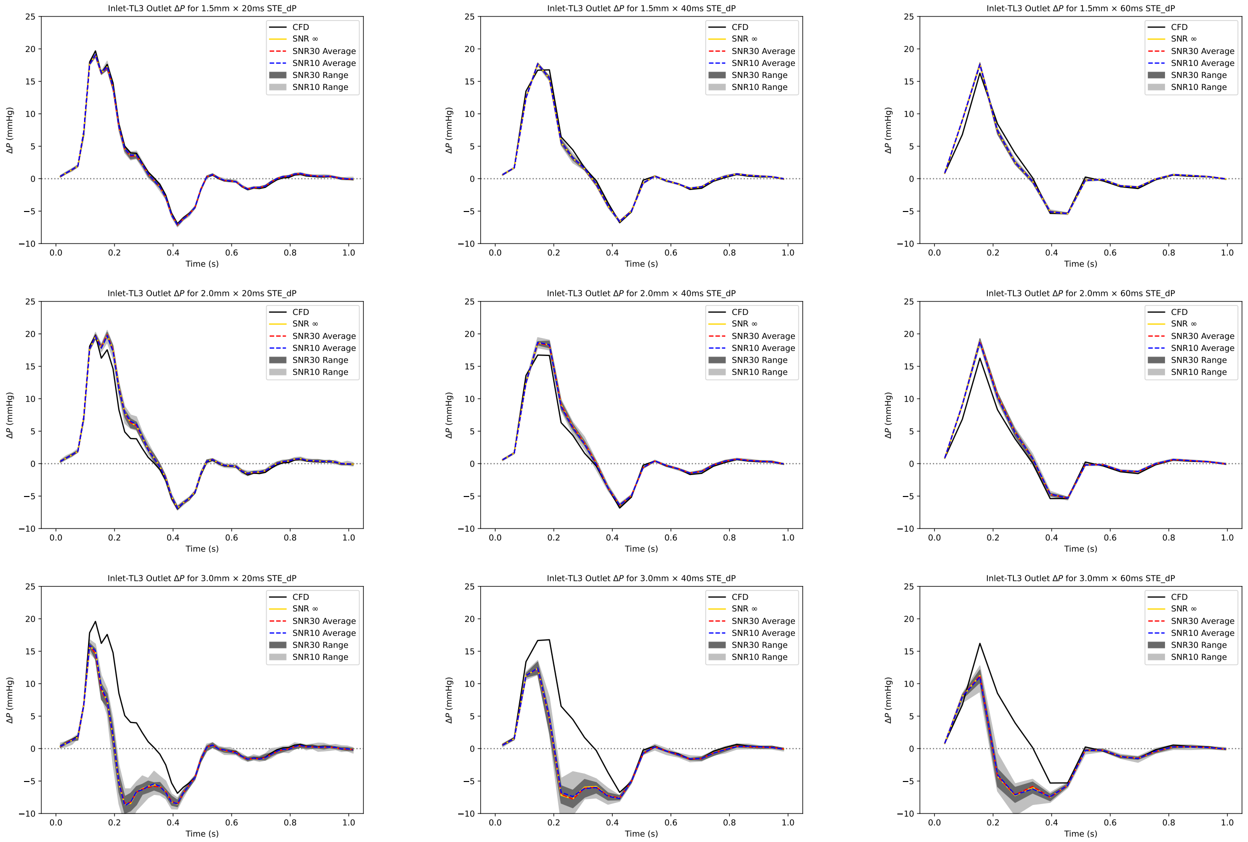
Inlet-TL3 Outlet ΔP for 1.5mm × 20ms STE_dP
Inlet-TL3 Outlet ΔP for 1.5mm × 40ms STE_dP
Inlet-TL3 Outlet ΔP for 1.5mm × 60ms STE_dP
Inlet-TL3 Outlet ΔP for 2.0mm × 20ms STE_dP
Inlet-TL3 Outlet ΔP for 2.0mm × 40ms STE_dP
Inlet-TL3 Outlet ΔP for 2.0mm × 60ms STE_dP
Inlet-TL3 Outlet ΔP for 3.0mm × 20ms STE_dP
Inlet-TL3 Outlet ΔP for 3.0mm × 40ms STE_dP
Inlet-TL3 Outlet ΔP for 3.0mm × 60ms STE_dP
CFD
SNR ∞
SNR30 Average
SNR10 Average
SNR30 Range
SNR10 Range
ΔP (mmHg)
Time (s)

TL4

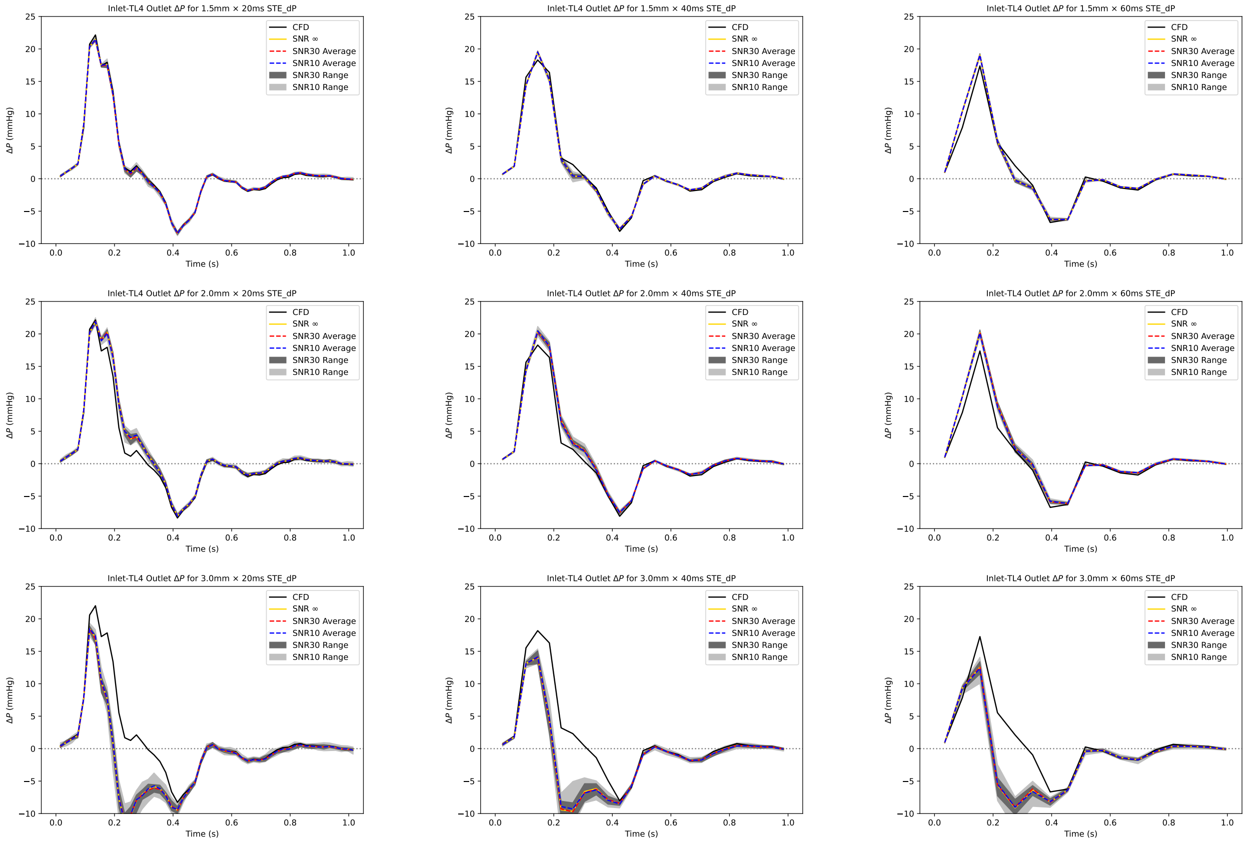
Inlet-TL4 Outlet ΔP for 1.5mm × 20ms STE_dP
Inlet-TL4 Outlet ΔP for 1.5mm × 40ms STE_dP
Inlet-TL4 Outlet ΔP for 1.5mm × 60ms STE_dP
Inlet-TL4 Outlet ΔP for 2.0mm × 20ms STE_dP
Inlet-TL4 Outlet ΔP for 2.0mm × 40ms STE_dP
Inlet-TL4 Outlet ΔP for 2.0mm × 60ms STE_dP
Inlet-TL4 Outlet ΔP for 3.0mm × 20ms STE_dP
Inlet-TL4 Outlet ΔP for 3.0mm × 40ms STE_dP
Inlet-TL4 Outlet ΔP for 3.0mm × 60ms STE_dP
CFD
SNR ∞
SNR30 Average
SNR10 Average
SNR30 Range
SNR10 Range
ΔP (mmHg)
Time (s)

1.6.3 PPE

FL1

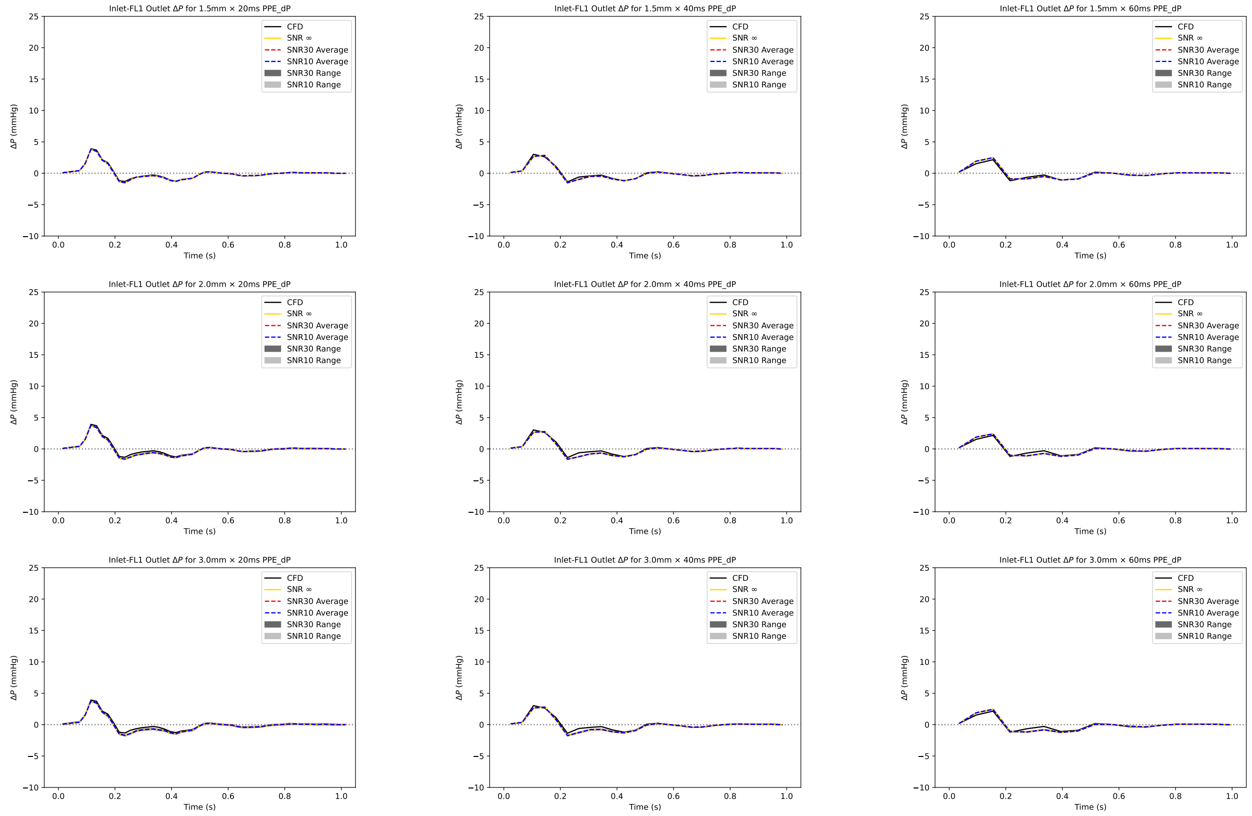

FL2

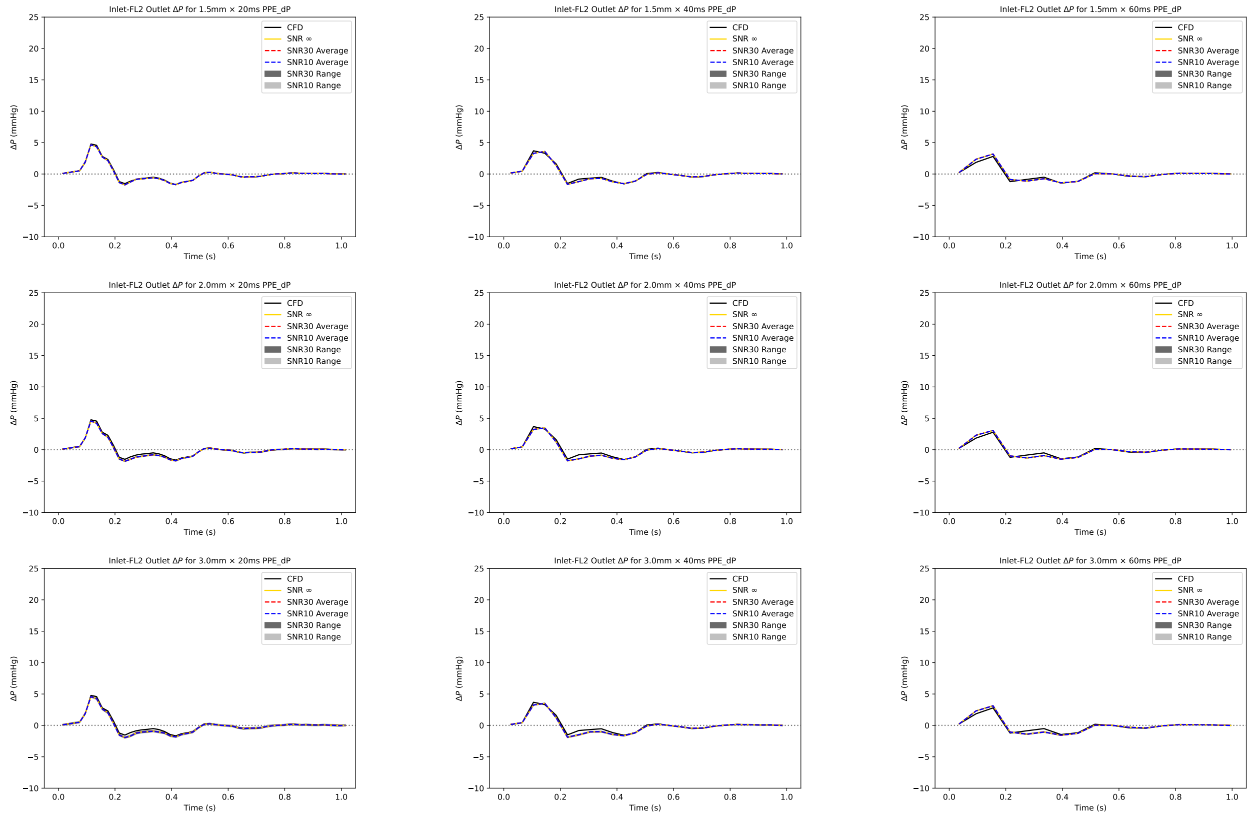

FL3

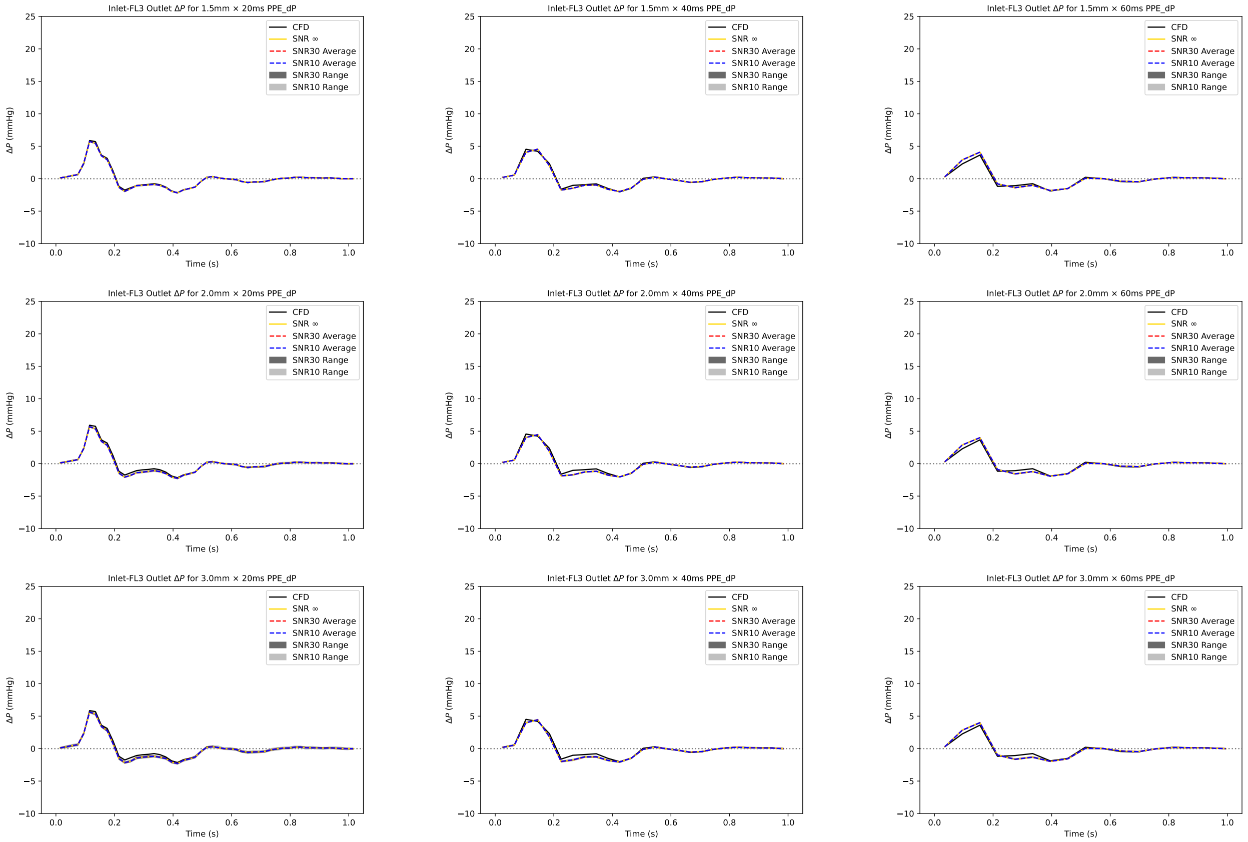
Inlet-FL3 Outlet ΔP for 1.5mm × 20ms PPE_dP
Inlet-FL3 Outlet ΔP for 1.5mm × 40ms PPE_dP
Inlet-FL3 Outlet ΔP for 1.5mm × 60ms PPE_dP
Inlet-FL3 Outlet ΔP for 2.0mm × 20ms PPE_dP
Inlet-FL3 Outlet ΔP for 2.0mm × 40ms PPE_dP
Inlet-FL3 Outlet ΔP for 2.0mm × 60ms PPE_dP
Inlet-FL3 Outlet ΔP for 3.0mm × 20ms PPE_dP
Inlet-FL3 Outlet ΔP for 3.0mm × 40ms PPE_dP
Inlet-FL3 Outlet ΔP for 3.0mm × 60ms PPE_dP
CFD
SNR ∞
SNR30 Average
SNR10 Average
SNR30 Range
SNR10 Range
ΔP (mmHg)
Time (s)

FL4

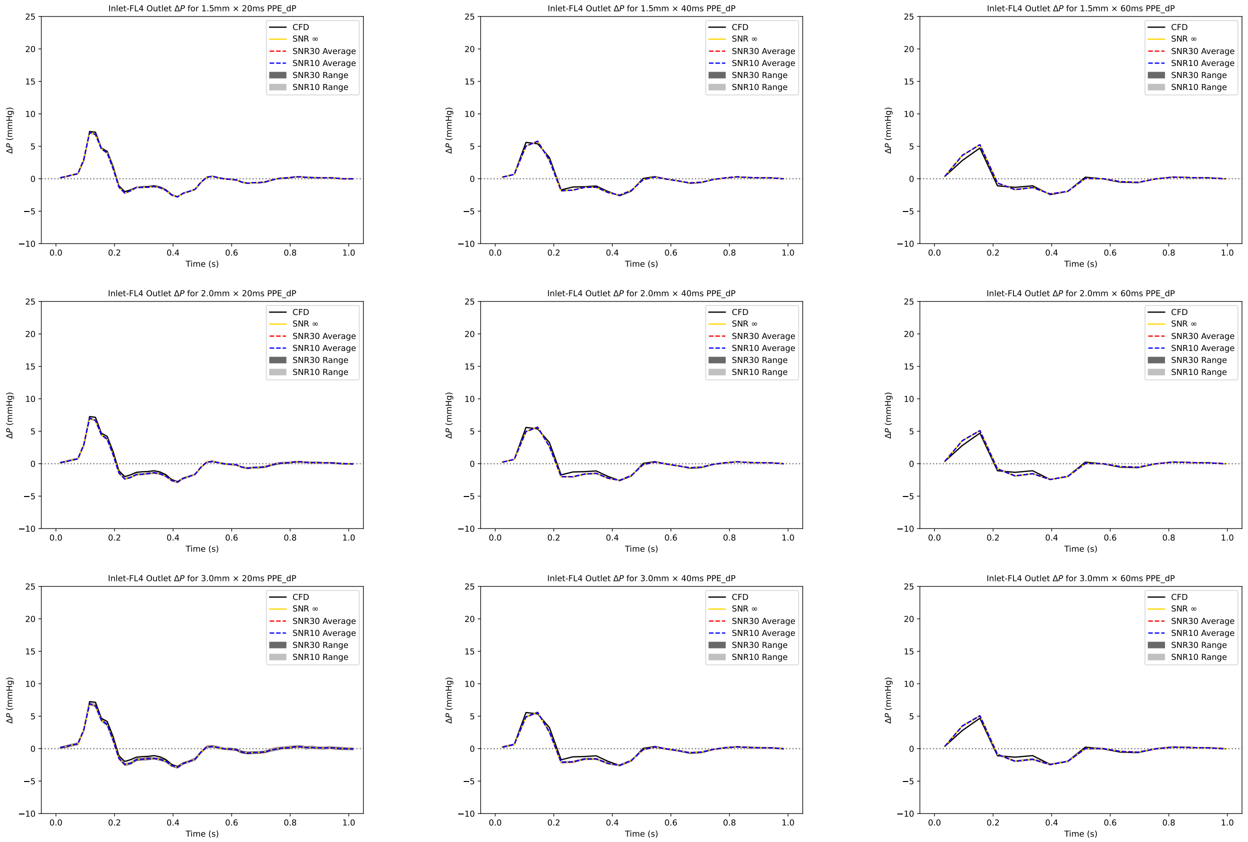
Inlet-FL4 Outlet ΔP for 1.5mm × 20ms PPE_dP
Inlet-FL4 Outlet ΔP for 1.5mm × 40ms PPE_dP
Inlet-FL4 Outlet ΔP for 1.5mm × 60ms PPE_dP
Inlet-FL4 Outlet ΔP for 2.0mm × 20ms PPE_dP
Inlet-FL4 Outlet ΔP for 2.0mm × 40ms PPE_dP
Inlet-FL4 Outlet ΔP for 2.0mm × 60ms PPE_dP
Inlet-FL4 Outlet ΔP for 3.0mm × 20ms PPE_dP
Inlet-FL4 Outlet ΔP for 3.0mm × 40ms PPE_dP
Inlet-FL4 Outlet ΔP for 3.0mm × 60ms PPE_dP
CFD
SNR ∞
SNR30 Average
SNR10 Average
SNR30 Range
SNR10 Range
ΔP (mmHg)
Time (s)

TL1

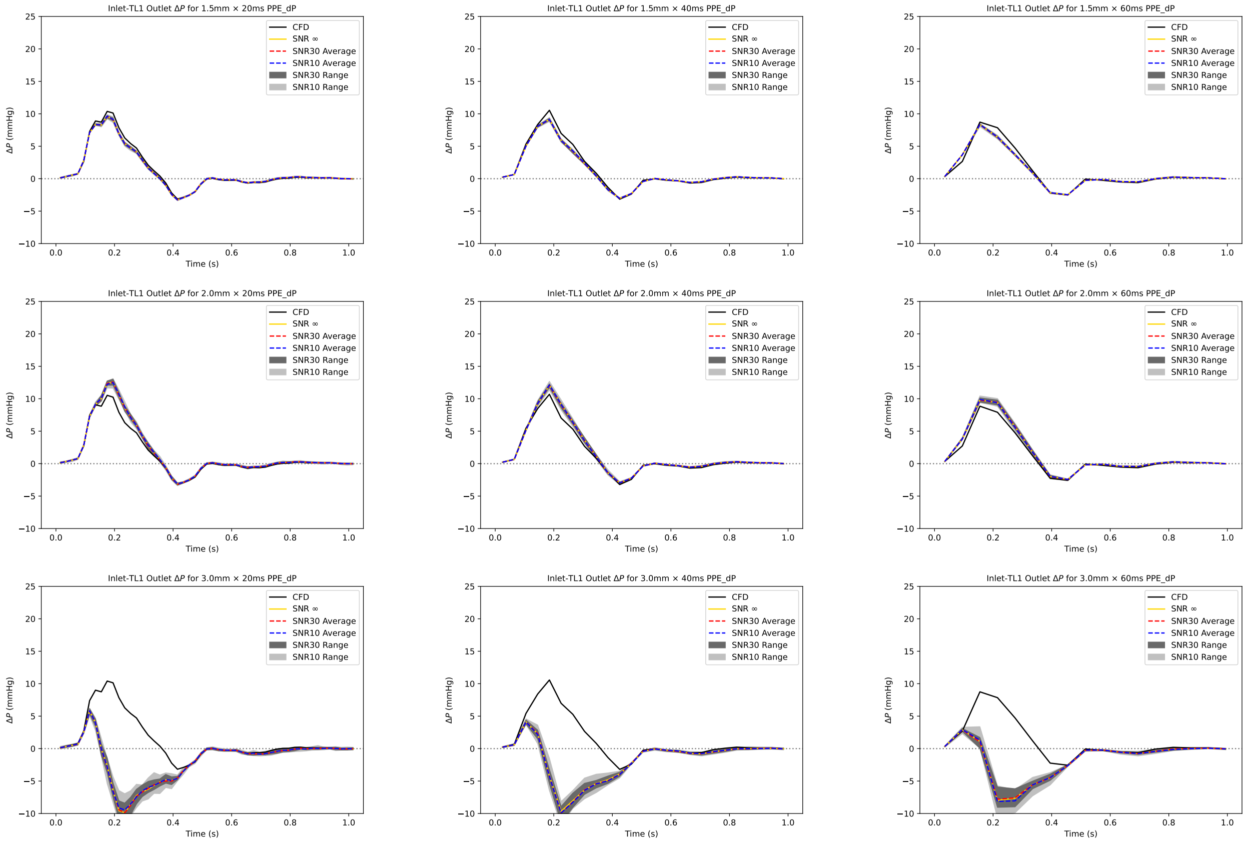
Inlet-TL1 Outlet ΔP for 1.5mm × 20ms PPE_dP
Inlet-TL1 Outlet ΔP for 1.5mm × 40ms PPE_dP
Inlet-TL1 Outlet ΔP for 1.5mm × 60ms PPE_dP
Inlet-TL1 Outlet ΔP for 2.0mm × 20ms PPE_dP
Inlet-TL1 Outlet ΔP for 2.0mm × 40ms PPE_dP
Inlet-TL1 Outlet ΔP for 2.0mm × 60ms PPE_dP
Inlet-TL1 Outlet ΔP for 3.0mm × 20ms PPE_dP
Inlet-TL1 Outlet ΔP for 3.0mm × 40ms PPE_dP
Inlet-TL1 Outlet ΔP for 3.0mm × 60ms PPE_dP
CFD
SNR ∞
SNR30 Average
SNR10 Average
SNR30 Range
SNR10 Range
ΔP (mmHg)
Time (s)

TL2

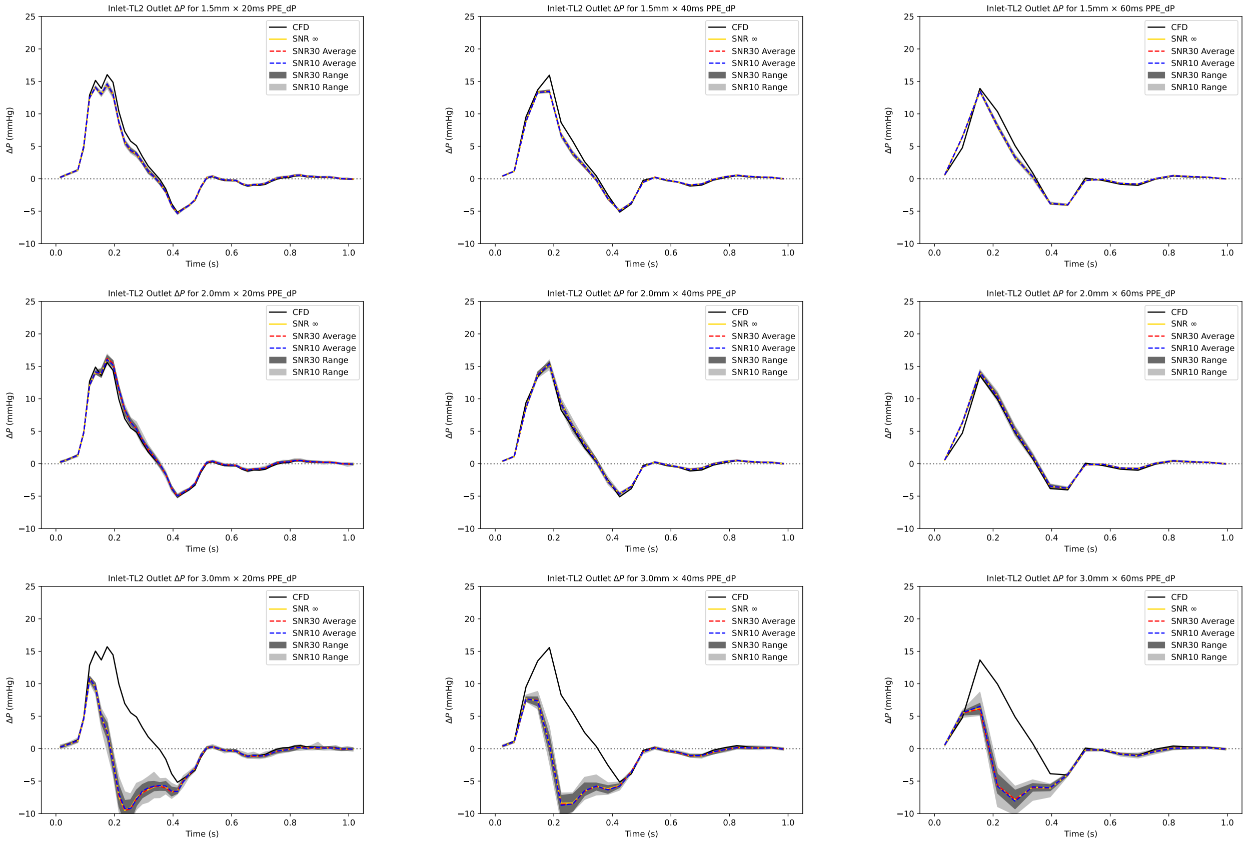
Inlet-TL2 Outlet ΔP for 1.5mm × 20ms PPE_dP
Inlet-TL2 Outlet ΔP for 1.5mm × 40ms PPE_dP
Inlet-TL2 Outlet ΔP for 1.5mm × 60ms PPE_dP
Inlet-TL2 Outlet ΔP for 2.0mm × 20ms PPE_dP
Inlet-TL2 Outlet ΔP for 2.0mm × 40ms PPE_dP
Inlet-TL2 Outlet ΔP for 2.0mm × 60ms PPE_dP
Inlet-TL2 Outlet ΔP for 3.0mm × 20ms PPE_dP
Inlet-TL2 Outlet ΔP for 3.0mm × 40ms PPE_dP
Inlet-TL2 Outlet ΔP for 3.0mm × 60ms PPE_dP
CFD
SNR ∞
SNR30 Average
SNR10 Average
SNR30 Range
SNR10 Range
ΔP (mmHg)
Time (s)

TL3

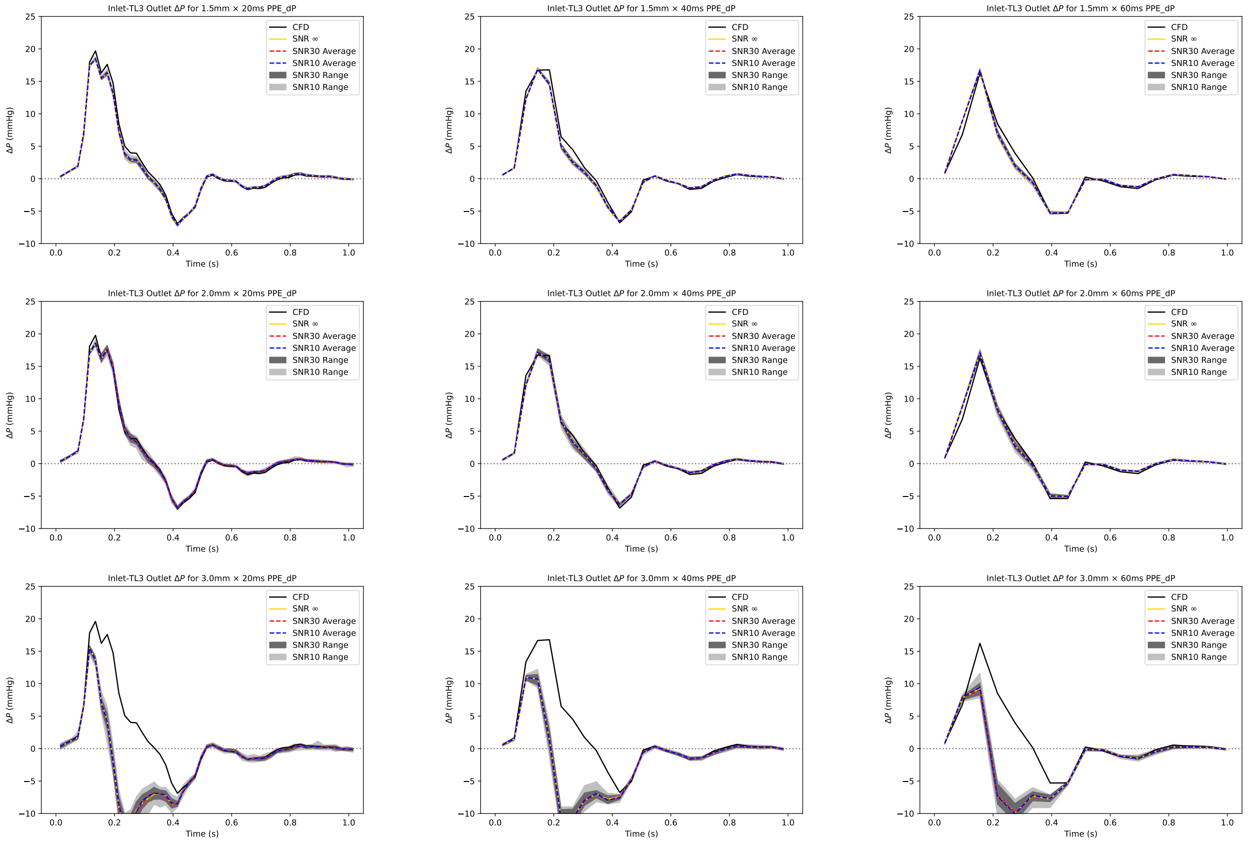
Inlet-TL3 Outlet ΔP for 1.5mm × 20ms PPE_dP
Inlet-TL3 Outlet ΔP for 1.5mm × 40ms PPE_dP
Inlet-TL3 Outlet ΔP for 1.5mm × 60ms PPE_dP
Inlet-TL3 Outlet ΔP for 2.0mm × 20ms PPE_dP
Inlet-TL3 Outlet ΔP for 2.0mm × 40ms PPE_dP
Inlet-TL3 Outlet ΔP for 2.0mm × 60ms PPE_dP
Inlet-TL3 Outlet ΔP for 3.0mm × 20ms PPE_dP
Inlet-TL3 Outlet ΔP for 3.0mm × 40ms PPE_dP
Inlet-TL3 Outlet ΔP for 3.0mm × 60ms PPE_dP
CFD
SNR ∞
SNR30 Average
SNR10 Average
SNR30 Range
SNR10 Range
ΔP (mmHg)
Time (s)

TL4

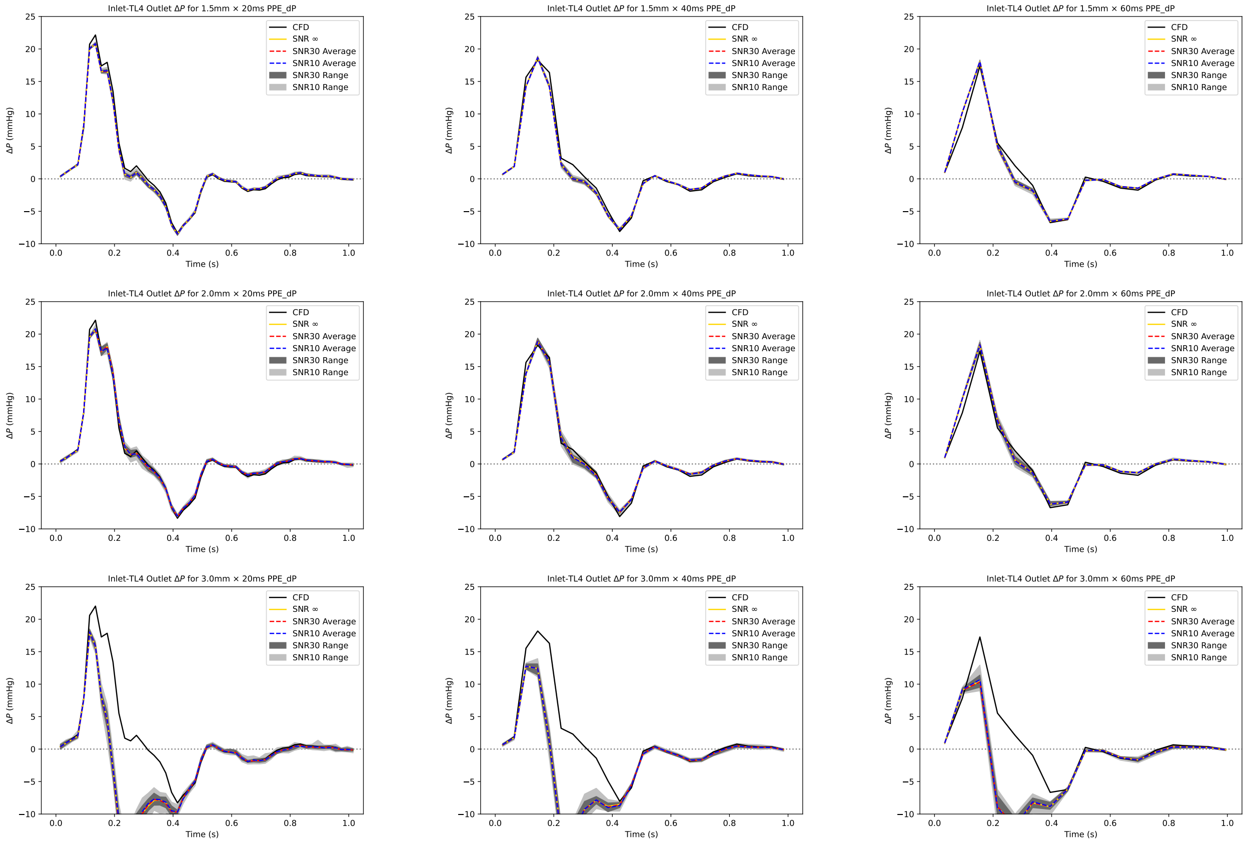
Inlet-TL4 Outlet ΔP for 1.5mm × 20ms PPE_dP
Inlet-TL4 Outlet ΔP for 1.5mm × 40ms PPE_dP
Inlet-TL4 Outlet ΔP for 1.5mm × 60ms PPE_dP
Inlet-TL4 Outlet ΔP for 2.0mm × 20ms PPE_dP
Inlet-TL4 Outlet ΔP for 2.0mm × 40ms PPE_dP
Inlet-TL4 Outlet ΔP for 2.0mm × 60ms PPE_dP
Inlet-TL4 Outlet ΔP for 3.0mm × 20ms PPE_dP
Inlet-TL4 Outlet ΔP for 3.0mm × 40ms PPE_dP
Inlet-TL4 Outlet ΔP for 3.0mm × 60ms PPE_dP
CFD
SNR ∞
SNR30 Average
SNR10 Average
SNR30 Range
SNR10 Range
ΔP (mmHg)
Time (s)

1.7 Deviation Tables

Average Deviation for vWERP, SNR30

dx

1.5mm × 20ms

	Avg Sigma Error	Avg Max Error
FL	2.25%	1.22%
TL	1.96%	1.67%

1.5mm × 40ms

	Avg Sigma Error	Avg Max Error
FL	1.45%	0.97%
TL	1.58%	1.36%

1.5mm × 60ms

	Avg Sigma Error	Avg Max Error
FL	1.27%	0.76%
TL	1.48%	1.28%

2.0mm × 20ms

	Avg Sigma Error	Avg Max Error
FL	3.21%	1.67%
TL	2.58%	2.50%

2.0mm × 40ms

	Avg Sigma Error	Avg Max Error
FL	1.83%	1.13%
TL	2.11%	2.13%

2.0mm × 60ms

	Avg Sigma Error	Avg Max Error
FL	1.49%	0.87%
TL	2.04%	1.82%

3.0mm × 20ms

	Avg Sigma Error	Avg Max Error
FL	17.52%	9.30%
TL	8.83%	10.00%

3.0mm × 40ms

	Avg Sigma Error	Avg Max Error
FL	10.29%	6.11%
TL	7.45%	9.45%

3.0mm × 60ms

	Avg Sigma Error	Avg Max Error
FL	8.67%	5.34%
TL	6.70%	7.83%

dt

Average Deviation for vWERP, SNR10

dx

1.5mm × 20ms

	Avg Sigma Error	Avg Max Error
FL	2.88%	2.07%
TL	3.37%	2.79%

1.5mm × 40ms

	Avg Sigma Error	Avg Max Error
FL	2.96%	1.71%
TL	2.76%	2.34%

1.5mm × 60ms

	Avg Sigma Error	Avg Max Error
FL	2.71%	1.50%
TL	2.60%	2.18%

2.0mm × 20ms

	Avg Sigma Error	Avg Max Error
FL	5.67%	3.00%
TL	4.41%	4.09%

2.0mm × 40ms

	Avg Sigma Error	Avg Max Error
FL	3.39%	2.05%
TL	3.79%	3.70%

2.0mm × 60ms

	Avg Sigma Error	Avg Max Error
FL	2.81%	1.64%
TL	3.70%	3.25%

3.0mm × 20ms

	Avg Sigma Error	Avg Max Error
FL	30.95%	16.55%
TL	16.00%	16.86%

3.0mm × 40ms

	Avg Sigma Error	Avg Max Error
FL	18.56%	11.43%
TL	12.95%	16.55%

3.0mm × 60ms

	Avg Sigma Error	Avg Max Error
FL	17.06%	10.70%
TL	12.12%	14.55%

dt

Average Deviation for STE_dP, SNR30

dx

1.5mm × 20ms

	Avg Sigma Error	Avg Max Error
FL	1.93%	0.97%
TL	1.90%	1.66%

1.5mm × 40ms

	Avg Sigma Error	Avg Max Error
FL	1.04%	0.62%
TL	1.49%	1.38%

1.5mm × 60ms

	Avg Sigma Error	Avg Max Error
FL	0.72%	0.45%
TL	1.40%	1.09%

2.0mm × 20ms

	Avg Sigma Error	Avg Max Error
FL	2.95%	1.50%
TL	2.51%	2.46%

2.0mm × 40ms

	Avg Sigma Error	Avg Max Error
FL	1.57%	0.90%
TL	2.09%	1.96%

2.0mm × 60ms

	Avg Sigma Error	Avg Max Error
FL	1.19%	0.73%
TL	1.99%	1.74%

3.0mm × 20ms

	Avg Sigma Error	Avg Max Error
FL	4.90%	2.46%
TL	6.91%	9.04%

3.0mm × 40ms

	Avg Sigma Error	Avg Max Error
FL	2.60%	1.56%
TL	6.35%	10.16%

3.0mm × 60ms

	Avg Sigma Error	Avg Max Error
FL	1.95%	1.10%
TL	5.95%	8.77%

dt

Average Deviation for STE_dP, SNR10

dx

1.5mm × 20ms

	Avg Sigma Error	Avg Max Error
FL	3.34%	1.72%
TL	3.31%	2.90%

1.5mm × 40ms

	Avg Sigma Error	Avg Max Error
FL	1.81%	1.13%
TL	2.69%	2.37%

1.5mm × 60ms

	Avg Sigma Error	Avg Max Error
FL	1.45%	0.88%
TL	2.36%	1.84%

2.0mm × 20ms

	Avg Sigma Error	Avg Max Error
FL	4.87%	2.51%
TL	4.61%	4.32%

2.0mm × 40ms

	Avg Sigma Error	Avg Max Error
FL	2.71%	1.61%
TL	3.92%	3.84%

2.0mm × 60ms

	Avg Sigma Error	Avg Max Error
FL	2.16%	1.22%
TL	3.49%	3.16%

3.0mm × 20ms

	Avg Sigma Error	Avg Max Error
FL	8.45%	4.44%
TL	12.27%	16.28%

3.0mm × 40ms

	Avg Sigma Error	Avg Max Error
FL	4.65%	2.79%
TL	11.49%	18.03%

3.0mm × 60ms

	Avg Sigma Error	Avg Max Error
FL	3.39%	2.05%
TL	10.56%	16.22%

dt

Average Deviation for PPE_dP, SNR30

1.5mm × 20ms

	Avg Sigma Error	Avg Max Error
FL	1.68%	0.85%
TL	1.72%	1.49%

1.5mm × 40ms

	Avg Sigma Error	Avg Max Error
FL	0.92%	0.57%
TL	1.31%	1.14%

1.5mm × 60ms

	Avg Sigma Error	Avg Max Error
FL	0.72%	0.46%
TL	1.21%	1.02%

2.0mm × 20ms

	Avg Sigma Error	Avg Max Error
FL	2.34%	1.27%
TL	2.82%	2.67%

2.0mm × 40ms

	Avg Sigma Error	Avg Max Error
FL	1.27%	0.84%
TL	2.42%	2.29%

2.0mm × 60ms

	Avg Sigma Error	Avg Max Error
FL	1.18%	0.78%
TL	2.23%	1.97%

3.0mm × 20ms

	Avg Sigma Error	Avg Max Error
FL	4.09%	2.28%
TL	6.41%	9.62%

3.0mm × 40ms

	Avg Sigma Error	Avg Max Error
FL	2.25%	1.51%
TL	5.50%	9.14%

3.0mm × 60ms

	Avg Sigma Error	Avg Max Error
FL	1.93%	1.23%
TL	5.68%	9.46%

dx

dt

Average Deviation for PPE_dP, SNR10

1.5mm × 20ms

	Avg Sigma Error	Avg Max Error
FL	2.86%	1.43%
TL	2.94%	2.47%

1.5mm × 40ms

	Avg Sigma Error	Avg Max Error
FL	1.69%	1.07%
TL	2.28%	1.87%

1.5mm × 60ms

	Avg Sigma Error	Avg Max Error
FL	1.53%	0.95%
TL	2.27%	1.88%

2.0mm × 20ms

	Avg Sigma Error	Avg Max Error
FL	4.11%	2.24%
TL	4.69%	4.06%

2.0mm × 40ms

	Avg Sigma Error	Avg Max Error
FL	2.43%	1.59%
TL	4.05%	3.94%

2.0mm × 60ms

	Avg Sigma Error	Avg Max Error
FL	2.02%	1.40%
TL	3.93%	3.47%

3.0mm × 20ms

	Avg Sigma Error	Avg Max Error
FL	6.87%	3.75%
TL	10.84%	15.58%

3.0mm × 40ms

	Avg Sigma Error	Avg Max Error
FL	3.80%	2.52%
TL	9.73%	15.72%

3.0mm × 60ms

	Avg Sigma Error	Avg Max Error
FL	2.91%	2.05%
TL	10.30%	16.89%

dx

dt

2 In Vitro Model

2.1 Pressure Drop Correlation Plots

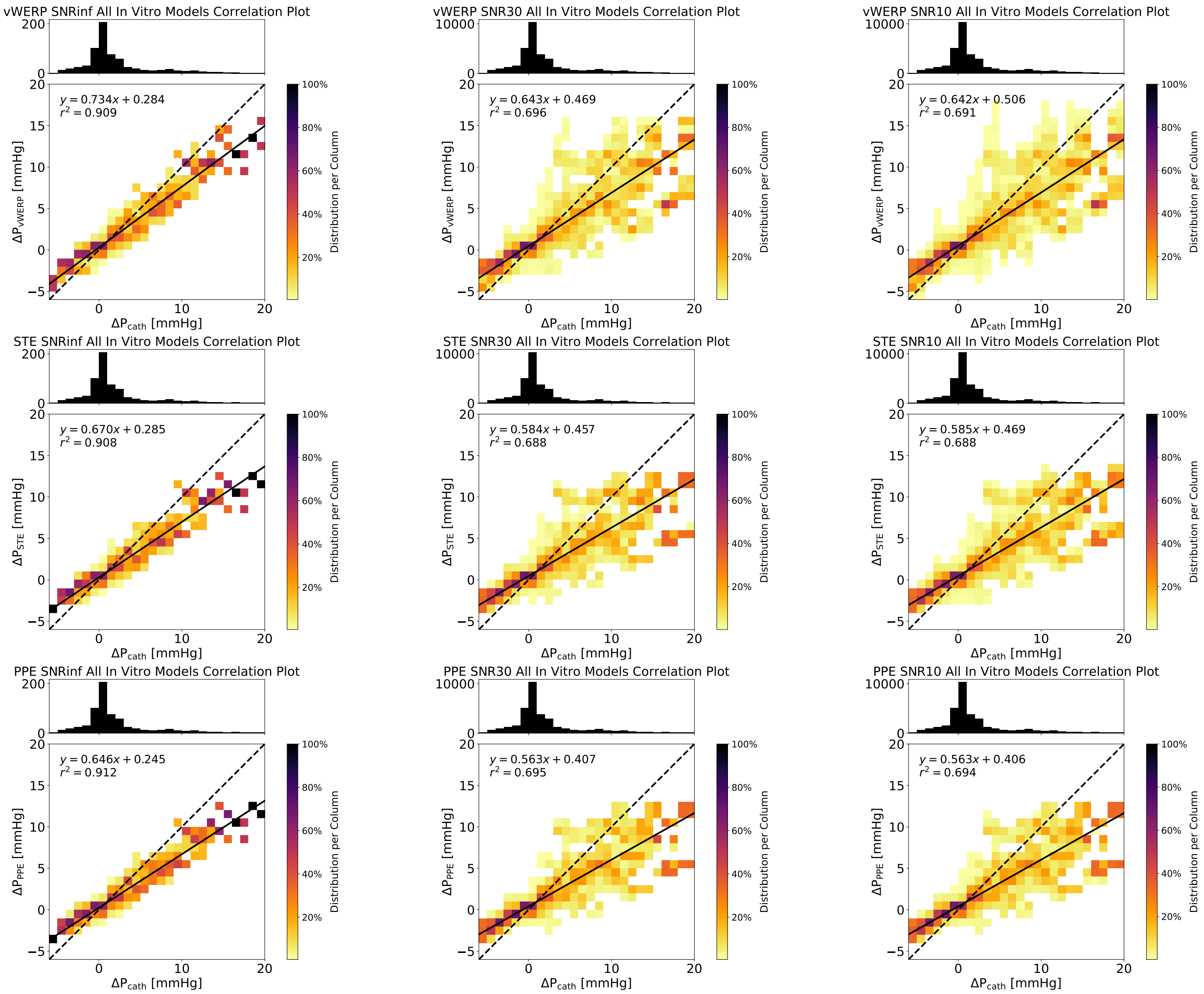

2.2 Deviation Tables

Average Deviation for vWERP

Model

TBAD_OR SNR30

	Avg Sigma Error	Avg Max Error
FL	1.30%	1.06%
TL	2.13%	1.46%
Outlet	2.09%	1.51%

TBAD_OR SNR10

	Avg Sigma Error	Avg Max Error
FL	5.00%	3.60%
TL	8.31%	5.22%
Outlet	7.27%	5.25%

TBAD_ENT SNR30

	Avg Sigma Error	Avg Max Error
FL	1.52%	1.46%
TL	2.95%	2.27%
Outlet	2.38%	2.50%

TBAD_ENT SNR10

	Avg Sigma Error	Avg Max Error
FL	5.41%	4.73%
TL	12.69%	8.90%
Outlet	8.55%	8.25%

TBAD_EXT SNR30

	Avg Sigma Error	Avg Max Error
FL	0.72%	0.57%
TL	1.49%	1.16%
Outlet	0.61%	0.60%

TBAD_EXT SNR10

	Avg Sigma Error	Avg Max Error
FL	2.58%	1.95%
TL	5.03%	3.71%
Outlet	2.02%	1.94%

SNR

Average Deviation for STE

Model / SNR

TBAD_OR SNR30

	Avg Sigma Error	Avg Max Error
FL	0.97%	0.71%
TL	1.83%	1.26%
Outlet	0.87%	0.62%

TBAD_OR SNR10

	Avg Sigma Error	Avg Max Error
FL	3.58%	2.44%
TL	6.97%	4.44%
Outlet	3.29%	2.22%

TBAD_ENT SNR30

	Avg Sigma Error	Avg Max Error
FL	1.20%	1.22%
TL	2.76%	2.15%
Outlet	1.22%	1.16%

TBAD_ENT SNR10

	Avg Sigma Error	Avg Max Error
FL	4.42%	3.83%
TL	10.80%	7.72%
Outlet	4.73%	4.15%

TBAD_EXT SNR30

	Avg Sigma Error	Avg Max Error
FL	1.37%	1.08%
TL	2.73%	2.10%
Outlet	1.18%	1.12%

TBAD_EXT SNR10

	Avg Sigma Error	Avg Max Error
FL	5.59%	3.88%
TL	10.58%	7.40%
Outlet	4.43%	3.88%

Average Deviation for PPE

Model / SNR

TBAD_OR SNR30

	Avg Sigma Error	Avg Max Error
FL	0.48%	0.36%
TL	0.95%	0.69%
Outlet	0.42%	0.30%

TBAD_OR SNR10

	Avg Sigma Error	Avg Max Error
FL	1.54%	1.16%
TL	3.08%	2.14%
Outlet	1.38%	0.97%

TBAD_ENT SNR30

	Avg Sigma Error	Avg Max Error
FL	0.57%	0.60%
TL	1.38%	1.11%
Outlet	0.58%	0.57%

TBAD_ENT SNR10

	Avg Sigma Error	Avg Max Error
FL	1.81%	1.85%
TL	4.49%	3.50%
Outlet	1.88%	1.75%

TBAD_EXT SNR30

	Avg Sigma Error	Avg Max Error
FL	0.69%	0.55%
TL	1.35%	1.12%
Outlet	0.54%	0.54%

TBAD_EXT SNR10

	Avg Sigma Error	Avg Max Error
FL	2.27%	1.77%
TL	4.42%	3.55%
Outlet	1.79%	1.69%

2.3 Pressure Drop Figures

2.3.1 *v*WERP

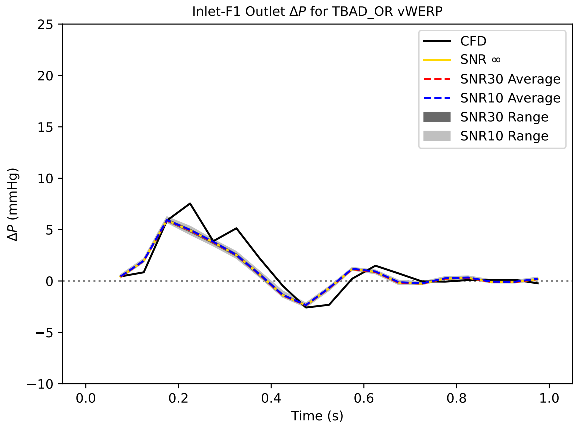

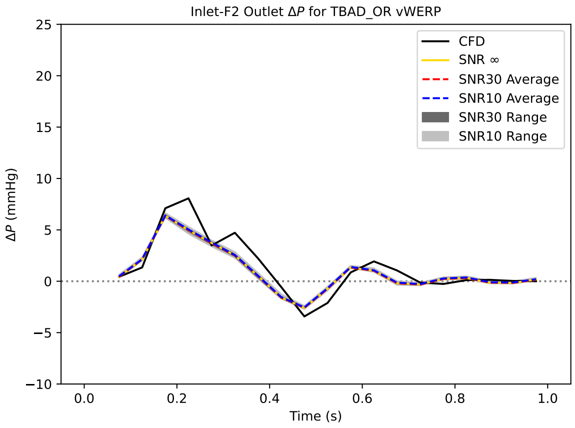

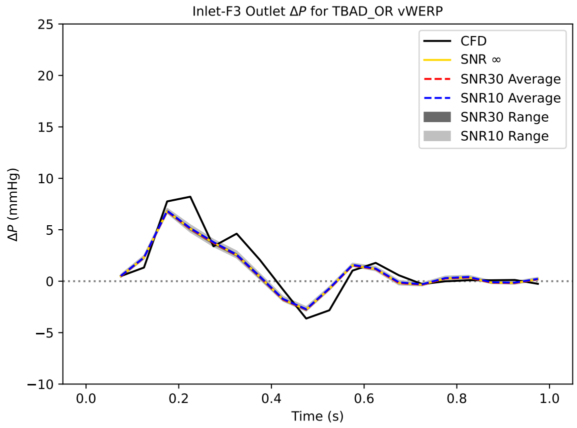

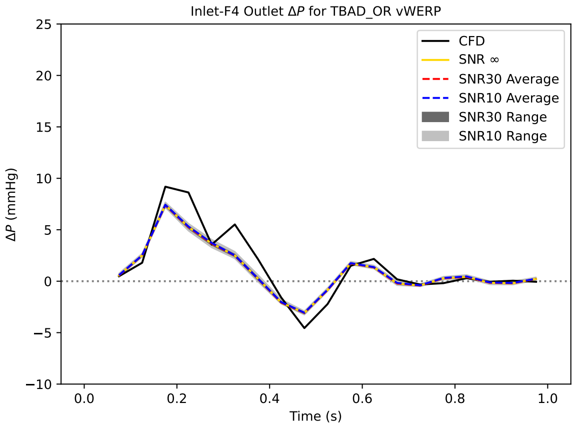

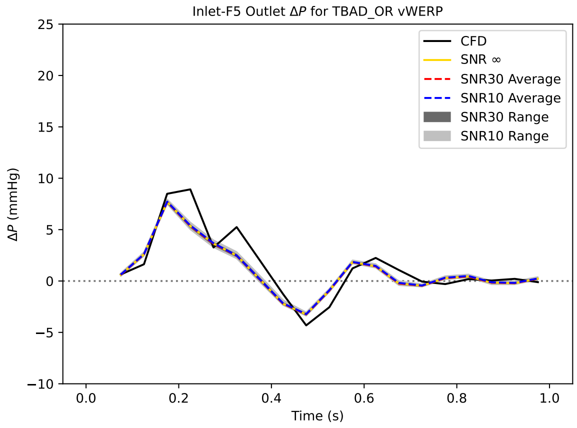

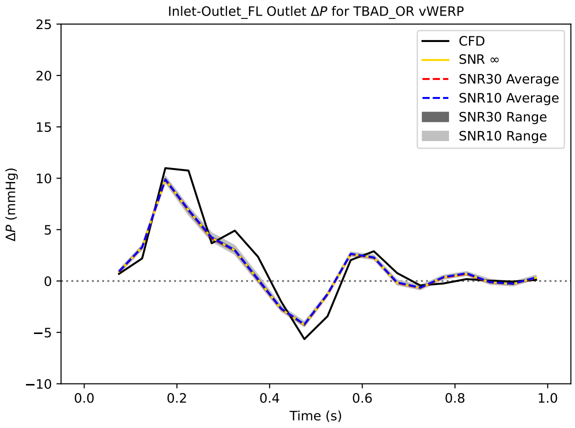

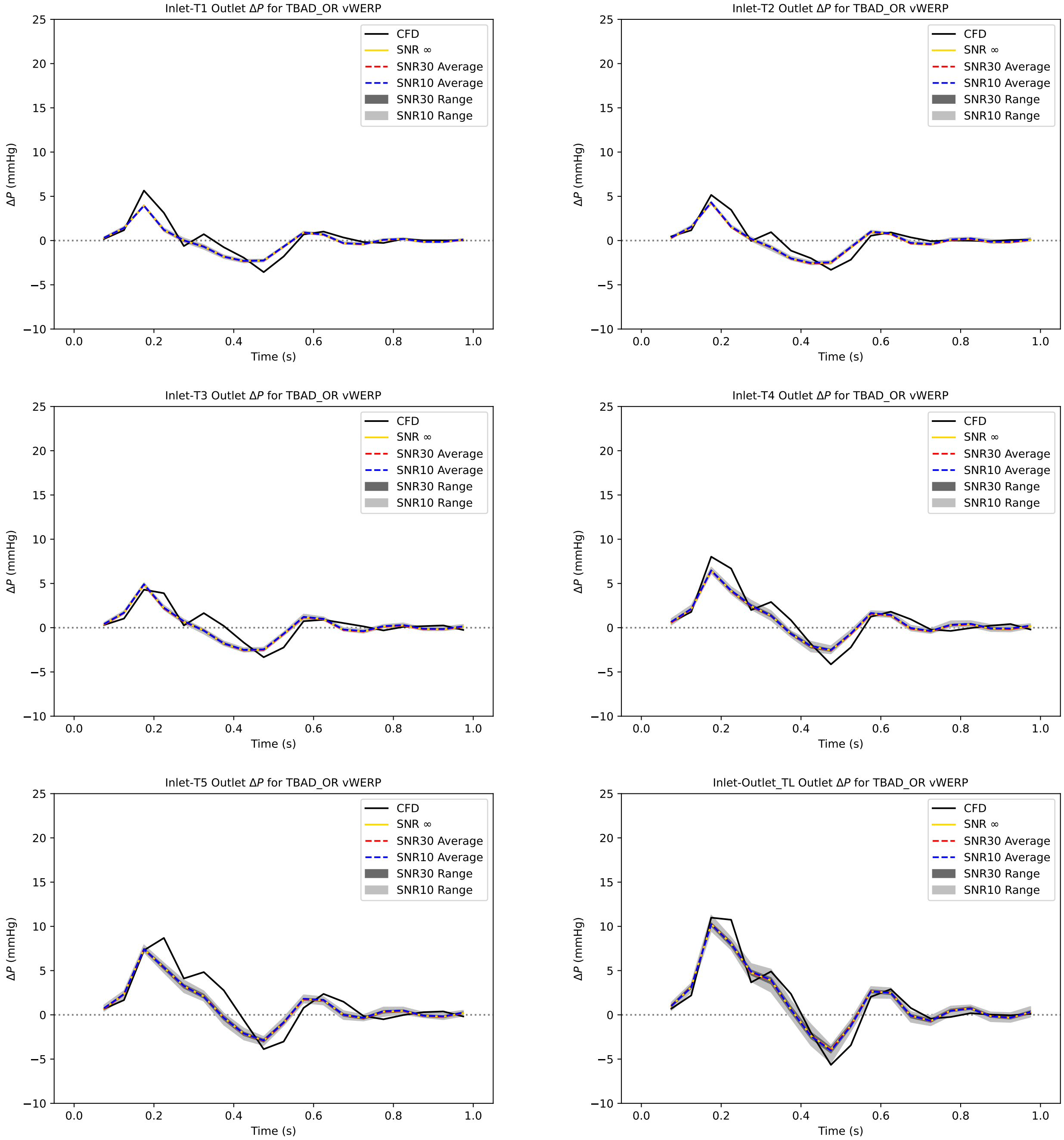
Inlet-T1 Outlet ΔP for TBAD_OR vWERP
Inlet-T2 Outlet ΔP for TBAD_OR vWERP
Inlet-T3 Outlet ΔP for TBAD_OR vWERP
Inlet-T4 Outlet ΔP for TBAD_OR vWERP
Inlet-T5 Outlet ΔP for TBAD_OR vWERP
Inlet-Outlet_TL Outlet ΔP for TBAD_OR vWERP
CFD
SNR ∞
SNR30 Average
SNR10 Average
SNR30 Range
SNR10 Range
ΔP (mmHg)
Time (s)

2.3.2 STE

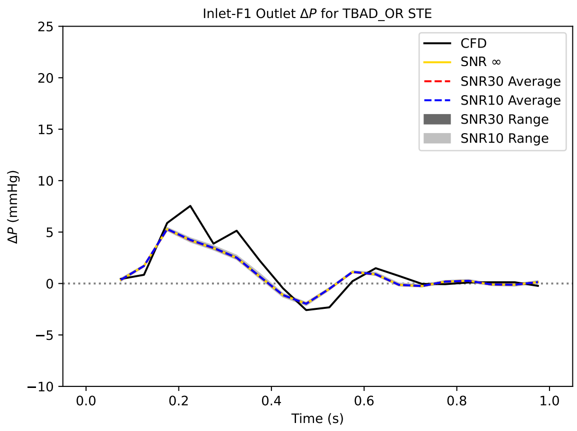
Inlet-F1 Outlet ΔP for TBAD_OR STE
CFD
SNR ∞
SNR30 Average
SNR10 Average
SNR30 Range
SNR10 Range
ΔP (mmHg)
Time (s)

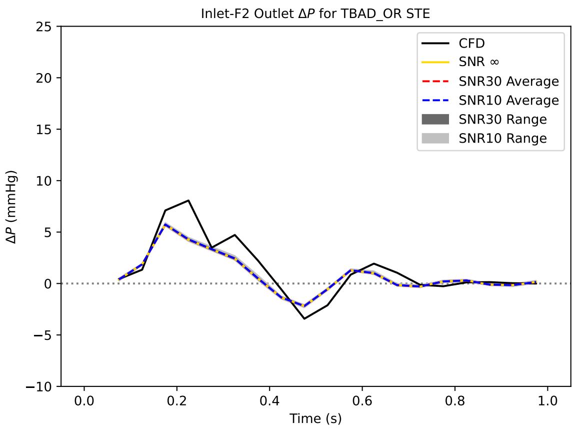
Inlet-F2 Outlet ΔP for TBAD_OR STE
CFD
SNR ∞
SNR30 Average
SNR10 Average
SNR30 Range
SNR10 Range
ΔP (mmHg)
Time (s)

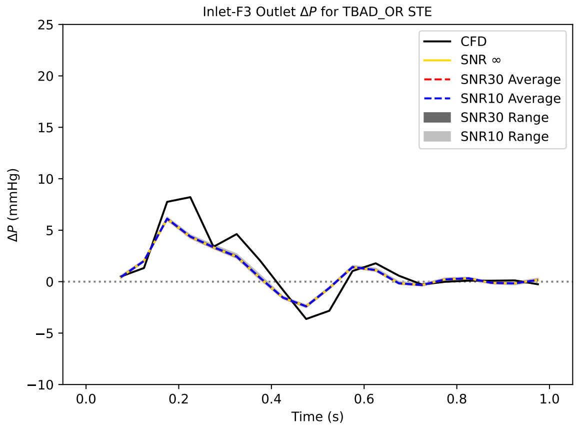
Inlet-F3 Outlet ΔP for TBAD_OR STE
CFD
SNR ∞
SNR30 Average
SNR10 Average
SNR30 Range
SNR10 Range
ΔP (mmHg)
Time (s)

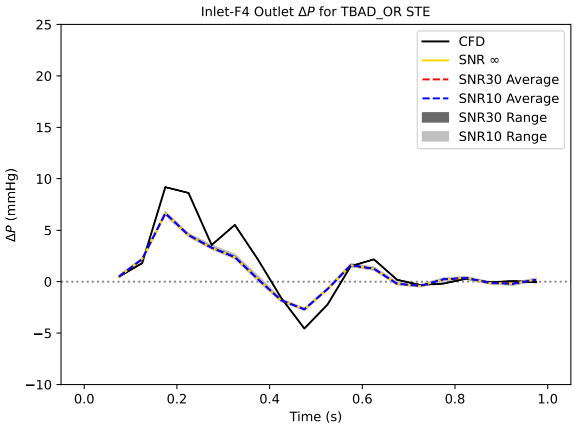
Inlet-F4 Outlet ΔP for TBAD_OR STE
CFD
SNR ∞
SNR30 Average
SNR10 Average
SNR30 Range
SNR10 Range
ΔP (mmHg)
Time (s)

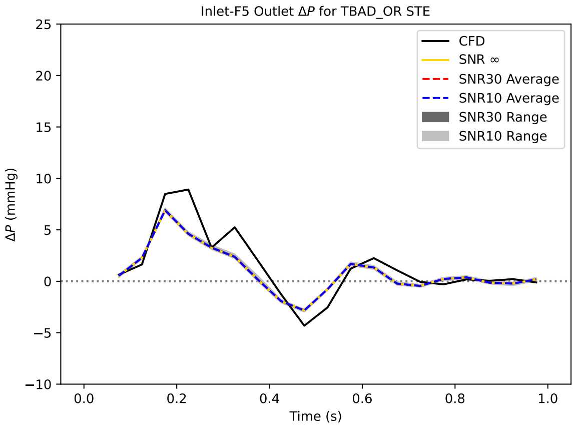
Inlet-F5 Outlet ΔP for TBAD_OR STE
CFD
SNR ∞
SNR30 Average
SNR10 Average
SNR30 Range
SNR10 Range
ΔP (mmHg)
Time (s)

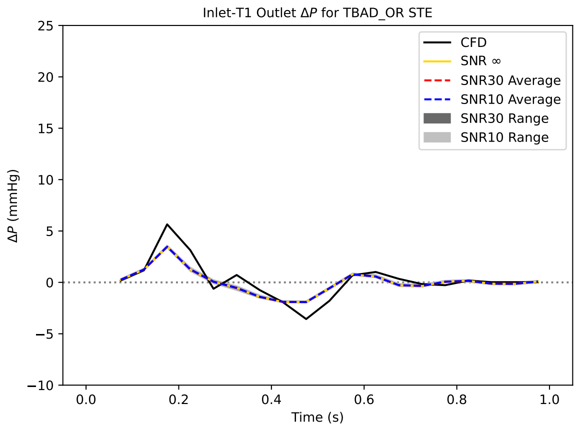
Inlet-T1 Outlet ΔP for TBAD_OR STE
CFD
SNR ∞
SNR30 Average
SNR10 Average
SNR30 Range
SNR10 Range
ΔP (mmHg)
Time (s)

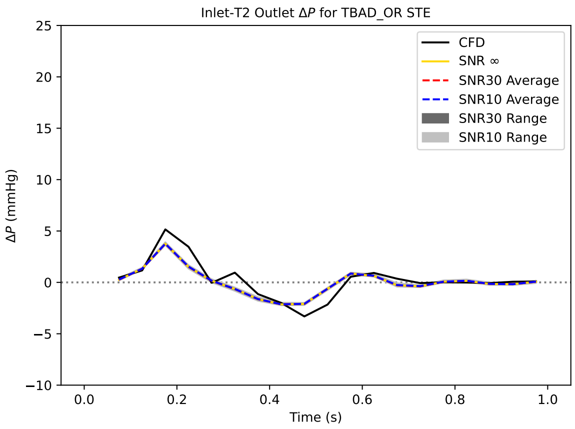
Inlet-T2 Outlet ΔP for TBAD_OR STE
CFD
SNR ∞
SNR30 Average
SNR10 Average
SNR30 Range
SNR10 Range
ΔP (mmHg)
Time (s)

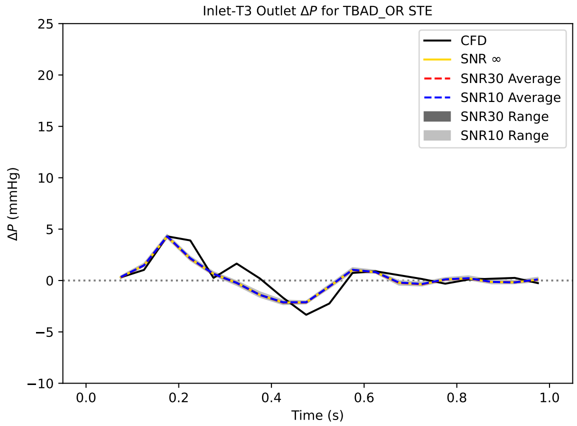
Inlet-T3 Outlet ΔP for TBAD_OR STE
CFD
SNR ∞
SNR30 Average
SNR10 Average
SNR30 Range
SNR10 Range
ΔP (mmHg)
Time (s)

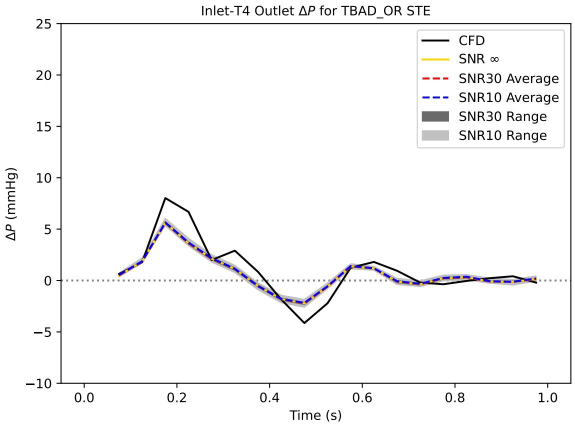
Inlet-T4 Outlet ΔP for TBAD_OR STE
CFD
SNR ∞
SNR30 Average
SNR10 Average
SNR30 Range
SNR10 Range
ΔP (mmHg)
Time (s)

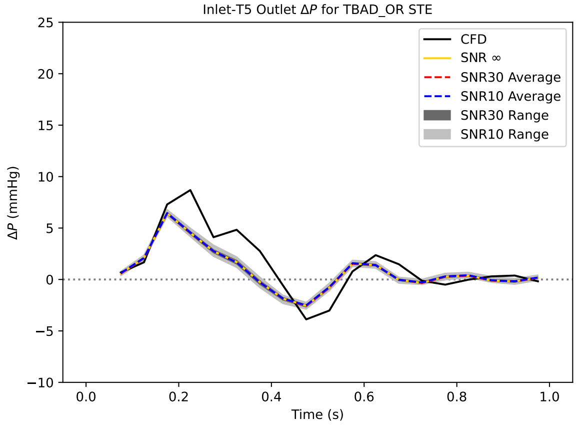
Inlet-T5 Outlet ΔP for TBAD_OR STE
CFD
SNR ∞
SNR30 Average
SNR10 Average
SNR30 Range
SNR10 Range
ΔP (mmHg)
Time (s)

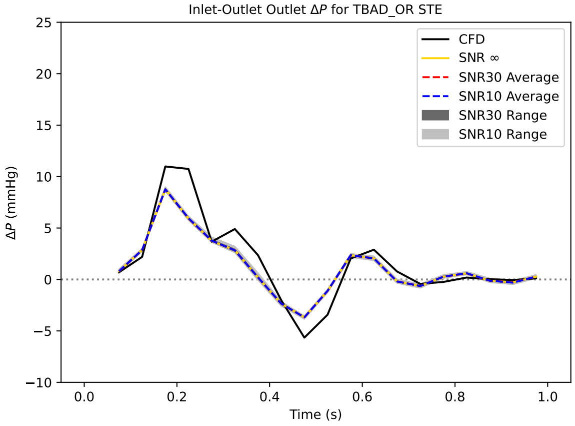
Inlet-Outlet Outlet ΔP for TBAD_OR STE
CFD
SNR ∞
SNR30 Average
SNR10 Average
SNR30 Range
SNR10 Range
ΔP (mmHg)
Time (s)

2.3.3 PPE

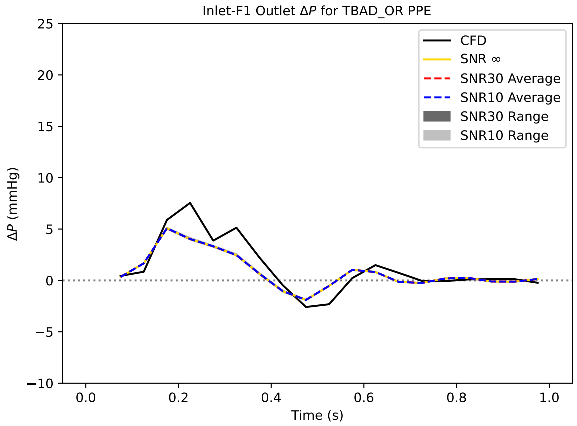

Inlet-F1 Outlet ΔP for TBAD_OR PPE
CFD
SNR ∞
SNR30 Average
SNR10 Average
SNR30 Range
SNR10 Range
ΔP (mmHg)
Time (s)

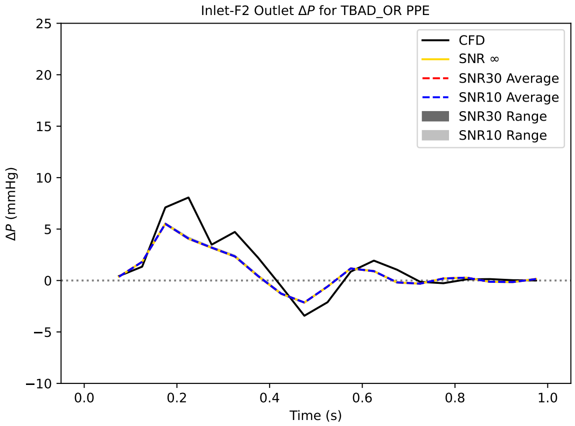

Inlet-F2 Outlet ΔP for TBAD_OR PPE
CFD
SNR ∞
SNR30 Average
SNR10 Average
SNR30 Range
SNR10 Range
ΔP (mmHg)
Time (s)

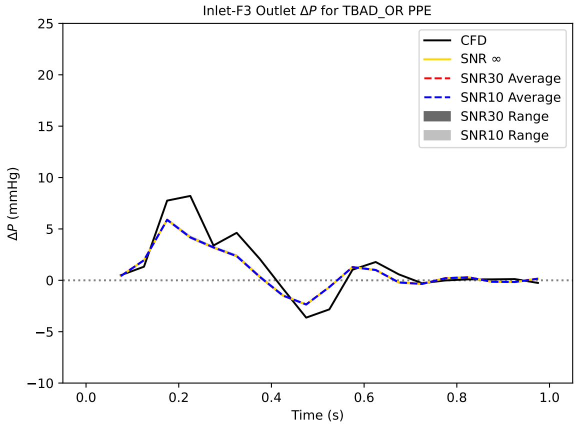

Inlet-F3 Outlet ΔP for TBAD_OR PPE
CFD
SNR ∞
SNR30 Average
SNR10 Average
SNR30 Range
SNR10 Range
ΔP (mmHg)
Time (s)

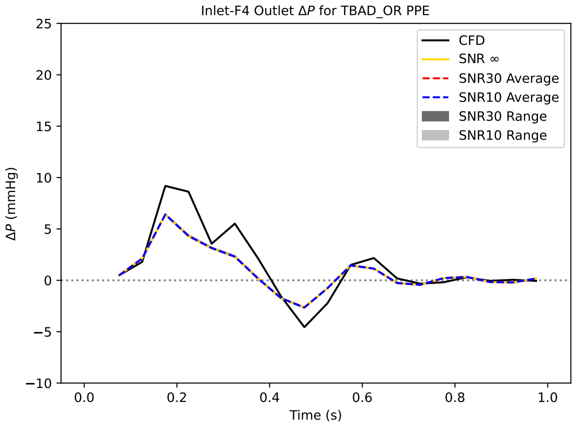

Inlet-F4 Outlet ΔP for TBAD_OR PPE
CFD
SNR ∞
SNR30 Average
SNR10 Average
SNR30 Range
SNR10 Range
ΔP (mmHg)
Time (s)

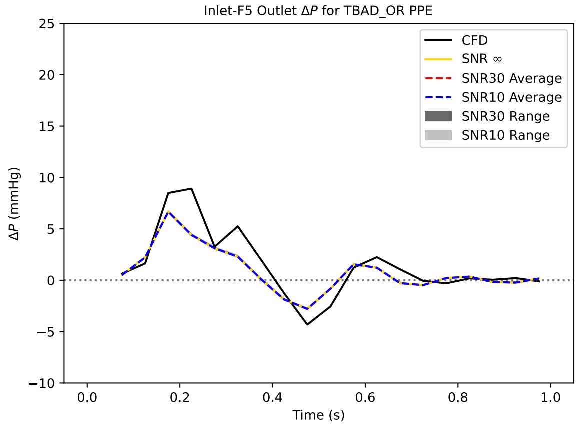

Inlet-F5 Outlet ΔP for TBAD_OR PPE
CFD
SNR ∞
SNR30 Average
SNR10 Average
SNR30 Range
SNR10 Range
ΔP (mmHg)
Time (s)

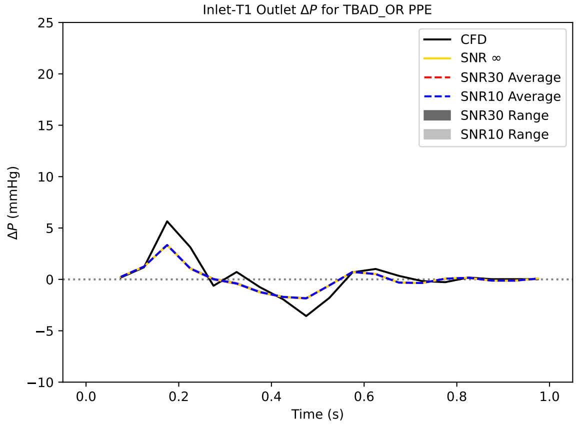
Inlet-T1 Outlet ΔP for TBAD_OR PPE
CFD
SNR ∞
SNR30 Average
SNR10 Average
SNR30 Range
SNR10 Range
ΔP (mmHg)
Time (s)

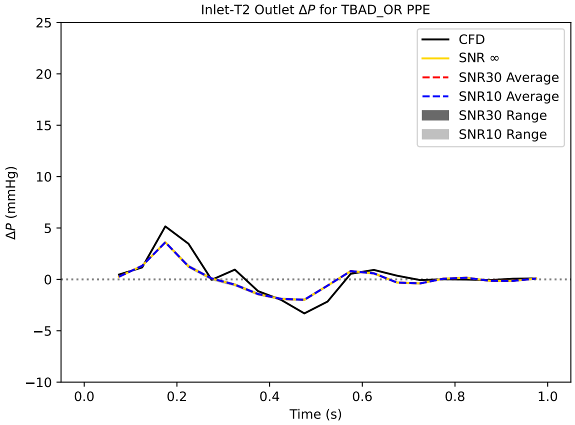
Inlet-T2 Outlet ΔP for TBAD_OR PPE
CFD
SNR ∞
SNR30 Average
SNR10 Average
SNR30 Range
SNR10 Range
ΔP (mmHg)
Time (s)

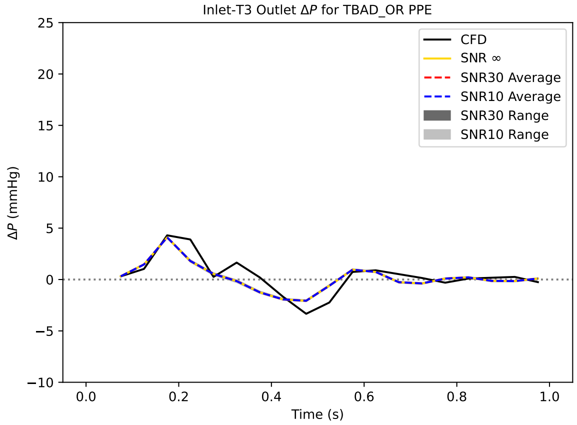
Inlet-T3 Outlet ΔP for TBAD_OR PPE
CFD
SNR ∞
SNR30 Average
SNR10 Average
SNR30 Range
SNR10 Range
ΔP (mmHg)
Time (s)

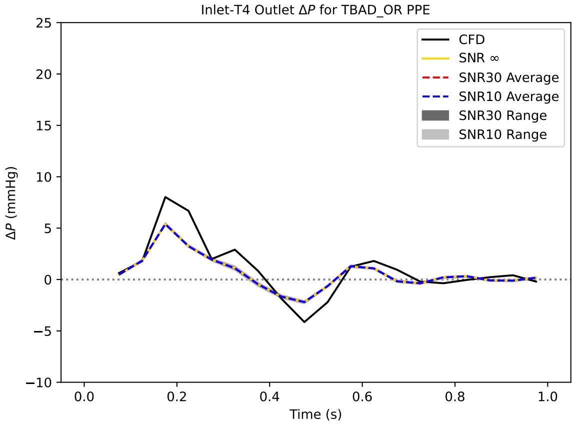
Inlet-T4 Outlet ΔP for TBAD_OR PPE
CFD
SNR ∞
SNR30 Average
SNR10 Average
SNR30 Range
SNR10 Range
ΔP (mmHg)
Time (s)

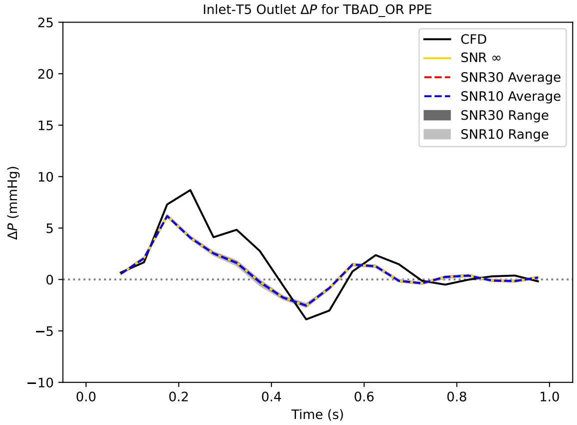
Inlet-T5 Outlet ΔP for TBAD_OR PPE
CFD
SNR ∞
SNR30 Average
SNR10 Average
SNR30 Range
SNR10 Range
ΔP (mmHg)
Time (s)

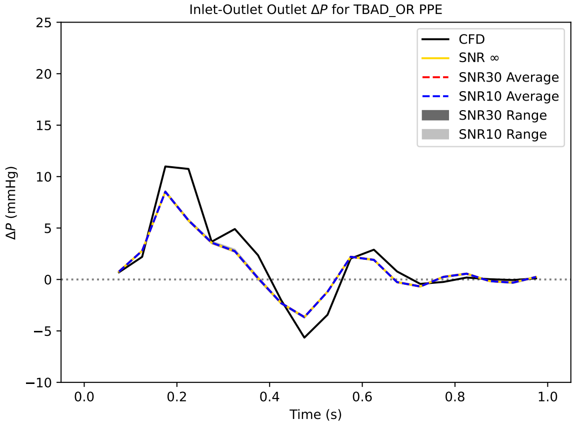
Inlet-Outlet Outlet ΔP for TBAD_OR PPE
CFD
SNR ∞
SNR30 Average
SNR10 Average
SNR30 Range
SNR10 Range
ΔP (mmHg)
Time (s)

3 Patient Cases

3.1 Individual Patients

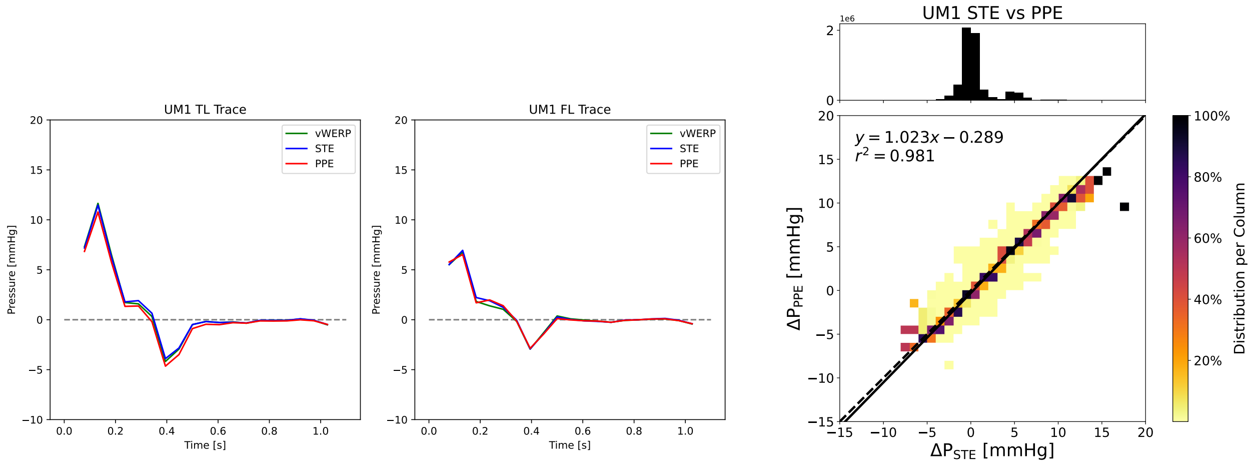

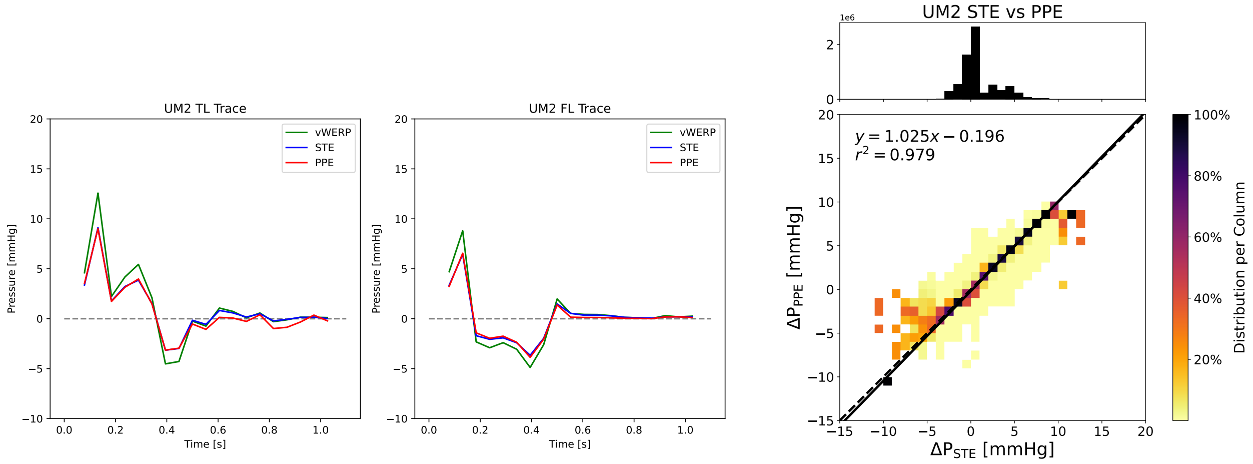

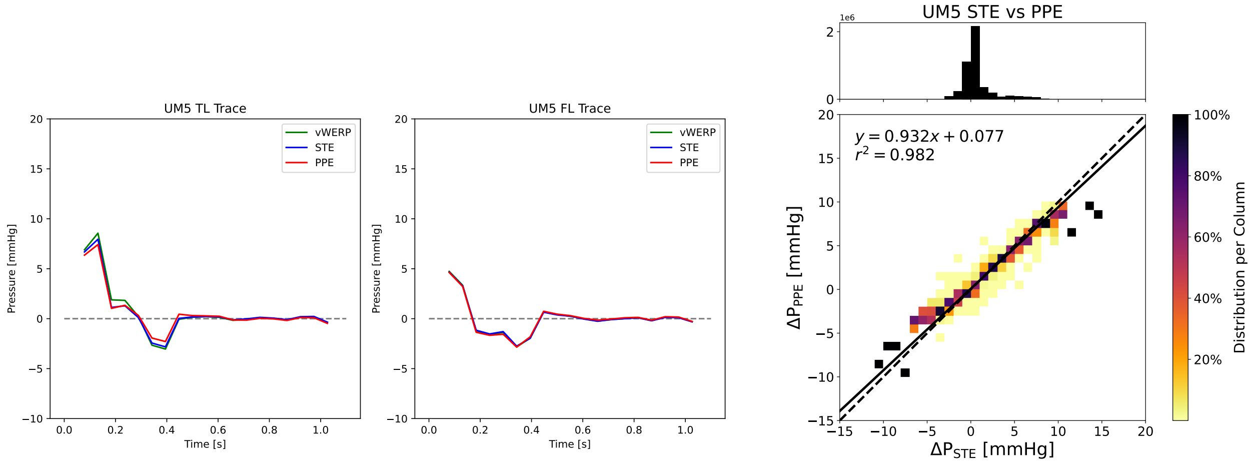

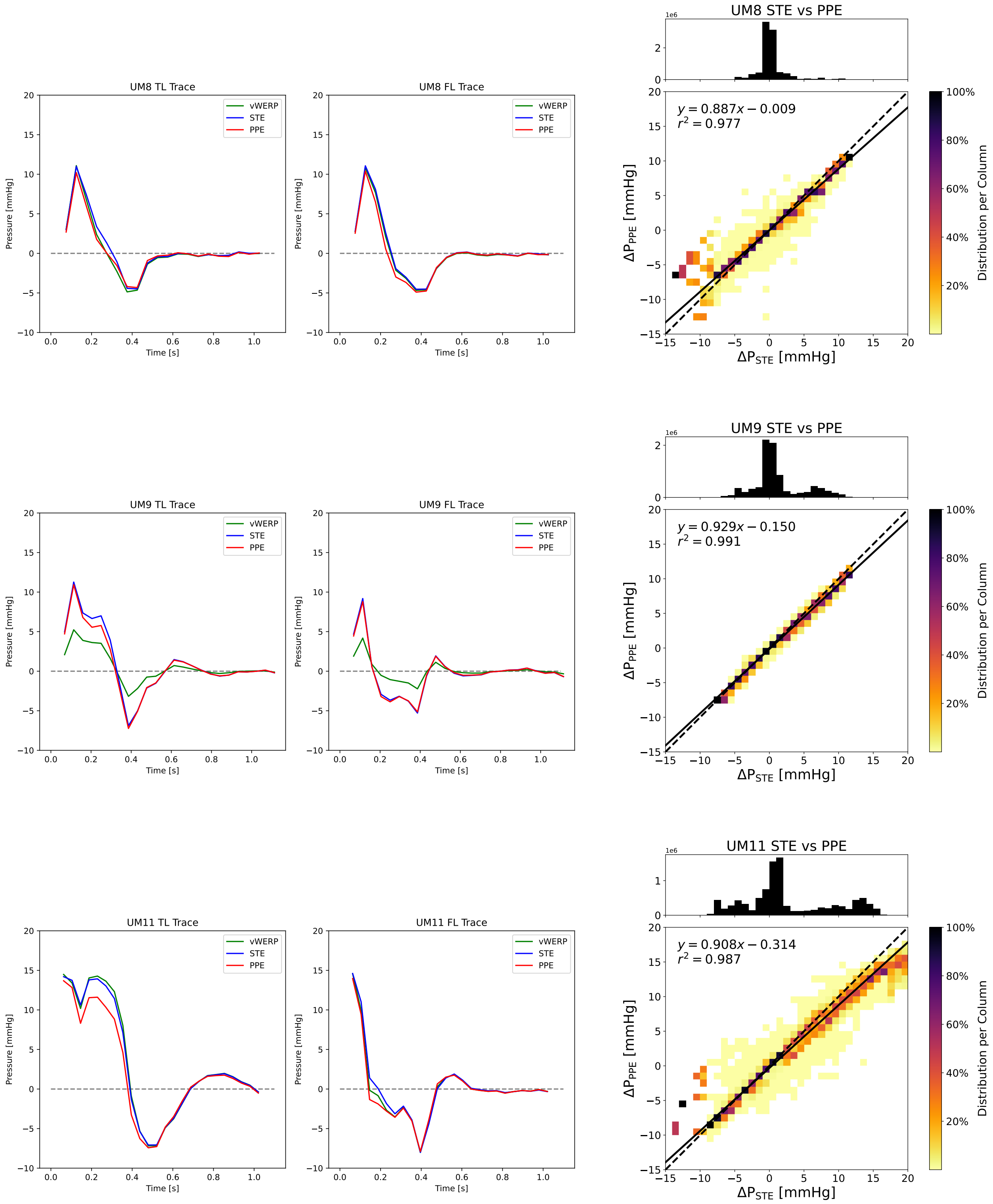
UM8 TL Trace
UM8 FL Trace
UM8 STE vs PPE
$y = 0.887x - 0.009$
$r^2 = 0.977$
UM9 TL Trace
UM9 FL Trace
UM9 STE vs PPE
$y = 0.929x - 0.150$
$r^2 = 0.991$
UM11 TL Trace
UM11 FL Trace
UM11 STE vs PPE
$y = 0.908x - 0.314$
$r^2 = 0.987$
vWERP
STE
PPE
Pressure [mmHg]
Time [s]
ΔP_{PPE} [mmHg]
ΔP_{STE} [mmHg]
Distribution per Column
1e6

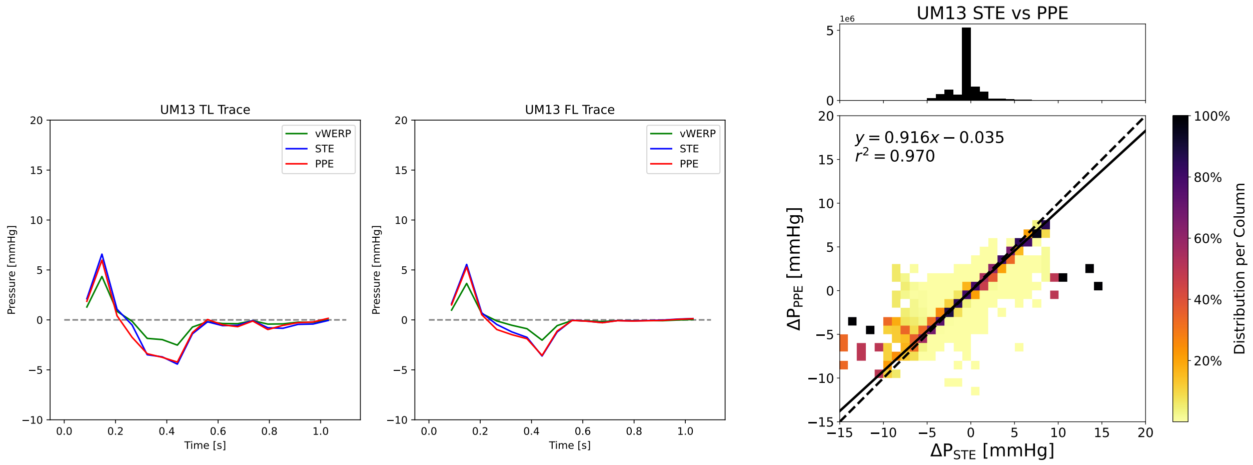

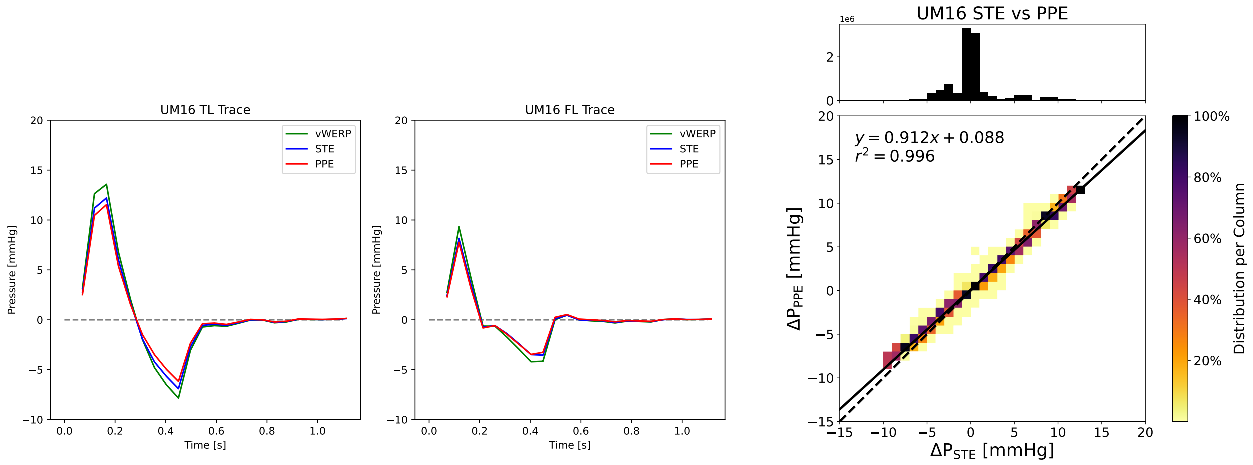

3.2 Pressure Drop Correlation Plots

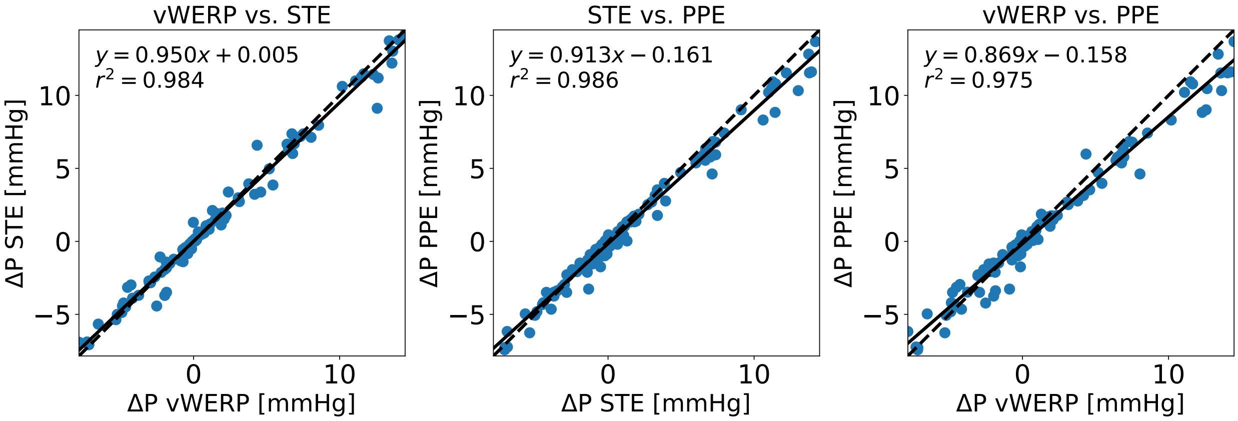

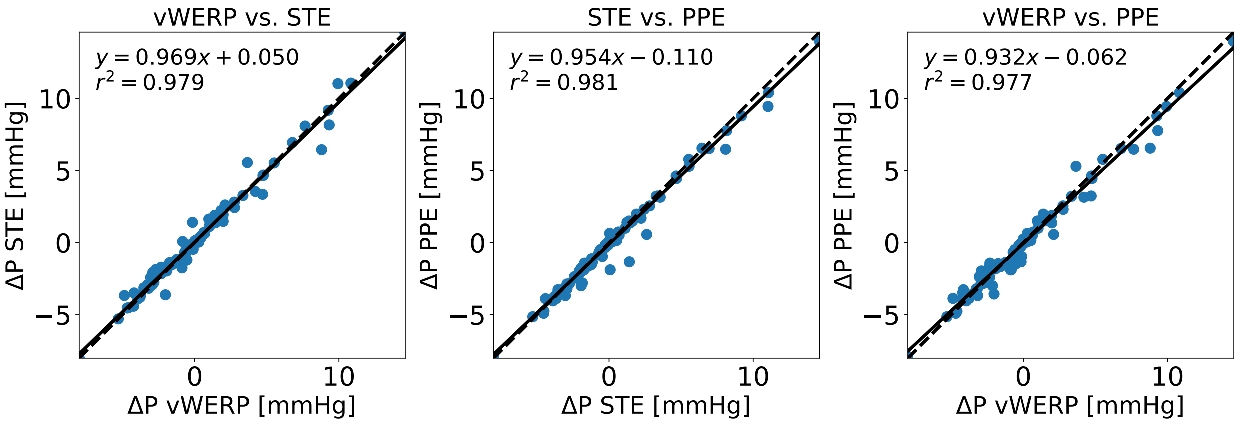
vWERP vs. STE
$y = 0.969x + 0.050$
$r^2 = 0.979$
ΔP STE [mmHg]
ΔP vWERP [mmHg]
STE vs. PPE
$y = 0.954x - 0.110$
$r^2 = 0.981$
ΔP PPE [mmHg]
ΔP STE [mmHg]
vWERP vs. PPE
$y = 0.932x - 0.062$
$r^2 = 0.977$
ΔP PPE [mmHg]
ΔP vWERP [mmHg]
